# Receptor transfer between immune cells by autoantibody-enhanced, CD32-driven trogocytosis is hijacked by HIV-1 to infect resting CD4 T cells

**DOI:** 10.1016/j.xcrm.2024.101483

**Published:** 2024-04-04

**Authors:** Manuel Albanese, Hong-Ru Chen, Madeleine Gapp, Maximilian Muenchhoff, Hsiu-Hui Yang, David Peterhoff, Katja Hoffmann, Qianhao Xiao, Adrian Ruhle, Ina Ambiel, Stephanie Schneider, Ernesto Mejías-Pérez, Marcel Stern, Paul R. Wratil, Katharina Hofmann, Laura Amann, Linda Jocham, Thimo Fuchs, Alessandro F. Ulivi, Simon Besson-Girard, Simon Weidlich, Jochen Schneider, Christoph D. Spinner, Kathrin Sutter, Ulf Dittmer, Andreas Humpe, Philipp Baumeister, Andreas Wieser, Simon Rothenfusser, Johannes Bogner, Julia Roider, Percy Knolle, Hartmut Hengel, Ralf Wagner, Vibor Laketa, Oliver T. Fackler, Oliver T. Keppler

**Affiliations:** 1 Max von Pettenkofer Institute and Gene Center, Virology, National Reference Center for Retroviruses, Faculty of Medicine, LMU München, Munich, Germany; 2 Department for Clinical Sciences and Community Health (DISCCO), University of Milan, Milan, Italy; 3 German Centre for Infection Research (DZIF), Partner Site Munich, Munich, Germany; 4 Institute of Medical Microbiology and Hygiene, Molecular Microbiology (Virology), University of Regensburg, Regensburg, Germany; 5 Institute of Virology, University Medical Center, Albert-Ludwigs-University Freiburg, Freiburg, Germany; 6 Department of Infectious Diseases, Heidelberg University, Medical Faculty Heidelberg, Integrative Virology, Center for Integrative Infectious Disease Research (CIID), Heidelberg, Germany; 7 German Centre for Infection Research (DZIF), Partner Site Heidelberg, Heidelberg, Germany; 8 Max Planck Institute of Psychiatry, Munich, Germany; 9 Institute for Stroke and Dementia Research, University Hospital, LMU München, Munich, Germany; 10 Technical University of Munich, School of Medicine, University Hospital Rechts der Isar, Department of Internal Medicine II, Munich, Germany; 11 University Hospital Essen, University Duisburg-Essen, Institute for Virology and Institute for Translational HIV Research, Essen, Germany; 12 Department of Transfusion Medicine, Cell Therapeutics, and Hemostaseology, Department of Anesthesiology, University Hospital Munich, Munich, Germany; 13 Department of Otorhinolaryngology, Head and Neck Surgery, University Hospital, LMU München, Munich, Germany; 14 Max von Pettenkofer Institute, Medical Microbiology and Hospital Epidemiology, Faculty of Medicine, LMU München, Munich, Germany; 15 Division of Infectious Diseases and Tropical Medicine, University Hospital, LMU München, Munich, Germany; 16 Division of Clinical Pharmacology, University Hospital, LMU München and Unit Clinical Pharmacology (EKliP), Helmholtz Center for Environmental Health, Munich, Germany; 17 Division of Infectious Diseases, University Hospital, Medizinische Klinik und Poliklinik IV, LMU München, Munich, Germany; 18 Institute of Molecular Immunology and Experimental Oncology, School of Medicine, Technical University of Munich (TUM), Munich, Germany; 19 Department of Infectious Diseases, Heidelberg University, Medical Faculty Heidelberg, Virology, Center for Integrative Infectious Disease Research (CIID), Heidelberg, Germany

**Keywords:** immune cell communication, trogocytosis, CD32, autoantibodies, HIV reservoir, CRISPR-Cas9

## Abstract

Immune cell phenotyping frequently detects lineage-unrelated receptors. Here, we report that surface receptors can be transferred from primary macrophages to CD4 T cells and identify the Fcγ receptor CD32 as driver and cargo of this trogocytotic transfer. Filamentous CD32^+^ nanoprotrusions deposit distinct plasma membrane patches onto target T cells. Transferred receptors confer cell migration and adhesion properties, and macrophage-derived membrane patches render resting CD4 T cells susceptible to infection by serving as hotspots for HIV-1 binding. Antibodies that recognize T cell epitopes enhance CD32-mediated trogocytosis. Such autoreactive anti-HIV-1 envelope antibodies can be found in the blood of HIV-1 patients and, consistently, the percentage of CD32^+^ CD4 T cells is increased in their blood. This CD32-mediated, antigen-independent cell communication mode transiently expands the receptor repertoire and functionality of immune cells. HIV-1 hijacks this mechanism by triggering the generation of trogocytosis-promoting autoantibodies to gain access to immune cells critical to its persistence.

## Highlights

• CD32 is driver and cargo of trogocytic transfer via long cell protrusions
• Autoantibodies that bind to target cells boost CD32-mediated trogocytosis
• CD32-mediated trogocytosis increases CD4 T cells’ permissivity to HIV-1 infection
• HIV-1 patient sera are enriched in trogocytosis-enhancing autoantibodies

## Graphical abstract

**Figure:**
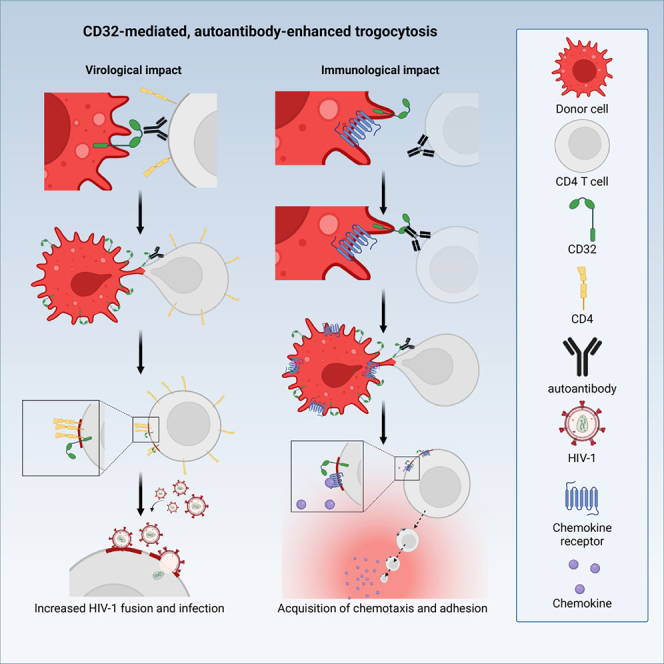

## Teaser

Albanese, Chen, Gapp et al. describe that the Fcγ receptor CD32 drives trogocytosis of membrane patches between immune cells and that this cell-cell transfer is markedly enhanced by autoantibodies. HIV-1 hijacks this mechanism by triggering the generation of trogocytosis-promoting autoantibodies to gain access to immune cells critical to its persistence.

## Introduction

The efficacy of specialized immune cells is regulated by differentiation into subsets with distinct differentiation and activation states that are characterized by specific protein markers exposed on their surface. This feature offers a convenient and widely used approach to define and study immune cell subsets by flow cytometry and microscopy.^1^ Cooperative immune cell functions rely on frequent intercellular communication such as the recognition of presented antigenic peptides, the triggering of signaling cascades by receptor-ligand interactions, or the release and capture of cytokines, often in the context of close physical cell-cell contacts (e.g., immune synapses). A plethora of studies describe atypical markers exposed on the surface of immune cells that is, however, not mirrored by gene expression of these receptors in these cells.^2^^,^^3^^,^^4^^,^^5^^,^^6^^,^^7^^,^^8^^,^^9^^,^^10^ Mechanisms proposed to explain this phenomenon include the exchange of membranes at antigen-dependent immune synapses by membrane stripping (trogocytosis),^11^^,^^12^ transfer of receptors or ligands via tunneling nanotubes,^13^ or the release and uptake of exosomal^13^^,^^14^ or ectosomal^15^^,^^16^ microvesicles.^17^ The unexpected detection of non-canonical surface markers can also reflect cells’ engagement in cell-cell contacts, resulting in cell doublets.^18^ These different modes of information exchange are typically triggered by specific receptor-ligand interactions in the context of antigen presentation, but their relative contribution to the overall membrane exchange between immune cells and the precise mechanisms regulating these modes of immune cell communication remain to be determined. To assess if such information exchange also occurs in the absence of specific triggers, we investigated receptor transfer between primary human macrophages and autologous CD4 T cells.

## Results

### Multiple receptors are transferred from human macrophages to autologous CD4 T cells in a contact-dependent manner

**Figure 1:**
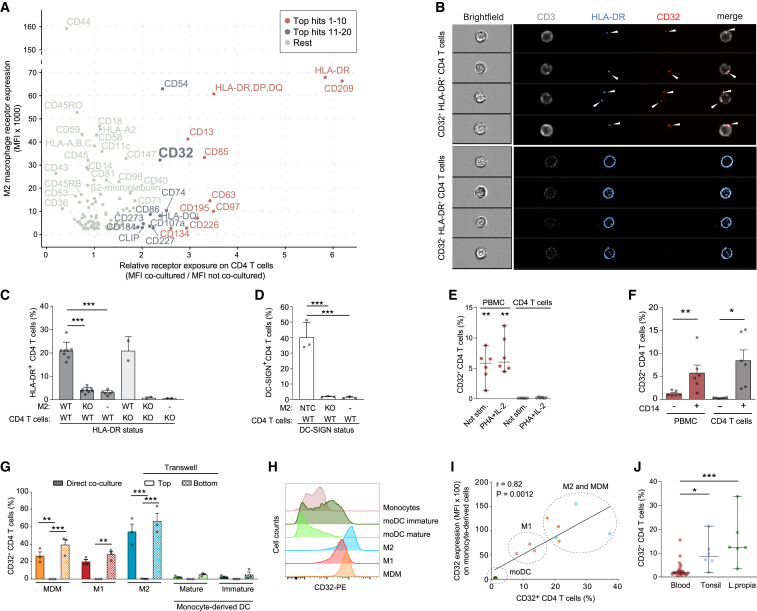
CD32 and other receptors are transferred from macrophages to co-cultured CD4 T cells (A) Screening for surface receptors transferred from autologous M2 macrophages (M2) to CD4 T cells following co-culture for 2 days. Receptors most highly transferred were categorized into “top hits 1–10” and “top hits 11–20,” respectively. x axis, mean of the mean fluorescence intensity (MFI) ratio from a pool of three donors; y axis, MFI of receptor expression on M2 from a pool of three donors. (B) Peripheral blood CD4 T cells were stained for CD3, HLA-DR, and CD32 and analyzed by AMNIS Imagestream. Shown are bright-field and fluorescent images of cells gated for CD32 positivity. Upper panel, CD32^+^ HLA-DR^+^ CD4 T cells; lower panel, CD32^‒^ HLA-DR^+^ CD4 T cells (see Figure S1D for gating strategy). (C) HLA-DR expression on autologous wild-type (WT) or HLA-DR KO CD4 T cells (KO) and M2 after 2 days of co-culture (mean ± SEM; n = 2–8). Asterisks indicate statistical significance by one-way ANOVA. p values were corrected for multiple comparison (Tukey). (D) DC-SIGN expression on WT CD4 T cells after 2 days co-culture with M2, either non-targeting control (NTC) or DC-SIGN KO (KO) (mean ± SEM; n = 3). Isolated CD4 T cells served as control (-). Asterisks indicate statistical significance by one-way ANOVA. p values were corrected for multiple comparison (Tukey). (E) CD32 expression on PBMCs and CD4 T cells after 3 days of culture in presence of absence of PHA/IL-2 (median with 95% CI; n = 6). Asterisks indicate statistical significance by one-way ANOVA relative to unstimulated (Not stim.) CD4 T cells. p values were corrected for multiple comparison (Tukey). (F) CD32 expression on CD4 T cells, PBMCs depleted of CD14^+^ cells, PBMCs or co-cultures of autologous CD4 T cell/CD14^+^ cells (mean ± SEM; n = 6). Asterisks indicate statistical significance by one-way ANOVA. p values were corrected for multiple comparison (Tukey). (G) CD14^+^ monocytes were differentiated into the indicated myeloid lineages (see Figure S3B) and co-cultured with autologous CD4 T cells for 2 days with or without (Transwell) direct cell-cell contact. Bottom: CD4 T cells migrated to the Transwell bottom and thus had direct contact with differentiated myeloid cells. Mean ± SEM of CD32^+^ T CD4 cells are shown (n = 3). Asterisks indicate statistical significance by two-way ANOVA test. p values were corrected for multiple comparison (Tukey). (H) CD32 expression on CD14^+^ monocytes and cells derived by lineage-specific differentiation after 1 week of cultivation. One representative donor is shown (n = 3). (I) Pearson correlation plot for CD32 surface expression on monocyte-derived cells (MFI) and autologous CD4 T cells (percentage of CD32^+^ cells) after 2 days of co-culture. (J) CD32 expression on CD4 T cells residing in peripheral blood (n = 23), tonsil (n = 6), or lamina propria of jejunum or ileum (n = 6) was assessed by flow cytometry. Median with 95% CI are shown. Asterisks indicate statistical significance by one-way ANOVA. p values were corrected for multiple comparison (Dunnett). ∗p ≤ 0.05, ∗∗p ≤ 0.01, ∗∗∗p ≤ 0.001.

Expression of non-canonical surface markers of immune cells could result from transient *de novo* expression or reflect the occasional transfer of these receptors from other cells. To investigate this phenomenon, co-cultures of M2 macrophages (M2) and autologous resting CD4 T cells were subjected to antibody-based screening by flow cytometry to detect receptors, which are preferentially or exclusively expressed on M2, 2 days later on CD4 T cells. Using settings for the exclusive detection of single cells, 116 out of 242 receptors examined were expressed on M2. A subset of these receptors was detected on co-cultured CD4 T cells, but not or at very low levels when these CD4 T cells were cultured in the absence of M2. These receptors included CD209 (DC-SIGN), HLA-DR, CD97, CD63, CD85, CD195 (CCR5), CD13, CD134, and the Fcγ receptor (FcγR) CD32 (Figures 1A, S1A, and S1B). Imaging flow cytometry analysis, again upon exclusion of cell doublets, revealed that CD32 and HLA-DR co-localized in distinct spots at the surface of single CD32 and HLA-DR double-positive CD4 T cells (Figure 1B, top panels), whereas CD3 and CXCR4 receptors, expressed endogenously by these CD4 T cells, were more evenly distributed (Figure 1B, top panels, and S1C). In contrast, HLA-DR was evenly distributed only on CD32^‒^ CD4 T cells suggesting a small population of cells expressing HLA-DR endogenously (Figure 1B, lower panels, and S1D and S1E). Spotted patterns of CD32 and HLA-DR were observed also on CD4 T cells directly isolated from peripheral blood or from resected tonsil tissue (Figures S1D, S1F, and S1G). Of note, co-culture with M2 for 2 days rendered >15% of CD4 T cells double-positive for CD32 and HLA-DR (Figure S2A). Comparative expression analyses of early T cell activation markers CD69 and CD25, and of HLA-DR on CD4 T cells following M2 co-culture or in response to T cell activation stimuli (Figures S2B and S2C) raised the possibility that CD32 and HLA-DR had both been actively transferred from donor M2 to target CD4 T cells by a cell contact-dependent mechanism, potentially providing an explanation for the recent *in vivo* description of CD32^+^ HLA-DR^+^ CD4 T cells.^19^ Of note, M2 had not been primed to present specific antigens in these co-culture experiments with autologous CD4 T cells. On the surface of CD4 T cells shown in the lower panel of Figure 1B, HLA-DR is evenly distributed and these cells are negative for CD32. Based on this expression pattern, we believe that these may represent CD4 T cells that endogenously express HLA-DR *in vivo*, similar to *in vitro* activated CD4 T cell cultures (Figure S2C), yet neither endogenously express CD32 nor have acquired the Fcγ receptor from other cells.

To validate if these receptors are indeed transferred from macrophages to T cells, the genes of two top transfer candidates, *HLA-DRA* and *DC-SIGN* (Figures 1C and 1D), were knocked out by CRISPR-Cas9 in M2 (knockout [KO] efficiency over 90%, Figure S3A). Co-culture with M2 KOs abolished HLA-DR or DC-SIGN (Figures 1C, 1D, and S3A) surface exposure on co-cultured CD4 T cells, respectively, whereas KO of *HLA-DR* in CD4 T cells^20^ did not impact on their HLA-DR surface exposure following co-culture with HLA-DR^+^ wild-type (WT) M2 (Figure 1H). These results strongly suggested the myeloid cells as the source of these surface-exposed receptors on co-cultured autologous CD4 T cells (Figures 1A and S1A).

Since the FcγR family, which includes CD16A, CD16B, CD32A, CD32B, CD32C, and CD64, has been associated with the transfer and internalization of individual receptors,^21^ we investigated the role of highly transferred CD32 receptors in more detail. To understand the cell-context dependence of CD32 positivity of CD4 T cells, we varied the presence of CD14^+^ monocytes in these cultures. Surface-exposed CD32 was readily detectable only on CD4 T cells in peripheral blood mononuclear cell (PBMC) cultures, but not on previously isolated and cultivated CD4 T cells, irrespective of the cells’ activation status (Figure 1E). Depletion of CD14^+^ cells from PBMC cultures abrogated CD32 positivity of their CD4 T cell population, whereas addition of autologous CD14^+^ cells to previously isolated CD4 T cells drastically increased their CD32 positivity (Figure 1F). Furthermore, terminal differentiation of CD14^+^ monocytes (Figures S3B and S3C) and subsequent co-culture with autologous CD4 T cells revealed that monocyte-derived macrophages, M1 macrophages (M1), or M2 (Figures 1G, direct co-culture and Transwell bottom, and S3D) lead to as much as 66.8% CD32^+^ CD4 T cells. Separation of these two cell populations by a Transwell membrane prevented subsequent CD32 exposure on CD4 T cells (Figure 1G, Transwell top), demonstrating the requirement of a direct macrophage-T cell interaction for CD32 positivity of the latter. In contrast, direct co-culture of monocyte-derived mature and immature dendritic cells (DCs) resulted in only <4.5% of CD32^+^ CD4 T cells (Figures 1G and S3D). Intriguingly, CD32 surface levels on different monocyte-derived cells varied markedly (Figures 1H and S3C) and this correlated positively with the percentage of CD32^+^ CD4 T cells following co-culture (Figure 1I). Among PBMC, also B cells are positive for CD32 thus qualifying as potential FcγR donors. To explore that, we co-cultured isolated CD4 T cells with isolated autologous CD19^+^ B cells and observed increased levels of CD32 exposed on CD4 T cells (Figure S3E), albeit at levels markedly lower compared with M2 co-cultures (4% vs. 66%). Supporting the model of an increased CD32 positivity rate following CD32^+^ cell contacts also *in vivo*, the percentage of CD32^+^ CD4 T cells was higher in cell-rich lymphatic tissue, including tonsil and lamina propria tissue of the intestinal tract, in which contacts between macrophages and T cells are more frequent^22^ compared with peripheral blood (Figure 1J). Collectively, these results indicate that CD4 T cells can efficiently acquire a specific set of surface receptors from autologous macrophages in a cell contact-dependent manner.

### CD32-dependent receptor transfer is enhanced by antibodies and occurs via specialized donor cell membrane nanoprotrusions

**Figure 2:**
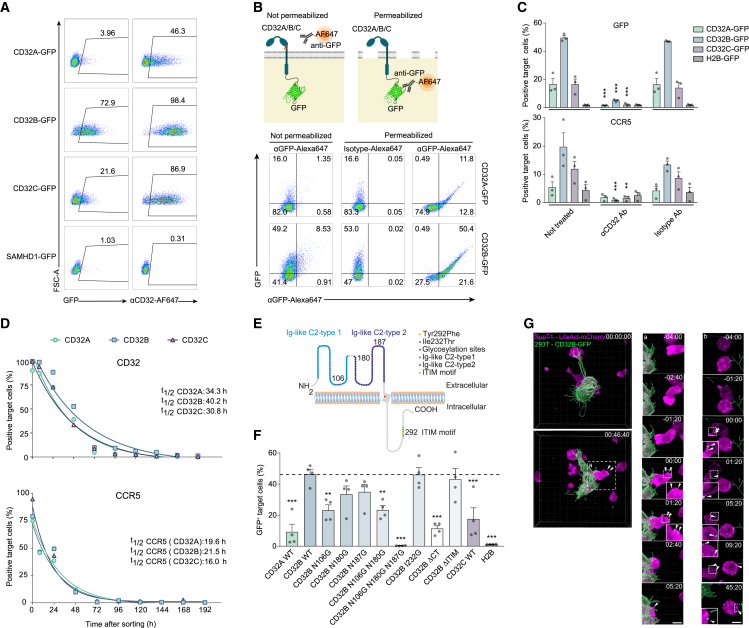
Characterization of CD32-driven trogocytosis (A) 293T cells transiently expressing C-terminal GFP fusion proteins of FcγRs CD32A, CD32B, or CD32C or, as a control, the nucleocytoplasmic dNTPase SAMHD1 served as donors in co-cultures with CellTrace dye-stained SupT1 T target cells. All culture media contained IgG-depleted FCS. Shown are representative flow cytometry dot plots and the percentages of CD32^+^ and GFP^+^ target T cells. One experiment out of two is shown. (B) Schematic of topology determination of transferred CD32-GFP (top). Bottom: SupT1 T cells were co-cultured as described in (A) and stained with either an anti-GFP mAb or an isotype control antibody, both conjugated to Alexa 647, with or without prior cell permeabilization. One representative experiment is shown (n = 3). The illustration was created with BioRender.com. (C) 293T cells were co-transfected with plasmids encoding C-terminal GFP fusion proteins of CD32A, CD32B, or CD32C or, as a control, histone H2B-GFP, together with a plasmid encoding CCR5. After 2 days, cells were either left untreated or pre-treated with an anti-CD32 Ab or an isotype control Ab prior to co-cultivation with SupT1 T cells. One day later, the expression of GFP and CCR5 on the target T cells was determined by flow cytometry. Mean ± SEM are shown (n = 3). Asterisks indicate statistical significance by two-way ANOVA. p values were corrected for multiple comparison (Tukey). (D) Half-life of CD32 and CCR5 surface expression on SupT1 target cells following co-culture as in (A). Following 1 day of co-culture, SupT1 T cells positive for CD32-GFP were sorted by flow cytometry and kept in culture for an additional 9 days. The expression of CD32 (top) or CCR5 (bottom) on sorted cells was determined for up to 192 h of cultivation. One representative experiment is shown (n = 2). (E) Schematic of CD32B with important amino acids and motifs indicated. (F) Transfer of the indicated CD32B mutants, CD32A WT, CD32C WT, or H2B (GFP fusion proteins), assessed as in (A) (mean ± SEM; n = 4). Asterisks indicate statistical significance by one-way ANOVA. p values were corrected for multiple comparison (Dunnett). (G) Visualization of the material transfer from CD32B-GFP expressing 293T cells to LifeAct-mCherry-expressing SupT1 using live-cell imaging. 293T cells transiently expressing CD32B-GFP (green) were co-cultured with LifeAct-mCherry-expressing SupT1 cells (magenta), cultivated in IgG-depleted FCS and boosted with PGT151 antibody, and imaged using spinning disc microscopy for 4 h. The left panel shows the beginning of co-culture. (a) Labels the area with the first transfer event (middle panel). (b) Labels the area of the second transfer event (right panel). Dashed white box marks the area that is zoomed and depicted with individual time points before and after the transfer event (shown below). The time stamp (upper right corner, relative to the time frame which shows the transfer event (time 00:00) in zoom-ins). Scale bar, 10 μm. ∗p ≤ 0.05; ∗∗p ≤ 0.01; ∗∗∗p ≤ 0.001.

To study this process and the mechanistic role of CD32 further, we established a cell line-based donor-target model system. 293T donor cells were transiently transfected with expression plasmids encoding human FcγRs.^23^ Co-cultured SupT1 CD4 T cells, stained with CellTrace dye, served as target cells (Figure S4A). Using this setup, C-terminal GFP fusion proteins of all three CD32 proteins, but not SAMHD1-GFP, which localizes to cytoplasm and nucleus,^24^ were found to be transferred to target CD4 T cells, albeit with variable efficiency (CD32B-GFP > CD32C-GFP > CD32A-GFP; Figure 2A).^25^ Flow cytometry-based detection by anti-CD32 antibody staining was generally more sensitive than detection of the GFP tag (and Figure S4B). The correct membrane topology of transferred FcγRs on CD4 T cells was indicated by the co-detection of CD32 fusion proteins with a C-terminal, intracellular GFP tag by an Alexa 647-conjugated anti-GFP antibody only when cells had been permeabilized (Figure 2B).

We next addressed whether the coinciding transfer of FcγRs and other cell surface receptors (Figure 1A) was linked mechanistically. Remarkably, transfer of the β-chemokine receptor CCR5 (CD195) was triggered upon co-expression with FcγRs in donor cells, with efficiencies descending from CD32B-GFP over CD32C-GFP to CD32A-GFP (Figures 2C, bottom, and S4C, bottom), while histone H2B-GFP co-expression did not induce marked transfer of CCR5. Specific anti-CD32 antibodies blocked the transfer of both the FcγRs and CCR5, indicating that the transfer of CCR5 was dependent on CD32 activity and/or co-transfer (Figures 2C and S4C). Transferred CD32 and CCR5 receptors remained detectable on target SupT1 cells for 3–5 days following separation from 293T donor cells with half-lives (t_1/2_) for surface-exposed CD32 subtypes from 30.8 to 40.2 h (Figure 2D, top) and for CCR5 from 16 to 21.5 h (Figure 2D, bottom), respectively. A similar dynamic was observed for CD32 transferred from macrophages to autologous CD4 T cells (Figure S4D). Together, these results show that CD32, and in particular CD32B, can be inducer and cargo of this intercellular receptor transfer.

Genetic mapping studies (Figure 2E) identified CD32B’s cytoplasmic tail and the N-glycosylation sites in its extracellular domain (Figure 2F), which, in analogy to other FcγRs, are likely required for Fc-mediated antibody binding,^26^ as molecular determinants for efficient receptor transfer, but its immunoreceptor tyrosine-based inhibition motif was dispensible.^27^ Similar observations were made for CD32A and CD32C or CD32 A/B chimeras (Figures S5A–S5D). Moreover, since culturing donor and target cells using bovine serum with reduced IgG levels diminished levels of receptor transfer (Figure S5E), the binding of antibodies to CD32B seemed to be involved in its ability to trigger the receptor transfer.

To gain insight into the dynamics of antibody-enhanced receptor transfer, we attempted to visualize this process between CD32B-GFP^+^ 293T cells and mCherry-expressing SupT1 cells and monitored the transfer events at a high spatiotemporal resolution. To minimize light-induced cytotoxicity, we employed spinning disc microscopy with minimal light coupled with image reconstruction by content-aware image restoration machine learning.^28^ This revealed that 293T donor cells often exhibited long membrane protrusions that made physical contact with SupT1 target cells. These protrusions were thin, did not adhere to the surface of the cell culture dish, and formed and retracted very dynamically despite the lack of detectable actin polymerization (Figures 2G, upper left, and S6A, upper left panel; Videos S1 and S2). Similar protrusions, but at much lower frequency and length, were also observed with 293T cells expressing GPI-anchored GFP or CD32BΔCT (Figure S6A, upper right, lower left), suggesting that induction of CD32B-mediated transfer by antibodies potentiates a cellular activity that has basal activity in non-stimulated cells. In addition, the formation of close cell-cell contacts between donor and target cells bridged via short cell protrusions were often observed (Figure 2G, left panel). In many cases, these short- and long-range contacts of 293T cells resulted in the deposition of CD32B-GFP punctae at the surface of SupT1 cells that resembled those previously observed by image stream analysis (Figures 1B and S6B; Videos S1 and S2).

Video S1. Time-lapse 3D reconstruction of a live-cell imaging showing the transfer of CD32B-GFP, related to Figure 2

Video S2. Detailed view of the first transfer event from video S1, related to Figure 2

Together, the key characteristics of this receptor transfer include a spotted distribution on single target cells (Figure 1B) and a strict dependence on direct cell-cell contact (Figure 1G), which excludes vesicle transfer as a major contributor. Instead, transfer occurs via long-range plasma membrane nanoprotrusions from donor to target cells, that are, however, F-actin negative (Figure 2G), and is independent from antigen presentation or recognition. We conclude that this intercellular exchange of plasma membrane components most likely reflects a form of antibody-enhanced, FcγR-driven trogocytosis.^29^^,^^30^^,^^31^^,^^32^^,^^33^^,^^34^^,^^35^^,^^36^

### T cell-reactive autoantibodies found in individuals living with HIV-1 enhance trogocytosis

**Figure 3:**
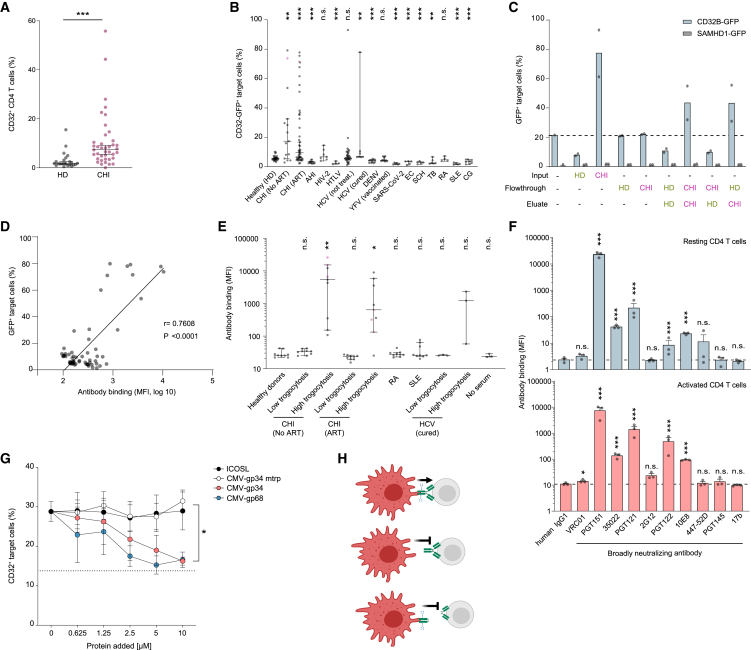
CD32-driven trogocytosis is boosted by T cell-autoreactive antibodies associated with chronic HIV-1 infection (A) CD32 expression on CD4 T cells from peripheral blood of healthy donors (HD) (n = 23) and chronic HIV-1 infected patients (CHI) (n = 39). Median with 95% CI are shown. Asterisks indicate statistical significance by Mann-Whitney test. (B) 293T cells transiently co-expressing CD32B-GFP and CCR5 were pre-treated with the indicated patient sera before 1 day of co-culture with SupT1 T cells. Shown are the percentage of CD32B-GFP^+^ and CCR5^+^ target cells (median with 95% CI, each dot represents a different patient; see also Figure S7C). CHI, chronic HIV-1 infection; ART, anti-retroviral therapy; AHI, acute HIV-1 infection. Fiebig stages II-III of acute HIV-1 infection^42^; HIV-2, HIV type 2; HTLV-1, human T cell lymphotropic virus type 1; HCV, hepatitis C virus; DENV, dengue virus; YFV, yellow fever virus-vaccinated; SARS-CoV-2, severe acute respiratory syndrome coronavirus type 2; EC, *Echinococcus multilocularis*; SCH, *Schistosoma* spp.; TB, *Mycobacterium tuberculosis*; RA, rheumatoid arthritis; SLE, systemic lupus erythematosus; CG, cryoglobulinemia. Asterisks indicate statistical significance by Mann-Whitney test. (C) Percentage of GFP^+^ target cells after 1 day of co-culture with 293T cells as in (B). IgG was depleted from the sera of two healthy donor (HD) and two HIV-1 patient (CHI) samples from (B, pink and red) and input (original sera), flowthrough and eluate of the IgG depletion were used for pre-treatment of cells prior to co-culture. Mean of two donors from each category is shown. (D) Correlation of antibody binding to SupT1 T cells and CD32B-GFP trogocytosis as in (B), with sera from HIV-1 patients. P, Pearson correlation coefficient. (E) Binding of sera with high or low trogocytotic activity (pink and red dots in B) to primary CD4 T cells as detected with fluorochrome-coupled anti-human IgG Ab (median with 95% CI, CD4 T cells; n = 3). Kruskal-Wallis test with Dunn’s multiple-testing correction. (F) A panel of bNAbs was analyzed for binding to uninfected resting CD4 T cells (top) or activated CD4 T cells (bottom). Mean ± SEM; n = 3. Asterisks indicate statistical significance by one-way ANOVA (top) or three-way ANOVA (bottom). p values were corrected for multiple comparison (Dunnett). (G) Purified, CMV-encoded, soluble Fc-binding proteins gp34 and gp68, or control proteins gp34 non-binding mutant (mtrp; W65F) and soluble ICOSL (inducible T cell co-stimulator ligand) were added to 293T donor cells as in (A), in the presence of PGT151 Ab, and subsequently co-cultured with SupT1 T cells. CD32 transfer was evaluated as in (B). Asterisks indicate statistical significance by two-way ANOVA. p values were corrected for multiple comparison (Tukey). ∗p ≤ 0.05, ∗∗p ≤ 0.01, ∗∗∗p ≤ 0.001; n.s., not significant. (H) Schematic of the determinants of antibodies for trogocytosis enhancement.

Considering potential pathophysiological consequences of antibody-enhanced trogocytosis, we addressed whether, as recently reported by Badia et al.,^37^ CD32 expression on unstimulated CD4 T cells is elevated in blood from HIV-infected compared with uninfected individuals. Indeed, the percentage of CD32^+^ CD4 T cells was significantly increased in PBMCs from patients with chronic HIV-1 infection (CHI) compared with healthy donors (HD) (Figures 3A, S7A, and S7B). Since antibodies can modulate trogocytosis,^21^^,^^38^^,^^39^^,^^40^ we hypothesized that (auto)antibodies specific to viral, bacterial, or parasitic infections or to self-antigens (in autoimmune diseases) may contribute to certain disease-specific pathologies by activating or interfering with this type of cell communication. We therefore tested the trogocytosis-triggering ability of serum samples from patients suffering from different infectious diseases or autoimmune diseases. Remarkably, sera from 20.5% of individuals with CHI (n = 122) enhanced the transfer of CD32B-GFP (Figure 3B; ART, 17.8%; no ART, 40%) and CCR5 (Figure S7C). This boosting effect did not correlate with the levels of IgGs in patient sera (Figures S7D–S7F) and was not observed in sera from HD or from patients with acute HIV-1 infection (n = 12), chronic infections with the closely related HIV-2 (n = 7), or the other major pathogenic lentivirus, human T-lymphotropic leukemia virus (HTLV) (n = 4) (Figure 3B). Apart from two hepatitis C virus cases (n = 40), most of the patient’s sera from other viral infections (SARS-CoV-2 [n = 6], dengue virus [n = 11], following attenuated yellow fever virus vaccination [n = 10]), parasitic infections (*Echinococcus multilocularis* [EC] [n = 5], *Schistosoma* spp. [SCH] [n = 5]) or a chronic bacterial infection (*Mycobacterium tuberculosis* [TB] [n = 6]) negatively affected trogocytosis (Figure 3B). Notably, also sera from patients suffering from autoimmune diseases, i.e., rheumatoid arthritis (n = 4), systemic lupus erythematosus (SLE) (n = 5), or cryoglobulinemia (CG) (n = 8) either did not or even negatively impact on CD32B or CCR5 trogocytosis (Figures 3B and S7C). This suggests that soluble immune complexes alone rather inhibit CD32B-mediated trogocytosis,^41^ while its induction is mediated by disease-related antibodies with specific features.

To further examine these trogocytosis-triggering properties, we assessed the role of IgG in the serum of CHI donors compared with HD. Column-based fractionation demonstrated that IgG depletion from HIV sera reduced trogocytosis of CD32B-GFP to background levels, while addition of the eluted IgG fraction from HIV-1 patients’ sera to the column flowthrough fraction (from either IgG-depleted CHI donors or HD) boosted trogocytosis (Figures 3C and S8). The requirement of both cell-cell contact (Figure 1G) and specific IgGs (Figure 3C) suggested a model, in which this type of trogocytosis is facilitated by the binding of a specific antibody’s Fab part to the surface of target T cells and their Fc part to CD32 expressed on donor cells. Indeed, the ability of patient sera to induce trogocytosis positively correlated with their IgGs’ ability to bind to SupT1 T cells (Figure 3D). This T cell autoreactivity of trogocytosis-boosting sera was seen also for primary CD4 T cells (Figure 3E).

Interestingly, among a panel of 10 broadly neutralizing monoclonal anti-HIV antibodies (bNAbs) targeting the HIV-1 envelope, four bNAbs, i.e., PGT151,^43^ 35022,^44^ PGT121,^45^ and PGT122^45^ were found to bind to the surface of uninfected primary CD4 T cells, in part dependent on the activation status of these cells (Figure 3F, compare top and bottom panels). Interestingly, binding of PGT151 to resting or activated CD4 T cells was significantly reduced by the α-mannosidase II inhibitor swainsonine or the α-mannosidase I inhibitor kifunensine, indicating that the antibody recognizes an N-glycosylated antigen (Figures S9A and S9B). Notably, the cell surface reactivity of bNAbs and of sera from HIV-1 patients was independent of cells expressing CD4. In fact, this reactivity was found in most cases also against parental 293T cells. PGT151 also bound to T cells in tonsillar tissue (HLAC) and lamina propria (LPAC) (Figure S9C) and induced trogocytosis in an FcγR domain-dependent pattern (Figure S9D, see also Figures 2E and 2F). Similar to bNAb PGT151, alemtuzumab (Lemtrada), a therapeutic humanized monoclonal antibody, which recognizes CD52 on mature T cells,^46^ bound to CD4 T cells (Figures S9C and S9E) and, importantly, triggered trogocytosis of both CD32B and CCR5 (Figure S9F).

IgGs contain a conserved N-glycosylation site at N297 in the Fc region that affects their interaction with FcγRs,^47^ and trogocytosis enhancement by PGT151 required the N-glycosylation sites in CD32 (Figures S10A–S10F): Endoglycosidase treatment to remove N-linked glycans from PGT151 disrupted both the binding to CD32 and trogocytosis enhancement, while the antibody’s ability to neutralize HIV-1 was preserved. Moreover, disrupting the overall domain organization of PGT151 or alemtuzumab by papain digestion showed that their Fab or Fc antibody parts alone were insufficient to boost trogocytosis (Figures S11A–S11E and 3H). Finally, the cytomegalovirus (CMV) glycoproteins 34 and 68 (gp34 and gp68), which specifically recognize the Fc part of human IgG and impair FcγR activation,^48^ reduced PGT151-dependent trogocytosis in a dose-dependent manner. In turn, neither the CMV-gp34 mtrp point mutant that lacks IgG binding^49^ nor the inducible T cell co-stimulator ligand impaired PGT151-dependent trogocytosis (Figures 3G and S12A). Furthermore, co-transfer of CD32 to target T cells in the presence of PGT151 was observed for several exogenously co-expressed cell surface transmembrane receptors (CXCR4, CXCR7, CD4) and, albeit with lower efficiency, for proteins that are peripherally associated with the inner leaflet of the plasma membrane, i.e., membrane-targeting domains of Lck and Fyn (LckN18, FynN18), but not for nucleocytoplasmic SAMHD1 (Figures S12B and S12C). Altogether, these results establish that the observed transfer of cell surface receptors from donor to target cells results from CD32-dependent trogocytosis of membrane patches with multiple cargo molecules, which is facilitated by IgG antibodies reactive to the surface of T cells.

### Receptor trogocytosis confers functional plasticity to immune cells

**Figure 4:**
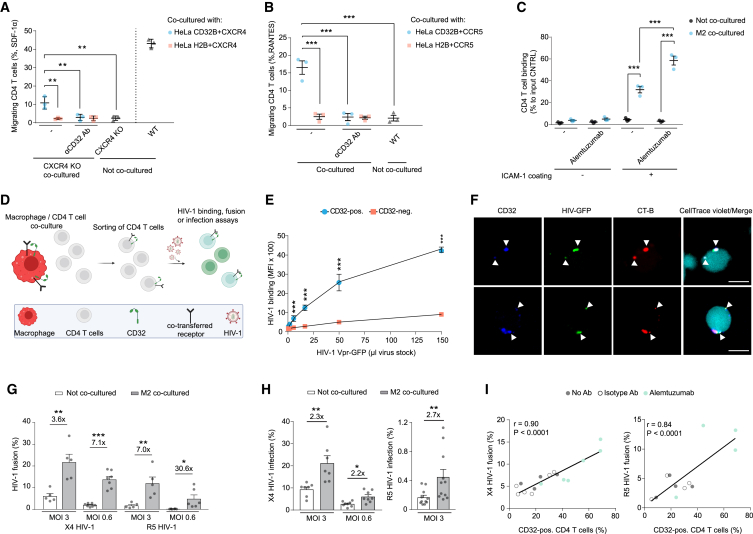
Trogocytosed receptors are functional and CD32^+^ membrane patches on resting CD4 T cells preferentially bind virions and enhance HIV-1 fusion (A) SDF-1α (CXCL12)/CXCR4 migration assay. CXCR4 KO CD4 T cells were co-cultured with HeLa cells transiently co-expressing CD32B-GFP or H2B-GFP (control) together with CXCR4. Prior to co-culture, HeLa donor cells were pre-treated with or without anti-CD32 mAbs. One day after co-culture, CD4 T cells were placed into the top chamber of a Transwell and SDF-1α was added to the bottom chamber. Migrating cells collected were counted by flow cytometry. CXCR4 WT and KO CD4 T cells without co-culture were used as positive and negative control (mean ± SEM; n = 3). Asterisks indicate statistical significance by one-way ANOVA. p values were corrected for multiple comparison (Tukey). (B) RANTES (CCL-5)/CCR5 migration assay. HeLa cells transiently co-expressing CD32B-GFP and CCR5 were co-cultured with CD4 T cells and the latter analyzed for migration toward CCL-5 (assay setup as in A) (mean ± SEM; n = 3). Asterisks indicate statistical significance by one-way ANOVA. p values were corrected for multiple comparison (Dunnett). (C) CD11b binding assay. Following co-culture of M2 with autologous CD4 T cells for 48 h, T cells were sorted and cultured in plates coated with or without the ICAM-1 ligand. Attached cells were quantified by luminometry (mean cell binding ± SEM normalized to wells with input cells without washing; n = 3). Asterisks indicate statistical significance by two-way ANOVA. p values were corrected for multiple comparison (Tukey). (D) HIV-1 binding, fusion, or infection of (CellTrace+) CD4 T cells following co-culture with autologous M2. The illustration was created with BioRender.com. (E) HIV-1 binding assay. M2-co-cultured CD4 T cells were sorted and challenged with HIV-1 Vpr-GFP particles. Shown is GFP and CD32 positivity of target CD4 T cells (mean ± SEM; n = 4). Asterisks indicate statistical significance by two-way ANOVA (see Figures S14A–S14C for confocal microscopy images). p values were corrected for multiple comparison (Tukey). (F) CD4 T cells were co-cultured with biotin-xx-conjugated cholera toxin subunit-B (CT-B)-labeled M2, sorted, challenged with HIV-1 Vpr-GFP (see also Figures S14D–S14F), and stained for CD32 and fluorochrome-conjugated streptavidin. Shown are representative confocal microscope micrographs. White arrow heads: co-localization of CD32, HIV-1 Vpr-GFP, and CT-B. Scale bars, 5 μm. (G) HIV-1 fusion assay. CD4 T cells were co-cultured with autologous M2, isolated and used in an HIV-1 fusion assay using two multiplicities of infection (MOIs). Shown is the percentage of cells that allowed virion fusion (mean ± SEM; n = 5). Asterisks indicate significance by two-tailed paired t test. ∗p ≤ 0.05, ∗∗p ≤ 0.01, ∗∗∗p ≤ 0.001; n.s., not significant. (H) HIV-1 infection assay. CD4 T cells were co-cultured with autologous M2, isolated, and infected with HIV-1 at different MOIs. Shown is the percentage of infected cells (mean ± SEM; n = 7–11). Asterisks indicate significance by two-tailed paired t test. (I) M2 were pre-treated with alemtuzumab or an isotype control antibody and then co-cultured with autologous CD4 T cells. Sorted CD4 T cells were incubated with X4 HIV-1 (left panel) or R5 HIV-1 (right panel), carrying Vpr-BlaM, and virion fusion was quantified. Pearson correlations between CD32 positivity and HIV-1 fusion are shown. ∗p ≤ 0.05, ∗∗p ≤ 0.01, ∗∗∗p ≤ 0.001; n.s., not significant.

We next addressed the functionality of trogocytosed chemokine receptors CXCR4 (CD184) and CCR5 (CD195) on target T cells. Chemotaxis of primary CD4 T cells toward the natural CXCR4 ligand SDF-1α (CXCL12) was abolished following genetic ablation of CXCR4, yet partially restored following co-culture of CXCR4 KO CD4 T cells with CD32B-GFP-expressing, CXCR4^+^ donor cells (Figures 4A, S13A, and S13B). Similarly, robust RANTES (CCL5)-mediated chemotaxis of freshly isolated primary CD4 T cells, with low or no endogenous expression of CCR5, was only observed following co-culture with CD32B-GFP/CCR5-co-expressing donor cells (Figures 4B and S13C). In line with an essential role of trogocytotic transfer of CXCR4 and CCR5, acquisition of the migratory function was not observed for H2B-GFP/chemokine receptor-co-expressing donor cells and could be blocked by addition of anti-CD32 antibodies (Figures 4A, 4B, and S13A–S13C). Moreover, transfer of the CD11b receptor between autologous primary cells, i.e., from M2 macrophages to CD4 T cells (Figures S13D and S1E), increased the ability of the latter to bind surfaces coated with the CD11b ligand ICAM1, and this adhesion function was boosted by addition of alemtuzumab (Figure 4C). Together, these studies further underscore the correct topology of transferred receptors (Figure 2B) and demonstrate their functionality for chemotaxis and ligand binding in primary cells.

Resting CD4 T cells are largely refractory to HIV-1 infection, yet these cells constitute an important reservoir of virus persistence. Following up on the controversy over the role of CD32 in HIV-1 biology,^19^^,^^37^^,^^50^^,^^51^ we sought to explore the relationship between HIV-1 infection and CD32 expression on CD4 T cells. To this end, we quantified the CD32 surface levels of PBMCs 3 days after challenge with HIV-1 GFP by flow cytometry. As indicated by expression of the GFP reporter, 9.3% of all CD4 T cells were productively infected (Figure S13F, left). Among these, 28% (2.64% of all cells) were positive for CD32, but significant levels of CD32^+^ cells (4.59% of all cells, corresponding to 63% of all CD32^+^ cells) were also observed in the uninfected, GFP-negative cell fraction. In contrast, CD32 was nearly undetectable on CD4 T cells (0.18% CD32^+^ cells) that had been infected following isolation from PBMCs by negative selection, despite comparable overall HIV-1 GFP infection levels (Figure S13F, right). Next, we explored whether trogocytosis can affect the susceptibility of primary CD4 T cells to HIV-1. To this end, these cells were first co-cultured with autologous M2 and then separated by cell sorting to allow functional analyses (Figure 4D). Binding of HIV-1 particles using CXCR4 as entry co-receptor (X4 HIV-1), carrying Vpr-GFP, to sorted CD4 T cells was strongly enhanced for the fraction of CD32^+^ cells compared with CD32^−^ cells in the same culture (Figure 4E) and confocal microscopy images revealed that X4 HIV-1 Vpr-GFP particles preferentially bound to CD32^+^ membrane patches (Figures 4F, S14A, and S14C). Moreover, pre-labeling ganglioside GM1, a typical constituent of lipid rafts, on M2 with cholera toxin subunit-B prior to co-culture showed that trogocytosed membrane patches with high HIV-1 binding capacity were GM1^+^ CD32^+^ (Figures 4F,S14D, and S1F). Importantly, these CD4 T cells also displayed an increased capacity to support fusion of HIV-1 particles, on average 7.1-fold for X4 HIV-1 and 30.6-fold for CCR5-using (R5) HIV-1, respectively (Figures 4G and S15A). Moreover, also productive HIV-1 infection as assessed by viral reporter gene expression was significantly enhanced in M2-co-cultured CD4 T cells (Figure 4H), and HIV-1 fusion efficacy correlated with the extent of receptor CD32 trogocytosis (Figure 4I). Of note, overexpression of CD32 alone in primary CD4 T cells following nucleofection of an expression plasmid was not sufficient to increase HIV-1 fusion (Figures S15A–S15C) indicating the importance of trogocytosis of CD32^+^ M2 membrane patches for this functionality.

To characterize why HIV-1 preferentially binds to trogocytosed CD32^+^ plasma membrane patches, we performed a small-scale CRISPR-Cas9 KO screen targeting M2 receptors previously implicated in HIV-1 binding,^52^^,^^53^^,^^54^^,^^55^^,^^56^^,^^57^^,^^58^ including CD206, CD209 (DC-SIGN), CD11a, CD11b, and CD11c, followed by co-culture with CD4 T cells. Disruption of expression of none of these receptors impacted trogocytosis and, more importantly, HIV-1 binding (Figures S16A–S16C) or HIV-1 fusion (Figure S16D). These data were confirmed using combinations of antibodies against the same receptors (Figure S16E). Next, we found that heparinase/chondroitinase treatment reduced HIV-1 GFP binding to HeLa cells (Figure S17A), while not affecting virus binding to M2-co-cultured CD4 T cells (Figure S17B).

**Figure 5:**
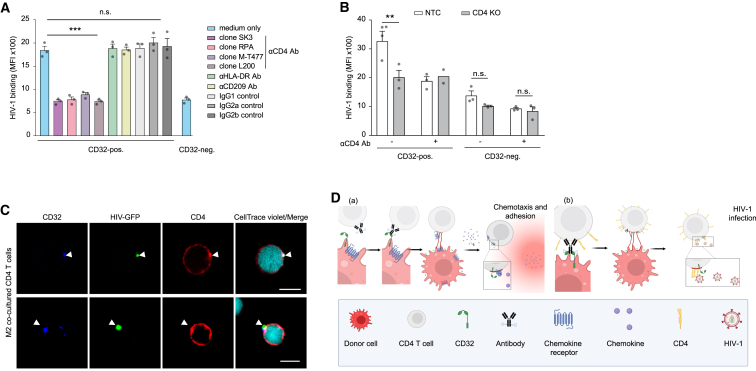
Transferred membrane patches on resting CD4 T cells preferentially enhance HIV-1 fusion and infection by endogenously expressed CD4 recruitment (A) HIV-1 binding to CD4 T cells following co-culture with M2 and addition of anti-CD4 antibodies, isotype control antibodies, or antibodies against efficiently transferred receptors (mean ± SEM; n = 3). Asterisks indicate statistical significance by two-way ANOVA. p values were corrected for multiple comparison (Šídák). (B) HIV-1 binding to CD4 KO or NTC CD4 T cells after co-culture with autologous M2 (mean ± SEM; n = 3). Asterisks indicate statistical significance by two-way ANOVA. p values were corrected for multiple comparison (Šídák). (C) Confocal microscope images of CD32 and CD4 localization on CD4 T cells that were challenged with HIV-1 Vpr-GFP following co-culture with unlabeled M2. White arrow heads indicate the co-localization of CD32, HIV-1 Vpr-GFP, and clustered CD4 (see also Figure S16C). Scale bar, 5 μm. ∗p ≤ 0.05, ∗∗p ≤ 0.01, ∗∗∗p ≤ 0.001; n.s., not significant. (D) Schematic model of trogocytotic transfer of CD32^+^ membrane patches from macrophages to CD4 T cells resulting (a) in the transfer of functional chemokine receptors to CD4 T cells with macrophage-like chemotactic properties and adhesion behavior and/or (b) the recruitment of the endogenous CD4 receptor to these specialized membrane sites providing functional platforms for enhanced binding and infection of HIV-1.

Unexpectedly, incubation of co-cultured CD4 T cells with anti-CD4 antibodies as well as inoculation with HIV-1 ΔEnv particles reduced HIV-1 binding to CD32^+^ cells to levels found for CD32^−^ cells (Figures 5A, S17C, and S17D). Importantly, genetic perturbation of the *CD4* gene in T cells, but not in M2 donor macrophages, mirrored this phenotype (Figures 5B and S17E–S17I). Corroborating a crucial role of CD4 in trogocytosis-mediated enhanced HIV-1 infection, we observed an accumulation of CD4 in CD32^+^ plasma membrane patches that co-localized with HIV-1 GFP particles (Figure 5C). This indicates that, after trogocytosis of M2-derived membranes, the endogenous CD4 receptor on T cells is preferentially recruited, possibly by lateral movement, into these patches creating a hotspot for HIV-1 binding and entry (Figure 5D). Trogocytosed receptors can thus exert complex biological activities on target cells and this process is hijacked by HIV-1 to increase the permissivity of resting CD4 T cells to infection.

## Discussion

Analyzing co-cultures of macrophages with CD4 T cells revealed that the transfer of membrane patches and associated proteins is far more frequent than appreciated previously. The process we identify here relies on direct cell-cell contact and shares features of previously described forms of intercellular trogocytotic membrane transfer in that it leads to the deposition of membrane patches that are inserted into target cells at the original membrane topology.^59^^,^^60^^,^^61^ In our case, the FcγR CD32, rather than antigen-dependent immune cell communication, drives the transfer of plasma membrane patches, which can be boosted by specific antibodies. Moreover, imaging at high spatiotemporal resolution unveiled long (up to 100 μm), highly dynamic filamentous nanoprotrusions that extend from CD32^+^ donor cell surfaces and deposit lipid raft-like membrane patches in an actin-independent manner onto the surface of T cells. This type of trogocytotic transfer thus reflects a “forced deposition” of membrane patches onto T lymphocytes rather than the typical trogocytic “extraction” by the target T lymphocyte. In cell lines as well as primary cells, transferred receptors have the correct orientation in the recipient cells and are functional. We therefore propose to consider this mode of cell-cell communication as long-distance, antibody-driven trogocytosis with features distinct from classical trogocytotic mechanisms.

Our analyses provided important mechanistic insight into this process. All three CD32 proteins are able to support this trogocytotic membrane transfer with CD32B displaying the highest activity. Both the FcγR’s cytoplasmic tail and N-glycosylation sites in the extracellular domain of CD32 are required to exert this activity. While this mapping is consistent with the ability of antibodies to regulate this process, the presence of CD32^+^ membrane patches on freshly isolated CD4 T cells from peripheral blood and tonsils of healthy donors suggests that trogocytotic receptor transfer occurs at a basal level under physiological conditions, and also if cells are co-cultured in bovine serum-containing medium. However, a disease- and autoantibody-dependent modulation of trogocytosis is observed in infection and autoimmunity: chronic HIV-1 infection elicits anti-Env antibodies that autoreact to epitopes on the surface of CD4 T cells that can boost this receptor transfer, and the analysis of HIV patient sera and a number of bNAbs suggests that the level of surface autoreactivity correlates with the ability to foster trogocytosis. In our experiments, bNAbs not only bound to CD4 T cells but also to CD32^+^ donor cells. The N-glycosylated antigen recognized by the trogocytosis-enhancing bNAb PGT151 or others is not known. In contrast, other infections (HTLV, SARS-CoV-2, TB, EC, SCH) or autoimmune diseases (CG, SLE) suppress basal trogocytosis activity. The mechanism of the latter is entirely unclear; we speculate that disease-specific immune complexes may induce signaling in either donor or target cells that curtails trogocytosis. The trogocytosis-enhancing activity did not correlate with the concentration of IgG in patient sera but was correlated to their ability to bind to the surface of T cells. Besides certain bNAbs and HIV-1 sera, this was also true for an anti-CD4 antibody and the anti-CD52 antibody alemtuzumab. This suggests that potentially any antibody that binds to the surface of T cells can induce trogocytosis from CD32^+^ cells to CD4 T cells. Together, qualitative rather than quantitative features of antibodies in patient sera govern the ability to promote trogocytosis.

Basal CD32-driven trogocytosis in different lymphoid compartments of healthy individuals as well as its boosting by pathological autoantibodies increase the plasticity of the surface proteome of target immune cells. These functional alterations are transient and their duration defined by the half-life of the deposited molecules, which may be a particularly efficient way of immune cell communication without the need for prior gene expression and protein synthesis. Functional alterations of target cells include macrophage-like migration and adhesion properties and rendering resting CD4 T cells susceptible to HIV-1 infection. These CD32-dependent processes may contribute to the expansion of the resting CD4 T cell reservoir in HIV patients. Some studies have suggested an effect of Fc receptor genetic diversity on HIV-1 transmission,^62^ pathogenesis,^63^ and reservoir size,^64^ while others could not confirm these findings,^65^^,^^66^ leading to an ongoing debate.^67^^,^^68^ The transient nature of CD32 positivity on CD4 T cells may in part reconcile the controversial findings regarding the role of CD32 as a biomarker for the latent HIV reservoir.^37^^,^^51^^,^^69^^,^^70^^,^^71^ How T cell-encoded CD4 accumulates at these privileged CD32^+^ GM1^+^ membrane sites at the cell surface and how this creates a transiently more favorable microenvironment for HIV-1 binding and fusion in these otherwise hard-to-infect primary reservoir cells remains unclear. We speculate that specific membrane components, e.g., lipid raft-like microdomains^72^ or integrins,^73^^,^^74^ transferred from macrophages to CD4 T cells, may in conjunction with endogenous CD4 contribute to the increased “stickiness” of CD32^+^ membrane patches for HIV-1 particles and increase the local virus concentration to boost infection. Importantly, this membrane transfer is enhanced by T cell-reactive autoantibodies, but not immune complexes, found in a subset of HIV-1 patients and in particular by some of the rare antibodies that broadly neutralize HIV. This implies that HIV hijacks this trogocytic transfer and exploits neutralizing antibody responses to boost its spread and expand the latent reservoir. In addition to representing an immune evasion strategy of HIV-1, the consequences of such trogocytosis need to be considered when assessing the efficacy and safety profiles of bNAbs for clinical application.^75^^,^^76^^,^^77^^,^^78^ In contrast to CAR-based immunotherapies, in which adverse effects due to trogocytosis are triggered by Fc-mediated antibody binding to the acceptor cells, the process we describe elicits antibody-mediated trogocytosis upon binding to the FcγR on the donor cell. Immunotherapies will thus require optimization to circumvent both types of cell-cell communication.

FcγR-mediated trogocytosis expands the functional repertoire of immune cells and HIV-1 exploits this process to persist. This insight may inform HIV patient stratification for current cure approaches and interference with this intercellular communication mode may open avenues to eradicate HIV-1. In general, non-canonical receptor transfer and exposure expand the spectrum of intercellular communication as well as gene regulation to broaden functionality. Therapeutic monoclonal antibodies as well as disease-specific autoantibodies emerge as key regulators of this process.

### Limitations of the study

Our findings raise a number of important questions that we were not yet able to address: While our results provide direct evidence that trogocytosed surface receptors alter the cell migration and adhesion behavior as well as susceptibility to HIV-1 infection of primary target cells, the mechanistic studies on the molecular determinants for transfer as well as the precise membrane topology of transferred receptors studied herein in model cell lines remain to be corroborated in primary cells. Future work is also required to define the identity of the N-glycosylated proteins that are recognized by transfer-triggering autoantibodies on recipient cells. Equally intriguing yet unresolved is the molecular basis that defines whether or not HIV-1 patients develop such trogocytosis-competent autoantibodies, which warrants a broad characterization of clinical and genetic parameters of the respective patients, but also the predominant virus variant they harbor. Finally, it remains unclear how the transfer of the entry receptor-containing membrane patches to CD4 target cells facilitates post-entry steps of the HIV-1 life cycle. This may, e.g., involve the local reorganization of protein and or lipid content including alterations in signal transduction and cell activation states, and distinguishing between these possibilities will require the application of advanced subcellular omics technology.

## STAR★Methods

### Key resources table

**Table:** 

REAGENT or RESOURCE	SOURCE	IDENTIFIER
**Antibodies**		

Alemtuzumab®	LMU hospital Munich	N/A
Anti-human CXCR4 BV421	BD	Cat#562448; RRID:AB_11153865
Anti-human CXCR4 APC	BD	Cat#555976; RRID:AB_398616
Anti-human CXCR4 PE-Cy5	BD	Cat#555975; RRID:AB_396268
Anti-human CXCR4 PE-Cy7	Biolegend	Cat#306514; RRID:AB_2089652
Anti-human CXCR2 APC	BD	Cat#551127; RRID:AB_398492
Anti-human CXCR7 APC	Biolegend	Cat#391405; RRID:AB_2565682
Anti-human CD25 BV421	BD	Cat#562442; RRID:AB_11154578
Anti-human CD25 APC	BD	Cat#555434; RRID:AB_398598
Anti-human CD69 BV421	BD	Cat#562884; RRID:AB_2687422
Anti-human CD69 APC	BD	Cat#555533; RRID:AB_398602
Anti-human CD32 PerCP-Cy5.5	Biolegend	Cat#303216; RRID:AB_2616925
Anti-human CD32 PE-Cy7	Biolegend	Cat#303214; RRID:AB_2616922
Anti-human CD32 AF647	Biolegend	Cat#303212; RRID:AB_2262705
Anti-human CD32 PE	Sony Biotechnology	Cat#2116030
Anti-human CD32 Bv421	BD	Cat#564838; RRID:AB_2738976
Anti-human CD32, clone FUN-2	Biolegend	Cat#303202, RRID:AB_314333
Anti-human CD32, clone FL8.26	BD	Cat#557333; RRID:AB_396647
Anti-human CD32, clone IV.3	Stemcell Technologies	Cat#60012; RRID:AB_2925215
Anti-GFP AF647	Biolegend	Cat#338005; RRID:AB_1279411
Anti-human CCR5 APC	BD	Cat#556903; RRID:AB_398619
Anti-human IgG Fc AF647	Biolegend	Cat#409320, RRID:AB_2563329
Anti-human CD11b APC	Thermo Fisher Scientific	Cat#12-0118-42, RRID:AB_2043799
Anti-human CD11b FITC	BioLegend	Cat#301330; RRID:AB_2561702
Anti-human CD11b Bv421	BioLegend	Cat#301324; RRID:AB_10933087
Anti-human CD11a APC	BioLegend	Cat#301212; RRID:AB_314150
Anti-human CD11c FITC	BioLegend	Cat#337213; RRID:AB_1877174
Anti-human HLA-DR FITC	BD	Cat#347363; RRID:AB_400291
Anti-human CD209 BV421	Biolegend	Cat#330118; RRID:AB_2734323
Anti-human CD206 APC	BD	Cat#550889; RRID:AB_398476
Anti-human CD14 FITC	Biolegend	Cat#325603; RRID:AB_830676
Anti-human CD19 FITC	Biolegend	Cat#302206; RRID:AB_314236
Anti-human CD3 APC -Cy7	BD	Cat#557832; RRID:AB_396890
Anti-human CD4 PE-Cy7	Biolegend	Cat#300512; RRID:AB_314080
Anti-human CD4 APC	BD	Cat#555349; RRID:AB_398593
Anti-human CD4 AF594	Biolegend	Cat#300544; RRID:AB_2563235
Anti-human CD4, clone SK3	Biolegend	Cat#344602; RRID:AB_1937277
Anti-human CD4, clone RPA-T4	BD	Cat#555344, RRID:AB_395749
Anti-human CD4, clone L200	BD	Cat#556614; RRID:AB_396486
Anti-human CD4, clone M-T477	BD	Cat#550625; RRID:AB_393787
Anti-human CD11b, clone VIM12	Santa Cruz Biotechnology	Cat# sc-59744; RRID:AB_781899
Anti-human CD11b, clone ICRF44	Biolegend	Cat#301361; RRID:AB_2814118
Anti-human CD11c, clone Bu15	Biolegend	Cat#337202; RRID:AB_1236381
Anti-human CD206, clone 19.2	BD	Cat#555953; RRID:AB_396249
Anti-human CD206 clone 15-2	Biolegend	Cat#321149, RRID:AB_2819952
Anti-human CD209, clone C209	NeoBiotechnologies	Cat#30835-MSM1-P1ABX
Anti-human CD13, clone DC28	R&D System	Cat#MAB16211; RRID:AB_2074320
Anti-human CD209, clone WM15	Biolegend	Cat#301723; RRID:AB_2728236
Anti-human CD226, clone TX25	Biolegend	Cat#337102, RRID:AB_1236383
Anti-human CD74, clone LN2	Biolegend	Cat#326802; RRID:AB_893401
Anti-human CD54, clone HCD54	Biolegend	Cat#322721; RRID:AB_2832633
Anti-human CD227, clone 16A	Biolegend	Cat#355602; RRID:AB_2561641
Anti-human HLA-DR, clone L243	Biolegend	Cat#307665; RRID:AB_2800798
Anti-human CCR5, clone 2D7	BD	Cat# 555990; RRID:AB_396276
Purified Mouse IgG1, κ Isotype Ctrl Antibody	Biolegend	Cat#400102; RRID:AB_2891079
Purified Mouse IgG2a, κ Isotype Ctrl Antibody	Biolegend	Cat#401501, RRID:AB_2800437
Purified Mouse IgG2b, κ Isotype Ctrl Antibody	Biolegend	Cat#400302
Ultra-LEAF™ Purified Human IgG1	BioLegend	Cat# 403501; RRID:AB_2927629
Anti-human IgG Fcγ F(ab’)2 Goat PE	Biolegend	Cat#398004; RRID:AB_2820063
Anti-human IgG Fcγ F(ab’)2 Goat APC	Jackson ImmunoResearch	Cat#109-136-170; RRID:AB_2337695
Anti-human IgG Fcγ F(ab’)2 Goat HRP-conjugated	Jackson ImmunoResearch	Cat#109-035-003; RRID:AB_2337577
Anti-human IgG F(ab’)2 Goat HRP-conjugated	Abcam	Cat#ab87422; RRID:AB_1951105
Anti-human IgG (Fc-specific) HRP-conjugated	Sigma-Aldrich	Cat#A0170; RRID:AB_257868
FcBlock	BD	Cat#564220; RRID:AB_2869554
bNAb PGT151	Ralf Wagner, Regensburg	N/A
bNAb VRC01	Ralf Wagner, Regensburg	N/A
bNAb 35O22	Ralf Wagner, Regensburg	N/A
bNAb PGT121	Ralf Wagner, Regensburg	N/A
bNAb 2G12	Ralf Wagner, Regensburg	N/A
bNAb PGT122	Ralf Wagner, Regensburg	N/A
bNAb 10E8	Ralf Wagner, Regensburg	N/A
bNAb 447-52D	Ralf Wagner, Regensburg	N/A
bNAb PGT145	Ralf Wagner, Regensburg	N/A
bNAb 17b	Ralf Wagner, Regensburg	N/A
HRP-conjugated goat α-human IgG (H + L)	Jackson ImmunoResearch	Cat#109-035-003; RRID:AB_2337577
Anti-human IgG (Fab’2)	Abcam	Cat#ab87422; RRID:AB_1951105
Anti-V5	Thermo Fisher/Invitrogen	Cat# R961-25; RRID:AB_2556565
Anti-His-TagT	Thermo Fisher/Invitrogen	Cat#R961-25, RRID:AB_2556565

**Bacterial and virus strains**		

Stbl3 Competent Cells	Thermo Fisher Scientific	Cat# C737303
Top10 Competent Cells	Thermo Fisher Scientific	Cat# C40005

**Biological samples**		

Human Peripheral Blood leukocyte reduction system chambers	LMU hospital Munich	N/A
Lamina propria mononuclear cells (LPMC)	U. Dittmer; University of Duisburg-Essen	N/A
Tonsillar cells (HLAC)	LMU hospital Munich	N/A
Anonymized human serum samples	Max von Pettenkofer Institute	N/A

**Chemicals, peptides, and recombinant proteins**		

RPMI 1640 GlutaMAX	Gibco/Thermo Fisher Scientific	Cat#61870044
DMEM GlutaMAX	Gibco/Thermo Fisher Scientific	Cat#31966047
CO_2_-independent medium	Thermo Fischer Scientific	Cat#18045088
MEM Non-Essential Amino Acid	Gibco/Thermo Fisher Scientific	Cat#11140035
Sodium pyruvate	Gibco/Thermo Fisher Scientific	Cat#11360070
Fetal bovine serum	Sigma-Aldrich	Cat#F7524-500ML
Human AB serum	Sigma-Aldrich	Cat#H4522-100ML
Fetal Bovine Serum, ultra-low IgG, US origin	Gibco	Cat#16250078
PBS	Gibco	Cat#12559069
Penicillin-Streptomycin	Sigma-Aldrich	Cat#P0781-100ML
Pancoll	PAN-Biotech	Cat#P04-60500
DMSO	Carl Roth	Cat#4720.2
Accutase	Sigma-Aldrich	Cat#A6964-100ML
Ethylenediaminetetraacetic acid disodiumsalt-dihydrate (EDTA)	Chemsolute, Th. Geyer	Cat#22.161.000
Recombinant human SDF-1α (CXCL12)	Peprotech	Cat#300-28A
Recombinant human RANTES (CCL5)	Peprotech	Cat#300-06
Recombinant human M-CSF	Peprotech	Cat#300-25
Recombinant human GM-CSF	Peprotech	Cat#300-03
Recombinant human IL-4	Peprotech	Cat#200-04
Recombinant human IL-15	Peprotech	Cat#200-15
Recombinant human IL-6	Peprotech	Cat#200-06
Recombinant human IL-2	Peprotech	Cat#200-02
Recombinant human IL-7	Peprotech	Cat#200-07
Recombinant human TNF-α	Peprotech	Cat#300-01A
Recombinant human IL-1β	Peprotech	Cat#200-01b
Recombinant human IFN-γ	Peprotech	Cat#300-02
Phytohemagglutinin (PHA)	Sigma-Aldrich	Cat#L1668-5MG
Liberase TL	Sigma Aldrich	Cat#05401020001
DNAase I	Sigma Aldrich	Cat#04716728001
NLS-Cas9	IDT	Cat#1081059
Accutase	Sigma Aldrich	Cat#A6964-100ML
Heparinase I	New England Biolabs	Cat#P0735S
Heparinase II	New England Biolabs	Cat#P0736S
Heparinase III	New England Biolabs	Cat#P0737S
Chondroitinase ABC from Proteus vulgaris	Merck	Cat#C3667-5UN
Lipopolysaccharide (LPS) from Escherichia coli O55:B5	Sigma Aldrich	Cat#L6529
Probenecid	MP Biomedicals	Cat#02156370-CF
Collagenase D	Sigma-Aldrich	Cat#C5138-100MG
Paraformaldehyde (PFA)	Morphisto	Cat#11762.01000
NuPAGE LDS Sample Buffer (4X)	Invitrogen	Cat#NP0007
Tris Glycine Gels	Thermo Fischer Scientific	Cat#XP00125BOX
InstantBlue protein stain	Merck	Cat#ISB1L-1L
Nitrocellulose membranes	Fisher Scientific	Cat#15259794
Powdered milk	Roth	Cat#T145.2
Clarity™ Western ECL Substrate	Bio-Rad	Cat#1705061
Linear polyethylenimine	Polysciences, Inc	Cat#23966
Lipofectamine 3000	Thermo Fisher Scientific	Cat#L3000008
Maraviroc	Sigma Aldrich	Cat#PZ0002-25MG
Enfuvirtid (T20)	Roche	N/A
AMD3100	Sigma Aldrich	Cat#A5602-5MG
Efavirenz (EFV)	Sigma Aldrich	Cat# SML0536-10MG
HCMV (AD169 strain) gp34	Kolb et al., 2021^49^	N/A
HCMV (AD169 strain) gp68	Kolb et al., 2021^49^	N/A
HCMV/AD169 strain) gp34 mtrp	Kolb et al., 2021^49^	N/A
Human ICOSL	Kolb et al., 2021^49^	N/A
Human ICAM-1	Kolb et al., 2021^49^	N/A
Biotin-XX-conjugated CT-B	Thermo Fisher Scientific	Cat#C34779
AF647-conjugated CT-B	Thermo Fisher Scientific	Cat#C34778
AF594-conjugated streptavidin	Thermo Fisher Scientific	Cat#C34777
Swainsonine	Sigma-Aldrich	S8195
Kifunensine	Sigma-Aldrich	K1140

Other		

EasySep Rosette Human CD4^+^ T cell enrichment kits	STEMCELL	Cat#15062
CD4 T cell Isolation Kit	Miltenyi Biotech	Cat#130-096-533
Human Monocyte Isolation Kit II	Miltenyi Biotech	Cat#130-091-153
CD14 MicroBeads	Miltenyi Biotech	Cat#130-050-201
P3 Primary Cell 4D-Nucleofector™ X Kit S	LONZA	Cat#V4XP-3032
Far Red CellTrace™ Cell Proliferation Kits	Thermo Fisher Scientific	Cat#C34572
Violet CellTrace™ Cell Proliferation Kits	Thermo Fisher Scientific	Cat#C34571
Live/dead™ Fixable Yellow Dead Cell Stain Kit	Thermo Scientific	Cat#L34967
LIVE/DEAD™ Fixable Far Red Dead Cell Stain Kit, for 633 or 635 nm excitation	Thermo Scientific	Cat#L34974
CellTrace CFSE Cell Proliferation Kit	Thermo Scientific	Cat# C34554
ProLong™ Diamond Antifade Mountant	Thermo Scientific	Cat# P36990
fixation/permeabilization solution kit	BD	Cat#554655
Human Cell Surface Marker Screening Panel BD Lyoplate™	BD	Cat#560747
BD Trucount™ Absolute Counting Tubes	BD	Cat#340334
CellTiter-Glo 2.0	Promega	Cat#G9243
CCF2/AM dye	Thermo Fisher Scientific	Cat#K1032
PIERCE BCA assay	Thermo Fischer Scientific	Cat#23225
human albumin/immunoglobulin depletion kit	Merck	Cat#LSKMAGHDKIT
Protein G High Performance Spintrap	Merck	Cat#GE28-9031-34
goat anti-human IgG (Fc Specific)-agarose antibody	Sigma-Aldrich	Cat#A3316
Fluoro Brite DMEM	Gibco	Cat# A18967-01

**Experimental models: Cell lines**		

SupT1	DSMZ	ACC 140
HEK-293T (293T)	DSMZ	ACC 635
HeLa	ATCC	CCL-2

**Oligonucleotides**		

See Table S1 for oligo and gRNAs list		N/A

**Recombinant DNA**		

pCMV6-XL4-CD32A	Origene	Cat#SC112914
pCMV6-XL5-CD32B	Origene	Cat#SC128159
pCMV6-XL5-CD32C	Origene	Cat#SC124933
pCMV-CD64A-GFP	Origene	Cat#RG207487
pCMV-CD16A-GFP	Origene	Cat#RG219204
pBK-CMV-FynN18-GFP	O.T. Fackler, Heidelberg	N/A
pBK-CMV-LckN18-GFP	O.T. Fackler, Heidelberg	N/A
pcDNA3.1 CXCR7	J. Bernhagen, Munich	N/A
pcDNA3.1 CXCR4	J. Bernhagen, Munich	N/A
pCXCR4-HA	J. Bernhagen, Munich	N/A
pCCR5-GFP	This paper	N/A
pHR-CCR5	This paper	N/A
All plasmids encoding CD32 mutants fused to GFP	This paper	N/A
All plasmids encoding CD32 mutants fused to mtagBFP	This paper	N/A
pSAMHD1-GFP	This paper	N/A
pH2B-GFP	This paper	N/A
pLifeAct-GFP	O.T. Fackler, Heidelberg	N/A
pGPI-GFP	V. Laketa, Heidelberg	N/A
pUCIP (HCMV AD169 strain) gp34	Kolb et al., 2021^49^	N/A
pUCIP (HCMV AD169 strain) gp68	Kolb et al., 2021^49^	N/A
pUCIP (HCMV AD169 strain) gp34 mtrp	Kolb et al., 2021^49^	N/A
pLifeAct-mCherry	X. Sewald, Munich	N/A
pNLENG1-IRES	Levy et al., 2004^79^	N/A
pNLENG1-I-70	Levy et al., 2004^79^	N/A
pR5 HIVivo	Horwitz et al., 2017^80^	N/A
pX4 HIVivo	Albanese et al., 2022^20^	N/A
pCMV-BlaM-Vpr	Cavrois et al., 2002^81^	N/A
pcHIV-1 YFP	Barbara Müller, Heidelberg	N/A
pcHIV ΔEnv	Barbara Müller, Heidelberg	N/A
pVpr-GFP	Campbell et al., 2007^82^	N/A

**Software and algorithms**		

Imaris Viewer	Oxford Instruments	https://imaris.oxinst.com/imaris-viewer
ImageJ	National Institutes of Health	https://imagej.net/ij/
NIS Elements	Nikon	https://www.microscope.healthcare.nikon.com/de_EU/products/software/nis-elements
FlowJo	BD	https://www.flowjo.com/
GraphPad Prism v9.30	GraphPad	https://www.graphpad.com/
Adobe Illustrator 2023	Adobe	https://www.adobe.com/de
Biorender.com	Biorender	https://www.biorender.com/

**Other**		

Corning® cell strainer, 40 μM	Corning	Cat#352340
MACS® SmartStrainers, 70 μM	Miltenyi Biotec	Cat#130-098-462
Stericup-GV 0.22 μm, 500 mL	Sigma-Aldrich	Cat#SCGVU05RE
His-Trap FF crude column	GE Healthcare/Cytiva	Cat#17525501
Vivaspin 500 centrifugal concentrators (MWCO 100 kDa)	Merck	Cat#Z614092-25EA
Transwell® Polycarbonatmembran-Zellkultureinsätze (polycarbonate membrane cell culture inserts 6.5 mm Transwell with 3.0 μm pore	Corning	Cat#CLS3415-48EA
μ-Slide 8-well glass bottom	Ibidi	Cat#80827

### Resources availability

#### Lead contact

Further information and requests for resources and reagents should be directed to and will be fulfilled by the lead contact, Prof. Dr. Oliver T. Keppler (keppler@mvp.lmu.de).

#### Materials availability

All unique/stable reagents generated in this study are available from the lead contact with a completed Materials Transfer Agreement.

#### Data and code availability

• All primary microscopy and western blot data generated in this study are available from the lead contact upon request.
• This paper does report original code.
• Any additional information required to reanalyze or reproduce data reported in this paper is available from the lead contact upon request.

### Experimental model and study participants details

#### Isolation of primary human cells from blood

Human CD4 T cells, CD14^+^ monocytes, and CD19^+^ B cells were isolated from heparinized blood retained in leukocyte reduction system chambers from healthy blood donors with approval by the Ethics Committee of the Medical Faculty of LMU München (Project No. 17–202 UE). For CD4 T cells, blood cells were diluted with PBS (Gibco) and CD4 T cells were isolated via the EasySep Rosette Human CD4^+^ T cell enrichment kits (STEMCELL Technologies) according to the manufacturer’s protocol. Alternatively, CD4 T cells were isolated using the CD4^+^ T cell Isolation Kit (Miltenyi Biotech). CD4 T cells were kept in RPMI 1640 GlutaMAX (Gibco) supplemented with 10% (v/v) fetal bovine serum (FBS; Sigma) and Penicillin-Streptomycin (100 IU/mL; Thermo Fisher Scientific). For activation, phytohemagglutinin (PHA; 5 μg/mL; Sigma Aldrich) and IL-2 (50 IU/mL; Biomol) were added to CD4 T cells. Monocytes were isolated via the Human Monocyte Isolation Kit II or CD14 MicroBeads (Miltenyi Biotech) according to the manufacturer’s instructions. B cells were isolated with the CD19 MicroBeads (Miltenyi Biotech).

PBMCs from healthy donors were obtained from leukocyte reduction system chambers as described above and cryoconserved at a concentration of 0.5–1 x 10^8^ cells/ml in RPMI, 10% DMSO, 10% FBS. PBMCs from HIV-1 patients were obtained with informed consent and approval by the local Ethics Committees of the Medical Faculty of LMU München (Project No. 21–0866) and TUM (Project No. 548/21). PBMCs were isolated from EDTA whole blood, and cryoconserved at a concentration of 1 x 10^7^ cells/ml in RPMI, 10% DMSO, 10% FBS.

#### Isolation of lamina propria mononuclear cells

Macroscopically normal human jejunum or ileum tissue samples were obtained from patients undergoing elective abdominal surgery. Lamina propria mononuclear cells (LPMCs) were obtained and processed as described previously.^83^^,^^84^ Briefly, lamina propria mucosa was mechanically separated from muscularis mucosa, EDTA was used to separate epithelial cells, and collagenase D treatment released LPMCs. Cells were cryopreserved in RPMI, 10% DMSO, 10% FBS. This study has been approved by the Ethics Committee of the Medical Faculty of the University Duisburg-Essen (Project No. 15-6310-BO).

#### Isolation of tonsillar cells

Tonsil tissue was removed during therapeutic tonsillectomy from HIV-, hepatitis B virus-, and hepatitis C virus-negative patients with informed consent. Use of anonymous surgical waste for research was approved by the Ethics Committee of the Medical Faculty of LMU München (Project No. 18–209 UE). To prepare single cell suspension, the tissue was cut into blocks of 2–3 mm and passed consecutively through 70 μm and 40 μm cell strainers. If necessary, leftover tissue was additionally incubated with 0.4 mg/mL Liberase TL (Sigma Aldrich) and DNAase I (SIGMA Aldrich) 1 U/μL in RPMI 1640 GlutaMAX (w/o supplements) for 30 min at 37°C shaking, to harvest remaining cells. Cell suspensions were counted and cryoconserved at a concentration of 1 x 10^8^ cells/ml in RPMI 1640, 10% DMSO, 10% FBS.

### Method details

#### Terminal differentiation of monocytes

Monocytes were kept in RPMI 1640 GlutaMAX supplemented with 10% FBS and Penicillin-Streptomycin (100 IU/mL) containing M-CSF (100 ng/mL; Peprotech) for 7 days to differentiate them into monocyte-derived macrophages (MDMs), refreshing the cytokine every 2–3 days. After 6–7 days: For differentiation into M1 macrophages, cells were supplemented with lipopolysaccharide (LPS) (50 ng/mL; Sigma Aldrich) and INF-γ (20 ng/mL; Peprotech). For differentiation into M2 macrophages, cells were kept with IL-4 (20 ng/mL; Peprotech) for one additional day.

For differentiation into monocyte-derived dendritic cells (moDC), monocytes were cultivated with IL-4 (250 IU/mL; Peprotech) and GM-CSF (800 IU/mL; Peprotech) for 7 days, refreshing the cytokines every 2–3 days. After 6–7 days, for differentiation into mature moDCs, DCs were supplemented with IL-6 (2,000 IU/mL; Peprotech), IL-1β (400 IU/mL; Peprotech) and TNFα (2000 IU/mL; Peprotech).

For co-cultures with genetically modified CD4 T cells, autologous monocytes were kept in RPMI 1640 GlutaMAX supplemented with 10% FBS and Penicillin-Streptomycin (100 IU/mL) containing M-CSF (100 ng/mL; Peprotech) for 15 days to differentiate them into MDMs before adding IL-4 (20 ng/mL; Peprotech) for one additional day prior to starting co-culture.

#### Cell lines

Human T cell line SupT1 (DSMZ, ACC 140) was cultivated in RPMI 1640 GlutaMAX (Gibco) supplemented with 10% (v/v) FBS and Penicillin-Streptomycin (100 IU/mL).

IgG-depleted, ultra-low IgG FBS (<5 μg/mL) (Thermo Fisher Scientific) was used for all cell culture experiments except those shown in Figures 1 and 2A–2F and Figures S4 and S5E. 293T cells (DSMZ; ACC 635) were cultivated using DMEM GlutaMAX (Gibco) containing the same additives. HeLa cells (ATCC, CCL-2) were cultivated with the same culture medium as 293T cells with MEM Non-Essential Amino Acid (Gibco). All cells were cultivated at 37°C in a water-saturated atmosphere with 5% CO2.

#### Plasmids

pCMV6-XL4-CD32A (Cat. No. SC112914), pCMV6-XL5-CD32B (Cat. No. SC128159), pCMV6-XL5-CD32C (Cat. No. SC124933), pCMV-CD64A-GFP (Cat. No. RG207487) and pCMV-CD16A-GFP (Cat. No. RG219204) were purchase from Origene. pBK-CMV-FynN18-GFP (encoding only the first 18 amino acid of the N-terminal part (membrane domain) and pBK-CMV-LckN18-GFP (encoding only the first 18 amino acid of the N-terminal part (membrane domain) have been reported.^85^ pcDNA3.1 (+T7) human CXCR7 and pcDNA3.1 (+T7) human CXCR4 were kindly provided by J. Bernhagen, LMU München. To clone plasmids encoding GFP fusion proteins, cDNAs for human CD32A, CD32B, CD32C, CCR5, H2B and SAMHD1 were amplified by PCR and inserted in pEGFP-N1 (Clontech) using *Age*I and *EcoRI*. For site-specific mutagenesis, two overlapping primers carrying the mutations were used to amplify the plasmids followed by *Dpn*I digestion. To clone mtagBFP fusion proteins for human CD32A, CD32B, CD32C, and the corresponding mutated isoforms, the receptor encoding insert was cloned from the corresponding GFP fusion protein plasmids by restriction digest with AgeI and *EcoR*I pCMV-mtag BFP. For cloning of pCCR5-GFP, human CCR5 was amplified by PCR and inserted in pEGFP-N1 (Clontech) using *Sal*I and *Nhe*I. All oligos used for amplification are listed in the Table S1.

#### Knockout generation in primary CD4 T cells and monocytes

Following our recently established workflow,^20^ freshly isolated CD4 T cells or CD14^+^ monocytes (2x10^6^) were washed twice with PBS and resuspended in 20 μL buffer P3 (Lonza). In parallel, synthetic sgRNAs (Synthego) were incubated together with recombinant NLS-Cas9 (IDT) for 10 min at room temperature at a ratio of 1:2.5 (40 pmol Cas9 protein per 100 pmol gRNA) to form the CRISPR-Cas9-gRNA ribonucleoproteins (RNP) complex. The Cas9-gRNA mix was diluted with sterile filtered (0.22 μm) PBS to reach a final concentration of 20 μM RNPs. For efficient KO of individual targets, a mix of two or three gRNA was used, depending on the specific gene. Here, 2 μL of RNP complexes for each gRNAs were mixed with the cell suspension and transferred into a 16-well reaction cuvette of the 4D-Nucleofector System (Lonza). Cells were nucleofected using program EH-100 on the 4D-Nucleofector system. 100 μL of pre-warmed RPMI (w/o supplements) was added to each well, cells were transferred to a 48-well plate and allowed to recover for 10 min at 37°C. Subsequently, complete culture medium supplemented with the corresponding cytokines were added. A list of gRNA sequences used in this study can be found in Table S1. Knockouts were verified by Sanger sequencing as reported.^20^

#### Co-culture between macrophages and CD4 T cells

PBMCs from healthy blood donors were isolated with 1.077 g/mL Pancoll (Pan-Biotech) and density gradient centrifugation. CD14^+^ monocytes were isolated with CD14 MicroBeads (Miltenyi Biotech), and the remaining part of PBMCs were cryopreserved. After isolation, CD14^+^ monocytes were differentiated as outlined above. On day 7–9, autologous PBMCs were thawed and CD4 T cells were isolated with a CD4 T cell isolation kit (Miltenyi Biotec). CD4 T cells were stained with Violet or Far Red CellTrace Cell Proliferation Kits (Thermo Fisher Scientific) following the manufacturer’s protocol. Finally, cells were washed three times and resuspended in RPMI 1640 GlutaMAX complete, counted and used for the experiment. Stained CD4 T cells were placed on top of differentiated macrophages (day 7–9) with a ratio of 1:2 (MDM: CD4 T cells), if not specified differently. For trogocytosis enhancement assays, M2 macrophages were treated with Alemtuzumab (0.2 μg/mL, kindly provided by the Central Cytostatics Preparation Facility, LMU hospital Munich) or Ultra-LEAF Purified Human IgG1 (BioLegend) in RPMI 1640 GlutaMAX complete, before adding the CD4 T cells suspension on top, resulting in a final antibody concentration of 0.1 μg/mL. After 2 days (if not specified differently), CD4 T cells were collected and used for subsequent experiments. If purified CD4 T cells or an enriched CD4 T cell sub-populations were needed (i.e., CD32-positive or negative cells), CD4 T cells were sorted following co-culture using a FACSAria Fusion cell sorter (BD).

#### Plasmid nucleofection of CD4 T cells

Freshly isolated CD4 T cells (1x10^6^) were washed twice with PBS and resuspended in 20 μL buffer P3 (Lonza) with 0.1–0.5 μg plasmid DNA of the corresponding expression vector and transferred into a 16-well reaction cuvette of the 4D-Nucleofector System (Lonza). Cells were nucleofected using program EO-115. After nucleofection, 100 μL of pre-warmed RPMI (w/o supplements) was added to each well and cells were transferred to a 48-well plate and allowed to recover for 10 min at 37°C. Subsequently, complete culture medium supplemented with the corresponding cytokines were added. After 24 h, the nucleofection efficiency was analyzed by antibody staining for the corresponding markers, and 200,000 cells were seeded into 96-well V-shape bottom plates to perform HIV-1 fusion assays.

#### Immunoblotting

To analyze the IgG content in human sera, 10 μL of human serum, flowthrough of IgG-depletion columns, washing fraction or 2 μg of purified IgGs were mixed with reduced LDS sample buffer (Pierce) and incubated at 90°C for 10 min. For analysis of Fab and Fc of human IgGs, 2 μg of each antibody was mixed with non-reduced LDS sample buffer without heating. Sample lysates were separated by tris-glycine denaturing or non-denaturing SDS-PAGE (Thermo Fischer Scientific). Gels were then stained with InstantBlue protein stain (Sigma-Aldrich) or blotted onto 0.2 mm nitrocellulose membranes (GE Healthcare). After blocking in 5% milk (Roth) in TBS-T for 1 h, membranes were incubated with HRP-conjugated goat α-human IgG (H + L) (Cat. No. 109-035-003, Jackson ImmunoResearch), α-human IgG (Fab’2) (Cat. No. ab87422, Abcam), or α-human IgG (Fc-specific) (Cat. No. A0170, Sigma-Aldrich) antibodies in 5% milk for 1 h (1:10,000). ECL (ThermoFisher Scientific) was used as substrate and the chemiluminescent signals were detected on a Fusion Fx (Vilber).

#### Flow cytometry and antibodies

Macrophages were washed once with PBS and kept in Accutase (Innovative Cell Technologies, Inc.) for 1 h at 37°C to detach them. All cells were collected, washed once with PBS and resuspended in 25 μL of blocking solution consisting of PBS, 2 mM EDTA and 5% human AB serum (Sigma-Aldrich) and kept for 10 min at 4°C. After this time, 25 μL of staining solution (FACS buffer (PBS, 2 mM EDTA, 1% FBS) and specific antibodies) were added, and kept for 20 min at 4°C. Next, cells are washed and resuspended in FACS buffer (100 μL). For intracellular FACS staining, SupT1 cells were first stained with CellTrace Violet (Thermo Fisher Scientific) and co-cultured with 293T cells expressing CD32A-GFP or CD32B-GFP. After 24 h, cells were fixed and permeabilized with fixation/permeabilization solution kit (BD) following the manufacturer’s protocol. After staining with α-GFP antibodies as described above, cells were analyzed on a FACSLyric (BD).

The following human-specific antibodies were used: α-CXCR4 (BV421 [Cat. No. 562448] BD, APC [Cat. No. 555976] and PE-Cy5 [Cat. No. 555975] clone 12G5, BD; PE-Cy7 [Cat. No. 306514] clone 12G5, Biolegend), α-CXCR2 (APC [Cat. No. 551127] clone 6C6, BD), α-CXCR7 (APC [Cat. No. 391405] clone 10D1-J16, BioLegend), α-CD25 (BV421 [Cat. No. 562442] and APC [Cat. No. 555434] clone M-A251, BD), α-CD69 (BV421 [Cat. No. 562884] and APC [Cat. No. 555533] clone FN50, BD), α-CD32 (PerCP-Cy5.5 [Cat. No. 303216], PE-Cy7 [Cat. No. 303214] and AF647 [Cat. No. 303212] clone, FUN-2, BioLegend; PE [Cat. No. 2116030] clone FUN-2, Sony Biotechnology; α-GFP (AF647 [Cat. No. 338005] clone FM2-64G, BioLegend), α-CCR5 (APC [Cat. No. 556903], clone 2D7, BD), α-human IgG Fc (AF647 [Cat. No. 409320] clone HP6017, BioLegend), F(ab’)2 Goat α-human IgG Fcγ (PE [Cat. No. 398004] polyclonal, BioLegend; APC [Cat. No. 109-136-170] polyclonal, Jackson ImmunoResearch), α-CD11b (APC [Cat. No. 17-0118-42] clone ICRF44, eBioscience; FITC [Cat. No. 301330] and Bv421 [Cat. No. 301324] clone ICRF44, BioLegend), α-HLA-DR (FITC [Cat. No. 347363] clone L243, BD), α-CD209, (BV421 [Cat. No.330118] clone 9E9A8, Biolegend), α-CD206 (APC [Cat. No. 550889], clone 19.2, BD), α-CD11a (APC [Cat.:301212] clone HI111, Biolegend), α-CD11c (FITC [Cat. No.337213] clone Bu15, Biolegend), and Human Cell Surface Marker Screening Panel (Cat. No. 560747, BD). Stained cell suspensions were analyzed with the BD FACS Lyric (Becton, Dickinson and Company; BD) and using FlowJo software (BD).

The following procedure for the flow cytometric analysis of PBMCs from healthy donors and patients living with HIV-1 as well as cells from lymphatic tissue (tonsil, lamina propria tissue) was used: On the day of analysis, cells were thawed and washed twice with PBS before resuspending 1x10^6^ cells in 50 μL of blocking solution consisting of PBS, 2 mM EDTA and 5% human AB serum (Sigma-Aldrich) and kept for 10 min at 4°C. After this time, 50 μL of staining solution (FACS buffer (PBS, 2 mM EDTA, 1% FBS) with addition of Live/dead Fixable Yellow Dead Cell Stain Kit [Cat. No. L34967, Thermo Scientific] and the antibody panel α-CD14 (FITC [Cat. No. 325603] clone HCD14, BioLegend), α-CD19 (FITC [Cat. No. 302206] clone HIB19, Biolegend), α-CD3 (APC-Cy7 [Cat. No. 557832] clone SK7, BD) α-CD4 (PE-Cy7 [Cat. No. 300512] Biolegend, APC [Cat. No. 555349] BD, clone RPA-T4), α-CD32 Bv421 [Cat. No. 564838] clone FLI8.26, BD), were added, and kept for 20 min at 4°C. Finally, cells were washed twice and resuspended in FACS buffer (100 μL) and analyzed with the BD FACS Lyric.

#### Human cell surface marker screening

CD14^+^ monocytes and CD4 T cells were isolated from PBMCs, differentiated, co-cultured for 48 h, and CD4 T cells were sorted as described above. After sorting, the surface receptors of CD4 T cells were stained with the BD Lyoplate (BD) consisting of a panel of 242 monoclonal primary antibodies and Alexa Flour 647-conjugated goat anti-mouse IgG and goat anti-rat IgG secondary antibodies, and subsequently analyzed by flow cytometry. In parallel, M2 cultures were analyzed with the same antibody panel. Figure 1A was generated using the ggplot2 package.

#### Cell labeling

The following dyes were used: CellTrace CFSE Cell Proliferation Kit, and violet and far red CellTrace Cell Proliferation Kits (Thermo Fisher Scientific) following the manufacturer protocol. Finally, cells were washed three times and resuspended in RPMI 1640 GlutaMAX complete, counted and used for the experiment.

#### Co-culture between 293T cells and SupT1 cells/primary CD4 T cells

On day 1, 520 ng of the desired expression plasmids were mixed with 1 μL linear polyethylenimine (Merck) and 100 μL DMEM only, followed by incubation of the mixture with 90% confluent 293T cells in a 24-well plate for overnight. On day 2, 293T cells were reseeded at 1 x 10^5^ cells per well of a 96-well plate and incubated overnight. On day 3, SupT1 cells or freshly isolated CD4 T cells were stained with CellTrace and added at 2.5 x 10^5^ cells per well to these 293T cells. Subsequent analyses were performed after co-cultivation for 24 h.

#### Expression and purification of soluble V5-His6-tagged HCMV vFcγR ectodomain proteins

Expression and purification were performed in principle as reported.^49^ Only extracellular domains (ECDs) of the viral Fc-binding proteins gp34 and gp68 from HCMV AD169 strain were recombinantly expressed. In addition, an impaired-Fc-binding version of gp34, gp34 mtrp (mutated tryptophan at position 65, W65F) and soluble human ICOSL, serving as a non-Fc-binding control protein, were used. For expression, detection and purification purposes, soluble proteins were in-frame V5-His tagged on the C-terminal part of the ectodomains. gp34 and gp68 were cloned based on cDNA from HCMV AD169 BAC-infected MRC5 fibroblasts. For cloning purpose, restriction sites were introduced by PCR amplification. Recombinant protein expression was conducted by two different expression strategies: (i) by transient plasmid transfection of 293T cells (using polyethylemine (PEI, branched, Sigma-Aldrich, Germany) or (ii) by lentiviral transduction of 293T cells using pUCIP plasmids and puromycin selection (2 mg/mL). When transiently or stably transfected cells were 90–100% confluent and well attached, medium was carefully replaced by starvation medium without FBS (DMEM w/o phenol red, 1% penicillin/streptomycin, 1% sodium pyruvate). After 5–7 days or when cells started to detach, supernatants were collected, remaining cells in the supernatant removed by centrifugation (40 min, 4,000 g), sterile filtered and adjusted to a 10 mM imidazole concentration and passed over a His-Trap FF crude column (GE Healthcare, USA). Proteins were eluted in imidazole/phosphate buffer (250 mM Imidazole, 20 mM sodium phosphate, 500 mM NaCl) and dialyzed against PBS and concentrated via Amicon columns. Protein concentrations were determined by PIERCE BCA assay (Thermo Fischer Scientific) and protein quality was analyzed in various assays like IgG-Fc binding ELISA, Coomassie staining and western blotting using anti-V5 (Invitrogen, USA) or anti-His-Tag (Bethyl Laboratories Inc.) antibodies for detection.

#### Boosting or inhibiting trogocytosis

293T and SupT1 cells were prepared as donor cells and recipient cells, respectively, as described above. Before co-culture, donor cells were treated with human serum samples, bNAbs of HIV, α-CD32 antibodies or other antibodies indicated for 30 min at the indicated concentrations. Anonymized human serum samples were obtained from the Diagnostic Department of the Max von Pettenkofer Institute with approval by the local Ethics Committee. Serum was heat-inactivated at 56°C for 1 h. HIV-1 viral loads of patients on ART were all less than 50 copies/ml. The viral loads of patients without ART are listed in Table S3. During co-culture, human sera were added at 10% in complete culture medium. bNAbs and α-CD32 antibodies were used as 2.5 μg/mL. For inhibition, the following antibodies were used: α-CD32 (Cat. No. 303202, clone FUN-2, BioLegend; Cat. No. 557333, clone FL8.26, BD; Cat. No. 60012, clone IV-3, Stemcell Technologies) and mouse IgG1 isotype control (Cat. No. 400102, clone MOPC-21, BioLegend). After pre-incubation, the recipient cells were added to donor cells and co-cultured for 24 h.

#### IgG depletion from serum

IgGs were depleted and eluted from selected serum samples with either (i) human albumin/immunoglobulin depletion kit (Merck), (ii) Protein G High Performance Spintrap (Merck) or (iii) goat anti-human IgG (Fc Specific)-agarose antibody (Merck). The flowthrough was collected for further analysis. IgGs were eluted with 0.2 M glycine-HCl pH 2.2 and were desalted and concentrated with Vivaspin 500 centrifugal concentrators MWCO 100 kDa (Sartorius). The protein concentration in the eluate was quantified using the BCA assay (Pierce). The eluate of the human albumin/immunoglobulin depletion kit (Figure 3C) was used at 1.4 mg/mL in the co-culture system, the eluate of the Protein G High Performance Spintrap and goat anti-human IgG was used at 171 μg/mL (Figures S8C and S8D).

#### Human IgG binding to CD4 T cells

Primary CD4 T cells were isolated as described above. After isolation, resting CD4 T cells were incubated with FcBlock ([Cat. No. 564220], clone Fc1.3216, BD) for 20 min. After removing the supernatant, CD4 T cells were incubated with 50 μL selected human sera, 10 μg/mL bNAbs or 0.05 μg/mL Alemtuzumab. Excess primary antibodies were washed away with FACS buffer, and F(ab')2 fragment goat anti-human IgG, Fcγ fragment-specific (APC [Cat. No. 109-136-170] polyclonal, Jackson ImmunoResearch) was used as secondary antibody.

#### bNAb-hCMV glycoprotein trapping assay

The preparation of glycoproteins gp34, gp34mtrp and gp68 is described above. bNAb PGT151 (0.6 μM) was incubated together with titrated amounts of the purified soluble glycoproteins (gp) for 30 min at 37°C in RPMI 1640 GlutaMAX (w/o FBS and Pen-Strep). Next, the PGT151/hCMV gp pre-treatment was added to 293T cells expressing CD32B-BFP. After 30 min at 37°C, the co-culture with SupT1 was started as described above, with a final concentration of 0.06 μM for PGT151. The next day, BFP positivity of SupT1 cells was analyzed by flow cytometry.

#### Chemokine-migration assay

CD4 T cells were used as recipient cells one week after nucleofection and stained with CellTrace as described above. As donor cells, HeLa cells were transfected with CD32B-GFP or H2B-GFP, together with pHR-CCR5 or CXCR4-HA, with Lipofectamine 3000 (Invitrogen) following the manufacturer’s protocol. The recipient cells and donor cells were co-cultured for 24 h in a 96-well plate. After removing the membrane from the Transwell system, 500 μL of RPMI medium supplemented with 0.2% FBS and the chemokine SDF-1α (1,000 ng/mL; Peprotech) or RANTES (800 ng/mL; Peprotech) were added at the bottom of the Transwell-24 well plate with 3.0 μm pore polycarbonate membrane insert (Corning). The membrane was added into the corresponding wells, and for each condition 200 μL of medium containing 2.5 x 10^5^ cells were transferred on top of the membrane. The 24-well plate was incubated at 37°C for 3 h. Subsequently, the membrane was removed and the total number of cells in the 500 μL medium on the bottom of the Transwell was quantified by flow cytometry. For quantification BD Trucount Absolute Counting Tubes were used.

#### ICAM-1 adhesion assay

This assay was adapted from Strazza et al*.*^42^ A 384-well plate was coated with 10 μg/mL ICAM-1 solution (1 mM CaCl_2_ and 2 nM MgCl_2_ in PBS) at 37°C for 1 h. The plate was kept at 4°C prior to use. CD4 T cells were stained with CellTrace and co-cultured with autologous M2. After 2 days, CD4 T cells were sorted and resuspended in adhesion solution (0.5% BSA, 1 mM CaCl_2_ and 2 nM MgCl_2_ in PBS). 2 x 10^4^ cells were seeded into each well and incubated at 37°C for 30 min. After incubation, each well was washed with 30 μL adhesion solution three times. 30 μL adhesion solution and 10 μL CellTiter-Glo 2.0 solution (Promega) was added to each well. The number of cells remaining in each well was quantified with a CLARIOstar Plus plate reader (BMG Labtech).

#### HIV-1 plasmids

For HIV infection, the HIV-1 GFP proviral clone NLENG1-IRES^79^ and NLENG1-I-70^79^ was used, referred to as X4 or R5 HIV-1 GFP in the current study. For the virion-fusion assay, the R5 HIV-1 proviral clone HIVivo,^80^ kindly provided by Michel Nussenzweig, was used in combination with pCMV-BlaM-Vpr during virus production (see below). For the HIV-1 binding assay the X4 tropic pcHIV-1 YFP^86^ or pcHIV ΔEnv (kindly provided by Barbara Mueller) was used in combination with Vpr-GFP. X4 HIVivo was generated introducing the envelope gene from NLENG1-IRES into the R5 HIVivo backbone using the restriction sites *EcoR*I and *Hpa*I.

#### HIV-1 production

To produce sucrose cushion-purified HIV-1 stocks.^87^ 293T cells were seeded at a density of 8 × 10^6^ cells in a 15-cm dish. After 24 h, cells were co-transfected with a mixture of 37.5 μg HIV-1 plasmid and 112.5 μL of L-PEI (3 μL of L-PEI for every μg of DNA; stock concentration of 1 μg/μL, Polysciences, Inc) in DMEM without any additives for 30 min. After this time, the DNA/PEI solution was added on top of the cells. After 72 h, the supernatant was harvested and virus was purified via 25% sucrose-cushion centrifugation. For virus production for the virion-fusion assay, the transfection was performed as described above, combining 37.5 μg of X4 or R5 HIVivo and 12.5 μg of pCMV-BlaM-Vpr.

#### HIV-1 fusion assay

The assay was conducted using the Vpr-BlaM assay.^88^ Proviral plasmids R5 HIVivo^80^ or X4 HIVivo^20^ were used in combination with pCMV-BlaM-Vpr during virus production. CD4 T cells were incubated with virions containing BlaM-Vpr at 37°C for 4 h. Subsequently, cells were washed twice in CO_2_-independent medium (Thermo Fischer Scientific), and then loaded with CCF2/AM dye (Thermo Fisher Scientific), as described before.^28^^,^^88^ Briefly, 2 μL of CCF2/AM (1 mM) was mixed with 8 μL of solution B, and 10 μL of probenecid (250 mM stock; MP Biomedicals) in 1 mL of CO_2_-independent medium supplemented with 10% FBS. Cells were incubated in 100 μL of loading solution for 16 h at RT. Cells were then washed twice with PBS and fixed with 4% (v/v) paraformaldehyde (PFA) for 1.5 h. Subsequently, cells were washed and resuspended in FACS buffer (PBS, 1%FBS, 2mM EDTA). The shift in emission fluorescence of CCF2 after cleavage was monitored by flow cytometry.

#### HIV-1 infection assay

The titer of individual virus stocks was determined on SupT1 cells using the virus-encoded GFP signals measured by flow cytometry as readout for productive infection. Resting CD4 T cells were infected with virus stocks at different multiplicities of infection (MOIs) as indicated for each experiment. After 3 days, cells were washed twice with PBS and fixed with 4% PFA for 1.5 h. Cells were then washed and resuspended in FACS buffer. The percentage of GFP-positive cells was quantified by flow cytometry. Drug or antibody controls were added to cells 30 min prior to HIV-1 challenge. The following drugs were used: Efavirenz (EFV) (stock: 10 mM; Sigma Aldrich), AMD3100 (stock: 16 μg/mL; Sigma Aldrich), Anti-CD4 clone SK3 (stock: 25 μg/mL; Biolegend), T20 (stock: 90 mg/ml; Enfuvirtid; Roche), and Maraviroc (10 μg/μL; Sigma Aldrich).

#### HIV-1 Vpr-GFP binding assay

In a 15 cm plate dish, 10 x 10^7^ 293T cells were transfected with 17 μg of each plasmid encoding Vpr-GFP and pHIV-YFP and the supernatant collected and concentrated with ultracentrifugation as described above. CD4 T cells co-cultured or not with M2 macrophages were sorted as described above. After sorting, 200,000 CD4 T cells were seeded in a V-bottom 96-well plate (Corning) and inoculated with indicated titer of HIV-1 Vpr-GFP virus at 16°C for 1 h if not indicated otherwise. Depending of the experimental set up cells were treated before the virus inoculation with antiviral drugs, antibodies (final conc. 100 ng/μL) or medium only for 15 min at 16°C. The Following antibodies were used: α-CD4 [Cat.No. 344602] clone SK3, Biolegend; [Cat. No.555344] clone RPA-T4, BD; [Cat. No. 556614] clone M-T477, BD; [Cat. No. 550625] clone L200, BD; α-CD11b [Cat. No. sc-59744] clone VIM12, Santa Cruz Biotechnology; [Cat. No. 301361] clone ICRF44, Biolegend; α-CD11c [Cat. No. 337202] clone Bu15, Biolegend; α-CD206 [Cat. No.555953] clone 19.2, BD; [Cat. No. 321149] clone 15-2, Biolegend; α-CD209 [Cat. No. 30835-MSM1-P1ABX] clone C209, 1781, Thermo Fischer Scientific, [Cat. No. MAB16211], R&D System; α-CD13 [Cat. No. 301723] clone WM15, Biolegend; α-CD226 [Cat. No. 337102] clone TX25, Biolegend; α-CD74 [Cat. No. 326802] clone LN2, Biolegend; α-CD54 [Cat. No 0.322721] clone HCD54, Biolegend; α-CD227 [Cat. No. 355602] clone 16A, Biolegend; α-HLA-DR [Cat. No. 307665] clone L243, Biolegend; Biolegend; mouse IgG1κ [Cat. No. 400102], Biolegend; mouse IgG2a [Cat. No. 401501], Biolegend; mouse IgG2b [Cat. No. 400302], Biolegend.

The enzymatic digestion with 1 U/ml of Heparinase I/II/III (Heparinase I [Cat. No. P0735S]; Heparinase II [Cat. No. P0736S]; Heparinase III [Cat. No. P0737S]; New England Biolabs) and Chondroitinase (Chondroitinase ABC from Proteus vulgaris [Cat. No. C3667-5UN] Merck) was performed for 15 min at 37°C in PBS stopping the reaction by addition of medium supplemented with 10% FBS followed by a washing step with PBS, prior to virus inoculation. For all HIV-1 binding assays the culture medium was CO_2_-independent medium (Thermo Fischer Scientific). After intensive washing with FACS buffer, cells were stained for CD32 or other receptors of interest for flow cytometer analysis as described above and finally fixed with 4% (v/v) PFA at room temperature for 10 min. The result was acquired with flow cytometry or by microscopy (see below).

#### Confocal microscopy

293T cells were co-transfected with CD32B-GFP and pHR-CCR5. SupT1 cells transduced with a lentiviral vector encoding LifeAct-mCherry were used as donor cells. bNAb PGT151 was added to boost trogocytosis. After co-culture, cells were collected, stained with α-CCR5 mAb (clone 2D7, BD) and anti-mouse IgG secondary antibody (Invitrogen), and fixed with 4% (v/v) PFA. For HIV binding assay, HIV-1 Vpr-GFP was incubated with CD4 T cells as described above. After inoculation, cells were collected, washed twice with PBS and resuspended in staining solution (50 μL), i.e., FACS buffer (PBS, 1% FBS, 2 mM EDTA) containing α-CD32 mAb (AF647 [Cat. No. 303212] clone FUN-2, Biolegend) and α-CD4 mAb (AF594 [Cat. No. 300544] clone RPA-T4, Biolegend) and kept for 20 min at 4°C. After this time, cells were washed twice and fixed with 4% (v/v) PFA at room temperature for 10 min and washed again. Cells were then mounted with ProLong Diamond Antifade Mountant (Thermo Fischer Scientific). 3D images were taken by a spinning disk confocal microscope (CSU-W1, Nikon) with 0.3 μm stepsizes. The software Imaris Viewer (Oxford Instruments) was used to analyze images. Intensity profiles were obtained by using ImageJ (National Institutes of Health) software.

Colocalization was analyzed using a custom script written in jupyterlab 2.2.6 for Python 3.8.5, and the following packages: numpy 1.19.4, tiff file 2020.12.8 and scikit-image 0.18.1. Colocalization sites were detected as the pixels concomitantly in the 96.5th percentiles of cell, receptor and virus particle equalized histogram distributions. They were Gaussian blurred and quantified using a Laplacian of the Gaussian method for blob detection. For the control group the planes of the receptor stacks and, separately, those of the virus particle stacks were randomly re-shuffled, within each stack, before undergoing a new colocalization detection and quantification. The parameters for accurate blob detection were determined manually. The same parameters were used for the data and the matched shuffled controls. One stack was identified as outlier (ROUT method, Q = 10%) and excluded from the following statistical analysis.

#### Monosialotetrahexosylganglioside (GM1) labeling

Primary CD14^+^ monocytes and CD4 T cells were isolated and differentiated as described above. Cholera toxin subunit B (CT-B) staining solution was prepared by dissolving 1 μg/mL CT-B-conjugate in complete medium. Before co-culture, differentiated macrophages were rinsed once with cold complete medium, and then incubated for every 100,000 cells in 200 μL staining solution for 10 min at 4°C, followed by three gentle washes with cold PBS. After labeling, macrophages were co-cultured with CD4 T cells and boosted with mAb Alemtuzumab as described above. As a control, CD4 T cells were also incubated with supernatant of CT-B-labeled macrophages. For flow cytometry, AF647-conjugated CT-B (Thermo Fischer Scientific) and for microscopy, biotin-XX-conjugated CT-B (Thermo Fischer Scientific) was used. After co-culture for two days, CD4 T cells were sorted and incubated with HIV-1 Vpr-GFP, stained with α-CD32 mAb conjugated to PE-Cy7 (flow cytometry) or AF647 (microscope), and AF594-conjugated streptavidin (Thermo Fischer Scientific) (microscope), fixed and acquired with flow cytometry and confocal microscopy as described above. Intensity profiles were obtained by using ImageJ (National Institutes of Health) software.

#### Live microscopy

293T cells were seeded in a 24-well plate with a density of 200,000 cells per well and transfected the next day with PEI (each well with 320 ng of either pCD32B-GFP, pLifeAct-GFP, pGPI-GFP or pCD32B ΔCT-GFP as controls). 24 h post transfection 20,000 transfected 293T cells were seeded on fibronectin-coated 8-well ibidi glass bottom slides and after 48 h SupT1-LifAct-mCherry cells were added in a ratio of 1:3 in 250 μL co-culture medium (1.2 mL Fluoro Brite DMEM (gibco A18967-01), 3.6 mL RPMI, 10% IgG low FBS, 1% Pen/Strep). Cell were then cultured or not with bNAb PGT151 (2.5 μg/mL) and imaged using spinning disc microscopy. Spinning disk confocal microscopy was performed using a Nikon Ti2 microscope equipped with an Andor CSU-W1 spinning disk head. An Apochromat TIRF 100x/N.A. 1.49 oil immersion objective and EMCCD Andor iXon DU-888 camera were used. Live-cell imaging was performed at 37°C and 5% CO_2_. Images were acquired at 10 selected positions using an automated stage and the Perfect Focus System (PFS) for focus stabilization with a time-resolution of 80s/stack for 4h. To maximize acquisition speed images of the GFP (488 nm laser excitation; 525/50 bandpass filter for detection) and mCherry signals (561 nm laser excitation, 600/50 bandpass filter for detection) were acquired simultaneously using two cameras and a 561LP beam splitter. Stacks were acquired with a z-spacing of 500 nm covering axial range between 0 μm and 30 μm above the coverslip. To further increase the acquisition speed, minimize potential light-induced cytotoxic effects and enable long-term imaging, we aimed to use absolutely minimal camera exposure times and laser powers. This inevitably resulted in acquisition of very low signal-to-noise (SNR) images. To be able to use these images for subsequent visualization and analysis we made use of a machine learning-based approach (content-aware image restoration (CARE)) that restores high SNR from low SNR data.^28^ We used the CARE-based approach as it is implemented in the Nikon microscope control and analysis software (NIS Elements) as part of the NIS.ai “restoration” module. In short, a set of corresponding images was sequentially recorded with high laser power/long camera exposure and low laser power/short camera exposure. Next, the machine learning model has been trained that restores low SNR images to high SNR images using high laser power/long camera exposure data as the ground truth. The model was then applied to low SNR signals from live imaging and high SNR images were restored. This procedure allowed us to expose cells with 60-fold lower illumination than we would normally use to obtain high SNR images and therefore enabled us to perform long time-lapse imaging at high spatiotemporal resolution.

To enable better visual appreciation of these complex datasets we segmented the GFP signal from the background. For segmentation we trained a Random Forest classifier using the software ilastik^89^ and its pixel classification workflow. The training set of data was arbitrary selected and very sparsely labeled (<0.1% of total pixels were manually categorized into “signal” and “background” categories). The classifier was then applied to all the z slices of the entire time-lapse. Next, the segmented data from the GFP channel was imported in the software Imaris (Bitplane, Oxford Instruments) for 3D visualization. mCherry data was imported in Imaris as a second channel and displayed as a maximum intensity projection along the viewing angle. In order to better focus our attention on the membrane transfer events between 293T and SupT1 cells and remove distracting GFP objects sticking to the coverslip we used a filter in Imaris that removes all GFP objects whose center is closer than 800 nm from the coverslip.

#### Imaging flow cytometry

Lymphocytes obtained from peripheral blood and tonsils were stained with fixable near-IR dead cell stain kit (Invitrogen), α-CD3, α-CD4, α-HLA-DR and α-CD32 antibodies. The images were acquired with Amnis Imagestream imaging flow cytometer.

### Quantification and statistical analysis

#### Statistical analyses

All data were analyzed using GraphPad Prism v9.30. Statistical tests are indicated in the figure legends. N indicates a biological replicate in experiments performed with only cell lines and the number of donors for experiments performed with primary cells.

## Supplemental information

Document S1. Figures S1–S17 and Tables S2 and S3

Table S1. gRNA and cloning PCR primer sequences, related to STAR Methods

Document S2. Article plus supplemental information

## References

[1] Zola H., Swart B., Banham A., Barry S., Beare A., Bensussan A., Boumsell L., D Buckley C., Bühring H.J., Clark G. (2007). CD molecules 2006--human cell differentiation molecules. J. Immunol. Methods.

[2] Tatari-Calderone Z., Semnani R.T., Nutman T.B., Schlom J., Sabzevari H. (2002). Acquisition of CD80 by human T cells at early stages of activation: functional involvement of CD80 acquisition in T cell to T cell interaction. J. Immunol..

[3] Game D.S., Rogers N.J., Lechler R.I. (2005). Acquisition of HLA-DR and costimulatory molecules by T cells from allogeneic antigen presenting cells. Am. J. Transplant..

[4] Baba E., Takahashi Y., Lichtenfeld J., Tanaka R., Yoshida A., Sugamura K., Yamamoto N., Tanaka Y. (2001). Functional CD4 T cells after intercellular molecular transfer of 0X40 ligand. J. Immunol..

[5] Evans H.M., Schultz D.F., Boiman A.J., McKell M.C., Qualls J.E., Deepe G.S. (2021). Restraint of Fumarate Accrual by HIF-1α Preserves miR-27a-Mediated Limitation of Interleukin 10 during Infection of Macrophages by Histoplasma capsulatum. mBio.

[6] Suzuki Y., Yoshida T., Wang G., Aoki T., Katayama T., Miyamoto S., Miyazaki K., Iwabuchi K., Danbara M., Nakayama M. (2013). Incidence and clinical significance of aberrant T-cell marker expression on diffuse large B-cell lymphoma cells. Acta Haematol..

[7] Patel D.M., Arnold P.Y., White G.A., Nardella J.P., Mannie M.D. (1999). Class II MHC/peptide complexes are released from APC and are acquired by T cell responders during specific antigen recognition. J. Immunol..

[8] Kedl R.M., Schaefer B.C., Kappler J.W., Marrack P. (2002). T cells down-modulate peptide-MHC complexes on APCs in vivo. Nat. Immunol..

[9] Qureshi O.S., Zheng Y., Nakamura K., Attridge K., Manzotti C., Schmidt E.M., Baker J., Jeffery L.E., Kaur S., Briggs Z. (2011). Trans-endocytosis of CD80 and CD86: a molecular basis for the cell-extrinsic function of CTLA-4. Science.

[10] Wakim L.M., Bevan M.J. (2011). Cross-dressed dendritic cells drive memory CD8+ T-cell activation after viral infection. Nature.

[11] Schriek P., Villadangos J.A. (2023). Trogocytosis and cross-dressing in antigen presentation. Curr. Opin. Immunol..

[12] Zhao S., Zhang L., Xiang S., Hu Y., Wu Z., Shen J. (2022). Gnawing Between Cells and Cells in the Immune System: Friend or Foe? A Review of Trogocytosis. Front. Immunol..

[13] Chauveau A., Aucher A., Eissmann P., Vivier E., Davis D.M. (2010). Membrane nanotubes facilitate long-distance interactions between natural killer cells and target cells. Proc. Natl. Acad. Sci. USA.

[14] Kim H.R., Mun Y., Lee K.S., Park Y.J., Park J.S., Park J.H., Jeon B.N., Kim C.H., Jun Y., Hyun Y.M. (2018). T cell microvilli constitute immunological synaptosomes that carry messages to antigen-presenting cells. Nat. Commun..

[15] Stinchcombe J.C., Asano Y., Kaufman C.J.G., Böhlig K., Peddie C.J., Collinson L.M., Nadler A., Griffiths G.M. (2023). Ectocytosis renders T cell receptor signaling self-limiting at the immune synapse. Science.

[16] Park J.S., Kim J.H., Soh W.C., Kim N.Y., Lee K.S., Kim C.H., Chung I.J., Lee S., Kim H.R., Jun C.D. (2023). Trogocytic molting of T cell microvilli upregulates T cell receptor surface expression and promotes clonal expansion. Nat. Commun..

[17] Torralba D., Martín-Cófreces N.B., Sanchez-Madrid F. (2019). Mechanisms of polarized cell-cell communication of T lymphocytes. Immunol. Lett..

[18] Burel J.G., Pomaznoy M., Lindestam Arlehamn C.S., Weiskopf D., da Silva Antunes R., Jung Y., Babor M., Schulten V., Seumois G., Greenbaum J.A. (2019). Circulating T cell-monocyte complexes are markers of immune perturbations. Elife.

[19] Darcis G., Kootstra N.A., Hooibrink B., van Montfort T., Maurer I., Groen K., Jurriaans S., Bakker M., van Lint C., Berkhout B., Pasternak A.O. (2020). CD32(+)CD4(+) T Cells Are Highly Enriched for HIV DNA and Can Support Transcriptional Latency. Cell Rep..

[20] Albanese M., Ruhle A., Mittermaier J., Mejías-Pérez E., Gapp M., Linder A., Schmacke N.A., Hofmann K., Hennrich A.A., Levy D.N. (2022). Rapid, efficient and activation-neutral gene editing of polyclonal primary human resting CD4(+) T cells allows complex functional analyses. Nat. Methods.

[21] Masuda S., Iwasaki S., Tomaru U., Sato J., Kawakami A., Ichijo K., Sogo S., Baba T., Katsumata K., Kasahara M., Ishizu A. (2012). Mechanism of Fcγ receptor-mediated trogocytosis-based false-positive results in flow cytometry. PLoS One.

[22] Stojanovic A., Correia M.P., Cerwenka A. (2018). The NKG2D/NKG2DL Axis in the Crosstalk Between Lymphoid and Myeloid Cells in Health and Disease. Front. Immunol..

[23] Coggeshall K.M. (2002). Regulation of signal transduction by the Fc gamma receptor family members and their involvement in autoimmunity. Curr. Dir. Autoimmun..

[24] Baldauf H.M., Pan X., Erikson E., Schmidt S., Daddacha W., Burggraf M., Schenkova K., Ambiel I., Wabnitz G., Gramberg T. (2012). SAMHD1 restricts HIV-1 infection in resting CD4(+) T cells. Nat. Med..

[25] Roghanian A., Stopforth R.J., Dahal L.N., Cragg M.S. (2018). New revelations from an old receptor: Immunoregulatory functions of the inhibitory Fc gamma receptor, FcγRIIB (CD32B). J. Leukoc. Biol..

[26] Roberts J.T., Patel K.R., Barb A.W. (2020). Site-specific N-glycan Analysis of Antibody-binding Fc γ Receptors from Primary Human Monocytes. Mol. Cell. Proteomics.

[27] White A.L., Beers S.A., Cragg M.S. (2014). FcγRIIB as a key determinant of agonistic antibody efficacy. Curr. Top. Microbiol. Immunol..

[28] Weigert M., Schmidt U., Boothe T., Müller A., Dibrov A., Jain A., Wilhelm B., Schmidt D., Broaddus C., Culley S. (2018). Content-aware image restoration: pushing the limits of fluorescence microscopy. Nat. Methods.

[29] Dance A. (2019). Core Concept: Cells nibble one another via the under-appreciated process of trogocytosis. Proc. Natl. Acad. Sci. USA.

[30] Uribe-Querol E., Rosales C. (2021). The Multiple Roles of Trogocytosis in Immunity, the Nervous System, and Development. BioMed Res. Int..

[31] Daubeuf S., Aucher A., Bordier C., Salles A., Serre L., Gaibelet G., Faye J.C., Favre G., Joly E., Hudrisier D. (2010). Preferential transfer of certain plasma membrane proteins onto T and B cells by trogocytosis. PLoS One.

[32] Hudrisier D., Aucher A., Puaux A.L., Bordier C., Joly E. (2007). Capture of target cell membrane components via trogocytosis is triggered by a selected set of surface molecules on T or B cells. J. Immunol..

[33] Bettadapur A., Miller H.W., Ralston K.S. (2020). Biting Off What Can Be Chewed: Trogocytosis in Health, Infection, and Disease. Infect. Immun..

[34] Li Y., Basar R., Wang G., Liu E., Moyes J.S., Li L., Kerbauy L.N., Uprety N., Fathi M., Rezvan A. (2022). KIR-based inhibitory CARs overcome CAR-NK cell trogocytosis-mediated fratricide and tumor escape. Nat. Med..

[35] Taylor R.P., Lindorfer M.A. (2015). Fcγ-receptor-mediated trogocytosis impacts mAb-based therapies: historical precedence and recent developments. Blood.

[36] Schriek P., Ching A.C., Moily N.S., Moffat J., Beattie L., Steiner T.M., Hosking L.M., Thurman J.M., Holers V.M., Ishido S. (2022). Marginal zone B cells acquire dendritic cell functions by trogocytosis. Science.

[37] Badia R., Ballana E., Castellví M., García-Vidal E., Pujantell M., Clotet B., Prado J.G., Puig J., Martínez M.A., Riveira-Muñoz E., Esté J.A. (2018). CD32 expression is associated to T-cell activation and is not a marker of the HIV-1 reservoir. Nat. Commun..

[38] Pham T., Mero P., Booth J.W. (2011). Dynamics of macrophage trogocytosis of rituximab-coated B cells. PLoS One.

[39] Daubeuf S., Lindorfer M.A., Taylor R.P., Joly E., Hudrisier D. (2010). The direction of plasma membrane exchange between lymphocytes and accessory cells by trogocytosis is influenced by the nature of the accessory cell. J. Immunol..

[40] Iwasaki S., Masuda S., Baba T., Tomaru U., Katsumata K., Kasahara M., Ishizu A. (2011). Plasma-dependent, antibody- and Fcγ receptor-mediated translocation of CD8 molecules from T cells to monocytes. Cytometry A..

[41] Chen H., Maul-Pavicic A., Holzer M., Huber M., Salzer U., Chevalier N., Voll R.E., Hengel H., Kolb P. (2022). Detection and functional resolution of soluble immune complexes by an FcγR reporter cell panel. EMBO Mol. Med..

[42] Strazza M., Azoulay-Alfaguter I., Pedoeem A., Mor A. (2014). Static adhesion assay for the study of integrin activation in T lymphocytes. J. Vis. Exp..

[43] Falkowska E., Le K.M., Ramos A., Doores K.J., Lee J.H., Blattner C., Ramirez A., Derking R., van Gils M.J., Liang C.H. (2014). Broadly neutralizing HIV antibodies define a glycan-dependent epitope on the prefusion conformation of gp41 on cleaved envelope trimers. Immunity.

[44] Huang J., Kang B.H., Pancera M., Lee J.H., Tong T., Feng Y., Imamichi H., Georgiev I.S., Chuang G.Y., Druz A. (2014). Broad and potent HIV-1 neutralization by a human antibody that binds the gp41-gp120 interface. Nature.

[45] Walker L.M., Huber M., Doores K.J., Falkowska E., Pejchal R., Julien J.P., Wang S.K., Ramos A., Chan-Hui P.Y., Moyle M. (2011). Broad neutralization coverage of HIV by multiple highly potent antibodies. Nature.

[46] Baker D., Ali L., Saxena G., Pryce G., Jones M., Schmierer K., Giovannoni G., Gnanapavan S., Munger K.C., Samkoff L. (2020). The Irony of Humanization: Alemtuzumab, the First, But One of the Most Immunogenic, Humanized Monoclonal Antibodies. Front. Immunol..

[47] Wang X., Mathieu M., Brezski R.J. (2018). IgG Fc engineering to modulate antibody effector functions. Protein Cell.

[48] Corrales-Aguilar E., Trilling M., Hunold K., Fiedler M., Le V.T.K., Reinhard H., Ehrhardt K., Mercé-Maldonado E., Aliyev E., Zimmermann A. (2014). Human cytomegalovirus Fcγ binding proteins gp34 and gp68 antagonize Fcγ receptors I, II and III. PLoS Pathog..

[49] Kolb P., Hoffmann K., Sievert A., Reinhard H., Merce-Maldonado E., Le-Trilling V.T.K., Halenius A., Gütle D., Hengel H. (2021). Human cytomegalovirus antagonizes activation of Fcγ receptors by distinct and synergizing modes of IgG manipulation. Elife.

[50] Adams P., Fievez V., Schober R., Amand M., Iserentant G., Rutsaert S., Dessilly G., Vanham G., Hedin F., Cosma A. (2021). CD32(+)CD4(+) memory T cells are enriched for total HIV-1 DNA in tissues from humanized mice. iScience.

[51] Martin G.E., Pace M., Thornhill J.P., Phetsouphanh C., Meyerowitz J., Gossez M., Brown H., Olejniczak N., Lwanga J., Ramjee G. (2018). CD32-Expressing CD4 T Cells Are Phenotypically Diverse and Can Contain Proviral HIV DNA. Front. Immunol..

[52] Lai J., Bernhard O.K., Turville S.G., Harman A.N., Wilkinson J., Cunningham A.L. (2009). Oligomerization of the macrophage mannose receptor enhances gp120-mediated binding of HIV-1. J. Biol. Chem..

[53] Baribaud F., Pöhlmann S., Doms R.W. (2001). The role of DC-SIGN and DC-SIGNR in HIV and SIV attachment, infection, and transmission. Virology.

[54] DeLucia D.C., Rinaldo C.R., Rappocciolo G. (2018). Inefficient HIV-1 trans Infection of CD4(+) T Cells by Macrophages from HIV-1 Nonprogressors Is Associated with Altered Membrane Cholesterol and DC-SIGN. J. Virol..

[55] Fanibunda S.E., Modi D.N., Gokral J.S., Bandivdekar A.H. (2011). HIV gp120 binds to mannose receptor on vaginal epithelial cells and induces production of matrix metalloproteinases. PLoS One.

[56] Geijtenbeek T.B., Kwon D.S., Torensma R., van Vliet S.J., van Duijnhoven G.C., Middel J., Cornelissen I.L., Nottet H.S., KewalRamani V.N., Littman D.R. (2000). DC-SIGN, a dendritic cell-specific HIV-1-binding protein that enhances trans-infection of T cells. Cell.

[57] Bajtay Z., Speth C., Erdei A., Dierich M.P. (2004). Cutting edge: productive HIV-1 infection of dendritic cells via complement receptor type 3 (CR3, CD11b/CD18). J. Immunol..

[58] Day C.J., Hardison R.L., Spillings B.L., Poole J., Jurcisek J.A., Mak J., Jennings M.P., Edwards J.L. (2022). Complement Receptor 3 Mediates HIV-1 Transcytosis across an Intact Cervical Epithelial Cell Barrier: New Insight into HIV Transmission in Women. mBio.

[59] Joly E., Hudrisier D. (2003). What is trogocytosis and what is its purpose?. Nat. Immunol..

[60] Lindorfer M.A., Taylor R.P. (2022). FcγR-Mediated Trogocytosis 2.0: Revisiting History Gives Rise to a Unifying Hypothesis. Antibodies.

[61] Miyake K., Karasuyama H. (2021). The Role of Trogocytosis in the Modulation of Immune Cell Functions. Cells.

[62] Brouwer K.C., Lal R.B., Mirel L.B., Yang C., van Eijk A.M., Ayisi J., Otieno J., Nahlen B.L., Steketee R., Lal A.A., Shi Y.P. (2004). Polymorphism of Fc receptor IIa for IgG in infants is associated with susceptibility to perinatal HIV-1 infection. AIDS.

[63] Forthal D.N., Landucci G., Bream J., Jacobson L.P., Phan T.B., Montoya B. (2007). FcgammaRIIa genotype predicts progression of HIV infection. J. Immunol..

[64] Ananworanich J., Chomont N., Eller L.A., Kroon E., Tovanabutra S., Bose M., Nau M., Fletcher J.L.K., Tipsuk S., Vandergeeten C. (2016). HIV DNA Set Point is Rapidly Established in Acute HIV Infection and Dramatically Reduced by Early ART. EBioMedicine.

[65] Connolly S., Wall K.M., Tang J., Yu T., Kilembe W., Kijak G., Allen S., Hunter E. (2018). Fc-gamma receptor IIA and IIIA variants in two African cohorts: Lack of consistent impact on heterosexual HIV acquisition, viral control, and disease progression. Virology.

[66] Milligan C., Richardson B.A., John-Stewart G., Nduati R., Overbaugh J. (2015). FCGR2A and FCGR3A Genotypes in Human Immunodeficiency Virus Mother-to-Child Transmission. Open Forum Infect. Dis..

[67] Geraghty D.E., Thorball C.W., Fellay J., Thomas R. (2019). Effect of Fc Receptor Genetic Diversity on HIV-1 Disease Pathogenesis. Front. Immunol..

[68] Lamptey H., Bonney E.Y., Adu B., Kyei G.B. (2021). Are Fc Gamma Receptor Polymorphisms Important in HIV-1 Infection Outcomes and Latent Reservoir Size?. Front. Immunol..

[69] Dhummakupt A., Siems L.V., Singh D., Chen Y.H., Anderson T., Collinson-Streng A., Zhang H., Patel P., Agwu A., Persaud D. (2019). The Latent Human Immunodeficiency Virus (HIV) Reservoir Resides Primarily in CD32-CD4+ T Cells in Perinatally HIV-Infected Adolescents With Long-Term Virologic Suppression. J. Infect. Dis..

[70] García M., Navarrete-Muñoz M.A., Ligos J.M., Cabello A., Restrepo C., López-Bernaldo J.C., de la Hera F.J., Barros C., Montoya M., Fernández-Guerrero M. (2018). CD32 Expression is not Associated to HIV-DNA content in CD4 cell subsets of individuals with Different Levels of HIV Control. Sci. Rep..

[71] Bertagnolli L.N., White J.A., Simonetti F.R., Beg S.A., Lai J., Tomescu C., Murray A.J., Antar A.A.R., Zhang H., Margolick J.B. (2018). The role of CD32 during HIV-1 infection. Nature.

[72] Kozak S.L., Heard J.M., Kabat D. (2002). Segregation of CD4 and CXCR4 into distinct lipid microdomains in T lymphocytes suggests a mechanism for membrane destabilization by human immunodeficiency virus. J. Virol..

[73] Cicala C., Martinelli E., McNally J.P., Goode D.J., Gopaul R., Hiatt J., Jelicic K., Kottilil S., Macleod K., O'Shea A. (2009). The integrin alpha4beta7 forms a complex with cell-surface CD4 and defines a T-cell subset that is highly susceptible to infection by HIV-1. Proc. Natl. Acad. Sci. USA.

[74] Lakshmanappa Y.S., Roh J.W., Rane N.N., Dinasarapu A.R., Tran D.D., Velu V., Sheth A.N., Ofotokun I., Amara R.R., Kelley C.F. (2021). Circulating integrin α(4) β(7)(+) CD4 T cells are enriched for proliferative transcriptional programs in HIV infection. FEBS Lett..

[75] Hamieh M., Dobrin A., Cabriolu A., van der Stegen S.J.C., Giavridis T., Mansilla-Soto J., Eyquem J., Zhao Z., Whitlock B.M., Miele M.M. (2019). CAR T cell trogocytosis and cooperative killing regulate tumour antigen escape. Nature.

[76] Greenman R., Pizem Y., Haus-Cohen M., Horev G., Denkberg G., Shen-Orr S., Rubinstein J., Reiter Y. (2021). Phenotypic Models of CAR T-Cell Activation Elucidate the Pivotal Regulatory Role of CAR Downmodulation. Mol. Cancer Therapeut..

[77] Miao L., Zhang Z., Ren Z., Tang F., Li Y. (2021). Obstacles and Coping Strategies of CAR-T Cell Immunotherapy in Solid Tumors. Front. Immunol..

[78] Tekguc M., Wing J.B., Osaki M., Long J., Sakaguchi S. (2021). Treg-expressed CTLA-4 depletes CD80/CD86 by trogocytosis, releasing free PD-L1 on antigen-presenting cells. Proc. Natl. Acad. Sci. USA.

[79] Levy D.N., Aldrovandi G.M., Kutsch O., Shaw G.M. (2004). Dynamics of HIV-1 recombination in its natural target cells. Proc. Natl. Acad. Sci. USA.

[80] Horwitz J.A., Bar-On Y., Lu C.L., Fera D., Lockhart A.A.K., Lorenzi J.C.C., Nogueira L., Golijanin J., Scheid J.F., Seaman M.S. (2017). Non-neutralizing Antibodies Alter the Course of HIV-1 Infection In Vivo. Cell.

[81] Cavrois M., De Noronha C., Greene W.C. (2002). A sensitive and specific enzyme-based assay detecting HIV-1 virion fusion in primary T lymphocytes. Nat Biotechnol.

[82] Campbell E.M., Perez O., Melar M., Hope T.J. (2007). Labeling HIV-1 virions with two fluorescent proteins allows identification of virions that have productively entered the target cell. Virology.

[83] Dillon S.M., Manuzak J.A., Leone A.K., Lee E.J., Rogers L.M., McCarter M.D., Wilson C.C. (2012). HIV-1 infection of human intestinal lamina propria CD4+ T cells in vitro is enhanced by exposure to commensal Escherichia coli. J. Immunol..

[84] Steele A.K., Lee E.J., Manuzak J.A., Dillon S.M., Beckham J.D., McCarter M.D., Santiago M.L., Wilson C.C. (2014). Microbial exposure alters HIV-1-induced mucosal CD4+ T cell death pathways Ex vivo. Retrovirology.

[85] Chase A.J., Wombacher R., Fackler O.T. (2018). Intrinsic properties and plasma membrane trafficking route of Src family kinase SH4 domains sensitive to retargeting by HIV-1 Nef. J. Biol. Chem..

[86] Müller B., Anders M., Reinstein J. (2014). In vitro analysis of human immunodeficiency virus particle dissociation: gag proteolytic processing influences dissociation kinetics. PLoS One.

[87] Geuenich S., Goffinet C., Venzke S., Nolkemper S., Baumann I., Plinkert P., Reichling J., Keppler O.T. (2008). Aqueous extracts from peppermint, sage and lemon balm leaves display potent anti-HIV-1 activity by increasing the virion density. Retrovirology.

[88] Venzke S., Michel N., Allespach I., Fackler O.T., Keppler O.T. (2006). Expression of Nef downregulates CXCR4, the major coreceptor of human immunodeficiency virus, from the surfaces of target cells and thereby enhances resistance to superinfection. J. Virol..

[89] Berg S., Kutra D., Kroeger T., Straehle C.N., Kausler B.X., Haubold C., Schiegg M., Ales J., Beier T., Rudy M. (2019). ilastik: interactive machine learning for (bio)image analysis. Nat. Methods.

