# Supplementary material for: Receptor transfer between immune cells by autoantibody-enhanced, CD32-driven trogocytosis is hijacked by HIV-1 to infect resting CD4 T cells
Source: Cell Rep Med. 2024 Apr 4;5(4):101483. doi: 10.1016/j.xcrm.2024.101483 (PMC11031382; doi:10.1016/j.xcrm.2024.101483)
Supplement: Document S1. Figures S1–S17 and Tables S2 and S3 [file mmc1.pdf]

Cell Reports Medicine, Volume *5*

Supplemental information

Receptor transfer between immune cells by autoantibody-enhanced, CD32-driven trogocytosis is hijacked by HIV-1 to infect resting CD4 T cells

Manuel Albanese, Hong-Ru Chen, Madeleine Gapp, Maximilian Muenchhoff, Hsiu-Hui Yang, David Peterhoff, Katja Hoffmann, Qianhao Xiao, Adrian Ruhle, Ina Ambiel, Stephanie Schneider, Ernesto Mejías-Pérez, Marcel Stern, Paul R. Wratil, Katharina Hofmann, Laura Amann, Linda Jocham, Thimo Fuchs, Alessandro F. Ulivi, Simon Besson-Girard, Simon Weidlich, Jochen Schneider, Christoph D. Spinner, Kathrin Sutter, Ulf Dittmer, Andreas Humpe, Philipp Baumeister, Andreas Wieser, Simon Rothenfusser, Johannes Bogner, Julia Roider, Percy Knolle, Hartmut Hengel, Ralf Wagner, Vibor Laketa, Oliver T. Fackler, and Oliver T. Keppler

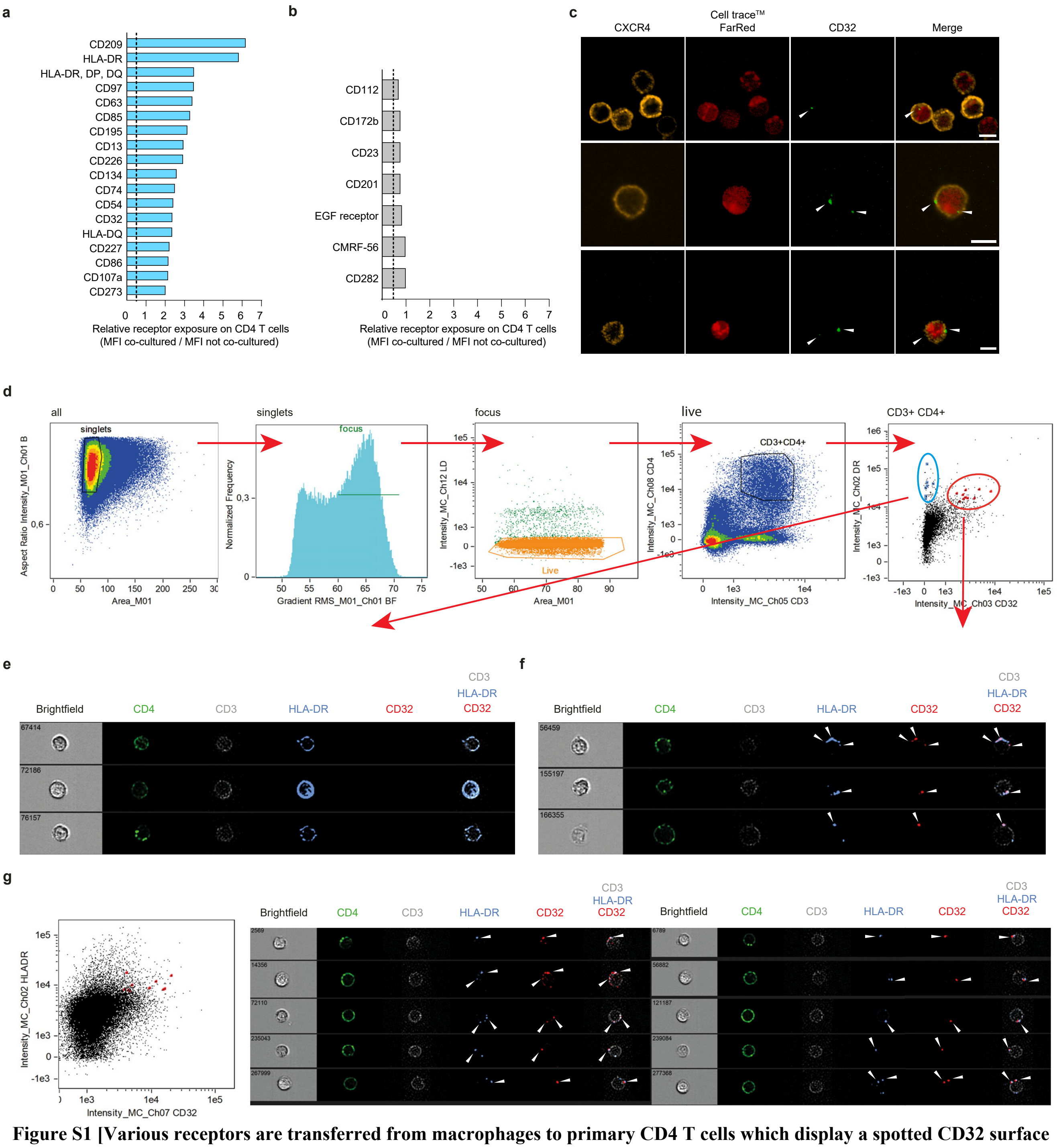

Figure S1 [Various receptors are transferred from macrophages to primary CD4 T cells which display a spotted CD32 surface staining and co-localize with HLA-DR after co-culture with autologous macrophages.], Related to Figure 1. a,b, CD4 T cells were co-cultured with M2 macrophages for 2 days. CD4 T cells were then sorted and stained with antibodies of the BD Lyoplate™ screening panel and analyzed by flow cytometry. Expression on M2 macrophages and CD4 T cells without co-culture was analyzed in parallel. The transfer level of the receptors is shown as the ratio of the mean fluorescence intensity (MFI) of receptors on co-cultured to not co-cultured CD4 T cells (pool of 3 donors). (a), Top hits of the most highly transferred surface receptors (MFI ratio >2). (b), Receptors not detectable on CD4 T cells in the absence of co-culture (negative for the receptor, MFI in the range of the isotype control) and unaltered MFI following co-culture (MFI ratio <1). **c**, Primary CD4 T cells were labeled with CellTrace followed by co-culture with M2 for 2 days. CD4 T cells were separated by sorting and stained for CD32 and CXCR4. Representative confocal microscopy images from one out of two experiments are shown. The arrows indicate the localization of CD32 on CD4 T cells. Scale bar: 5 µm. **d-g**, CD4 T cells freshly isolated from PBMCs (e, f) or tonsils (g) were stained with antibodies against CD4, CD3, HLA-DR and CD32, and then analyzed by AMNIS Imagestream technology. (d), Gating strategy of CD32-pos. and CD32-neg. CD4 T cell populations. The CD32-neg. population (e) and CD32-pos. population (f) are shown with brightfield and fluorescent images for individual HLA-DR-pos. cells. (g) Same procedure and gating for single-cell suspensions obtained from tonsil tissue. Arrow heads indicate the co-localization of CD32 and HLA-DR. Experiments were repeated independently for three donors for PBMC samples and two donors. for tonsil samples.

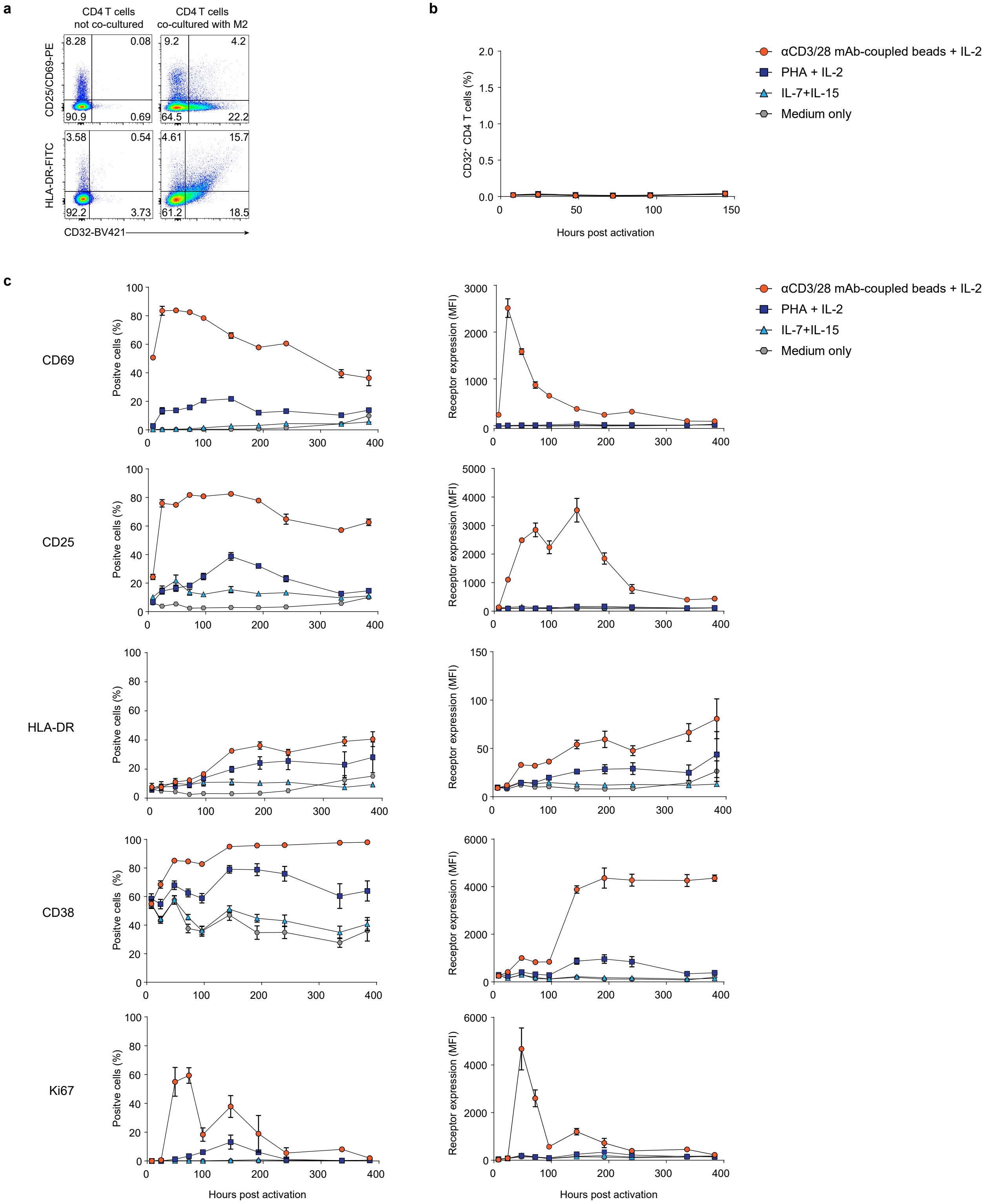

Figure S2 [Different stimuli can induce the expression of various T-cell activation markers, but not of CD32, on CD4 T cells.], Related to Figure 1. a, CD4 T cells from direct co-cultures with M2 from Fig. 1g were co-stained for CD32 and either HLA-DR or the early T cell activation markers CD25/CD69. One representative donor out of three is shown. **b,** isolated CD4 T cells were treated with different T-cell activation stimuli (anti-human CD3/CD28 monoclonal antibody (mAb)-coupled beads + human IL-2; PHA + human IL-2) or remained resting with human IL-7 + human IL-15 or medium only. CD32 levels on CD4 T cells were quantified by flow cytometry. Mean ± s.e.m. is shown (n = 3). **c,** isolated CD4 T cells were treated with activation stimuli as in (b). The expression of activation markers was analyzed by flow cytometry overtime. Shown are either the percentage of positive cells (left column) or the mean fluorescence intensity (MFI) (right column). The mean ± s.e.m. is shown (n = 3).

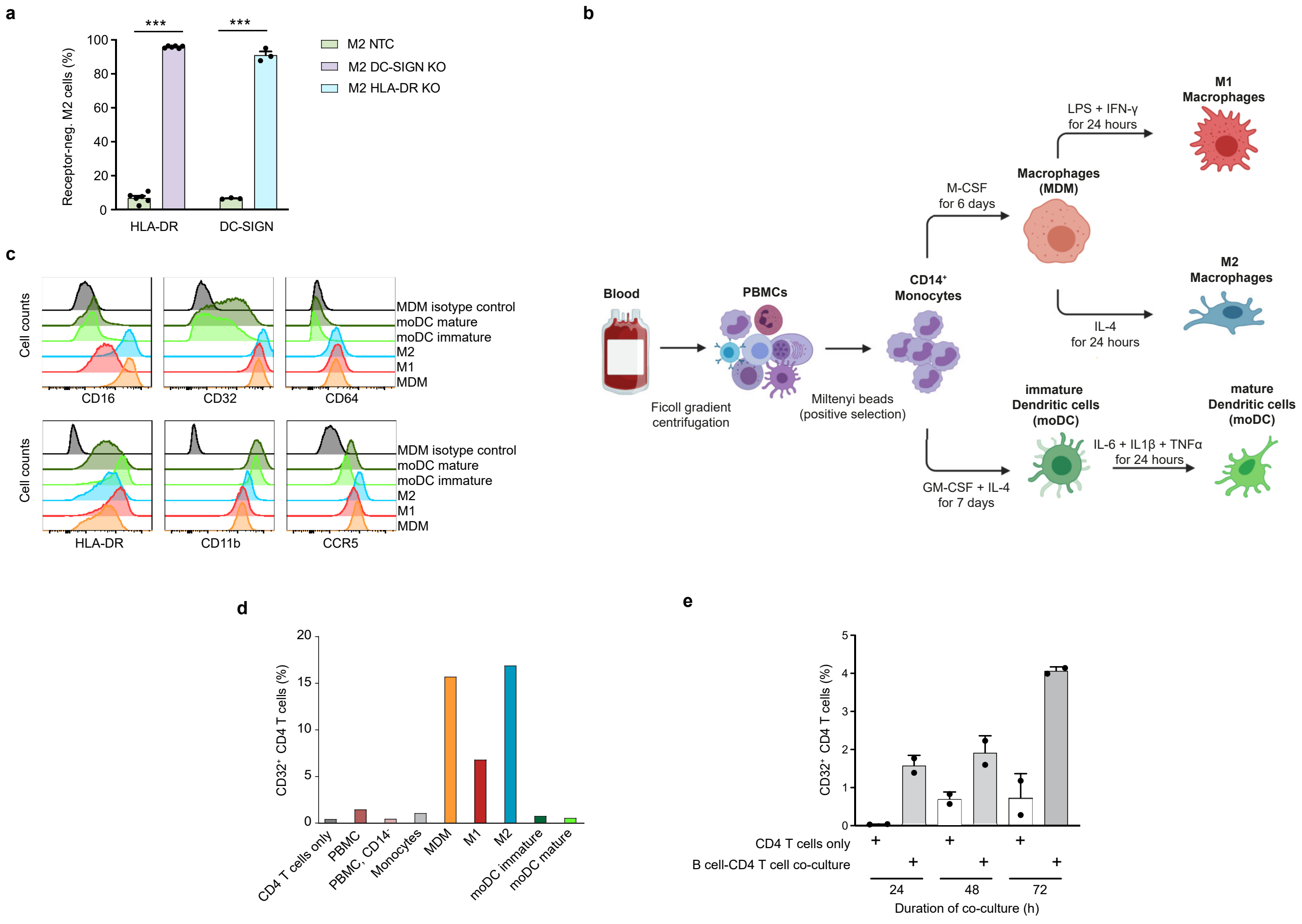

Figure S3 [Exposure of CD32 on CD4 T cells depends on the expression level of CD32 on the co-cultured myeloid cell type.], Related to Figure 1. a, monocytes were isolated from PBMCs and DC-SIGN or HLA-DR were knocked out using specific RNPs. Monocytes were differentiated to M2 macrophages and stained for DC-SIGN or HLA-DR. Non-target control gRNA (NTC) was used as control. Mean ± s.e.m. is shown (n = 3). Statistics indicate significance by two-tailed paired t-test, ***P ≤0.001. **b**, Schematic overview of myeloid lineage differentiation: PBMC isolation from whole blood, followed by Miltenyi bead-based positive selection for CD14-pos. monocytes. Monocytes were terminally differentiated into different myeloid lineages (monocyte-derived macrophages (MDM), M1 macrophages (M1), M2 macrophages (M2), or monocyte-derived dendritic cells (moDC)) by cultivation in the presence of specific cytokines (see Materials and methods). The illustration was created with BioRender.com. **c**, The expression levels of Fcγ receptors (CD16, CD32 and CD64) as well as HLA-DR, CD11b and CCR5 on the indicated myeloid cells were assessed by flow cytometry and are shown as histograms. One representative donor out of three is shown. **d**, Levels of surface-exposed CD32 on CD4 T cells, which had either been co-cultured with the respective autologous cells or cultivated alone. **e**, B cells were isolated from PBMC and co-cultured with autologous CD4 T cells. After 24, 48, and 72 hours of co-culture levels of surface-exposed CD32 was assessed by flow cytometry on co-cultured and non-cocultured CD4 T cells (n = 2).

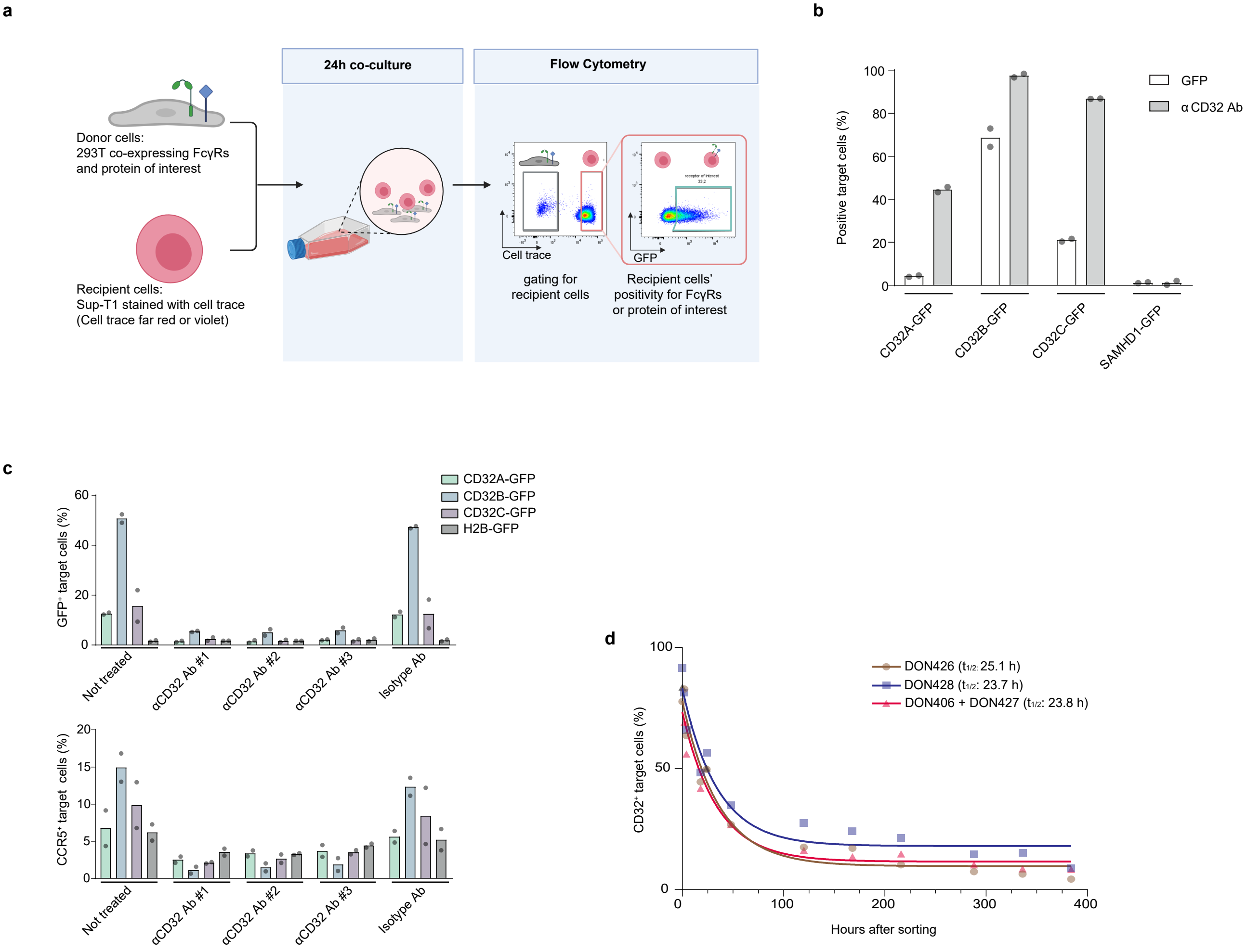

Figure S4 [Cell line-based donor-target model to study CD32-dependent trogocytosis.], Related to Figure 2. a, Schematic figure of the experimental setup: 293T cells were transiently transfected with plasmids encoding FcγRs and proteins of interest are subsequently co-cultured with SupT1 T cells (CellTrace+) as targets. The illustration was created with BioRender.com. **b**, Analysis of the percentage of CD32-neg. and GFP-pos. target T cells as in Fig. 2a. Percentages determined by flow cytometry. Mean is shown (n=2). **c**, 293T cells were transfected as described in Fig. 2c. Before co-cultured with SupT1 T cells, transfected 293T cells were either not treated or pre-treated with one of the three indicated monoclonal anti-CD32 Abs or an isotype control Ab for 30 min. Readouts were acquired as described in Fig. 2c. αCD32 Ab#1 (clone FUN2), αCD32 Ab#2 (clone FLI8.26), αCD32 Ab#3 (clone IV.3). Shown is the mean (n = 2). **d**, After co-culture for two days with autologous M2 cells from the indicated blood donors, CD4 T cells were stained with an anti-CD32 antibody and CellTrace- / CD32-double-pos. cells were sorted. These isolated CD32-pos. CD4 T cells were kept in culture for 16 days. CD32 surface levels were measured at 0, 2, 4, 18, 24, 48, 120, 168, 216, 288, 336, and 384 hours after sorting by flow cytometry.

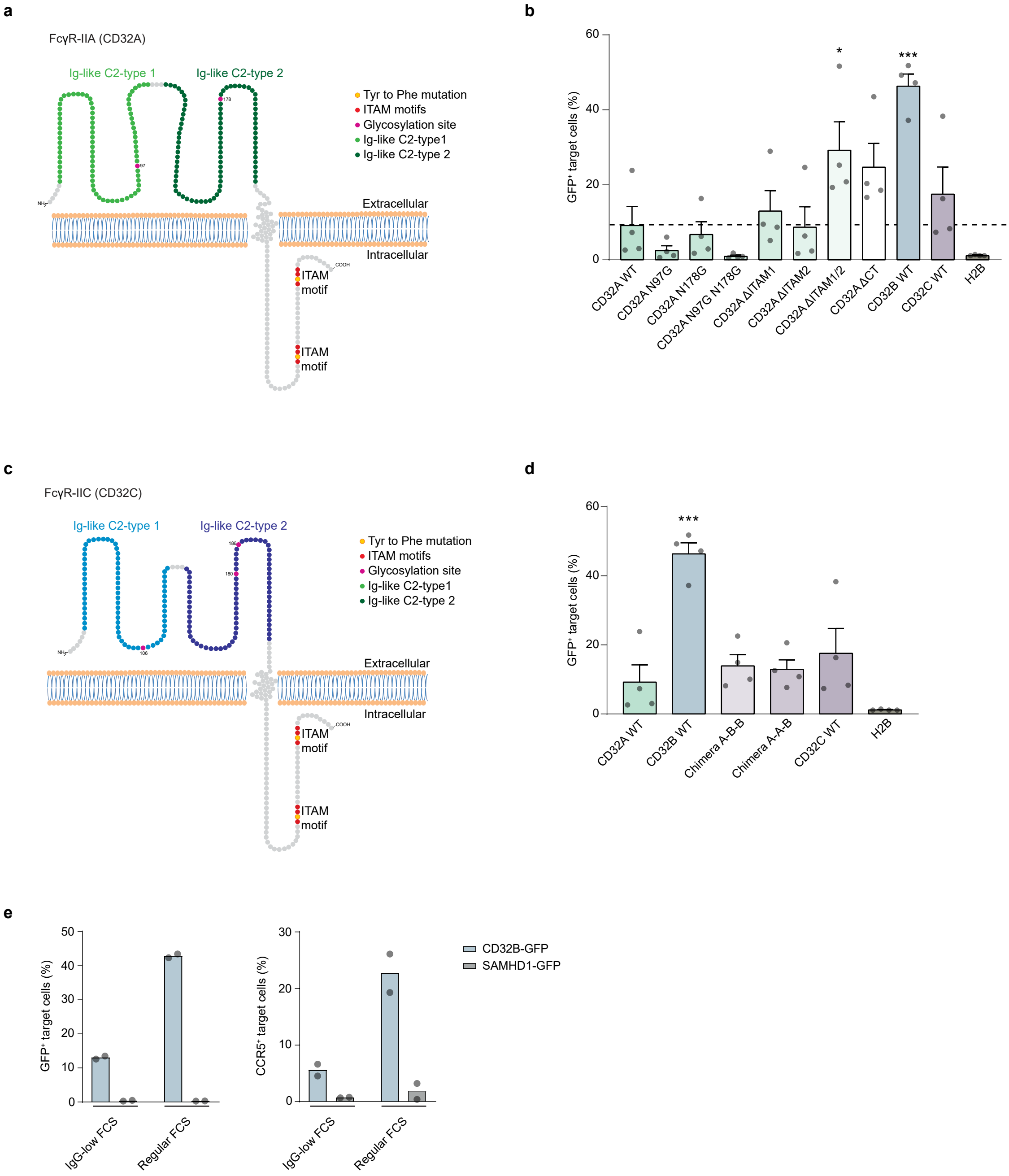

Figure S5 [The impact of activation motifs and *N*-linked glycosylation sites of CD32A and CD32C as well as IgG levels in media on trogocytosis.], Related to Figure 2. a, c, Schematic figure of CD32A (a) and CD32C (c). The ITAM motifs and *N*-linked glycosylation sites are indicated. **b, d**, 293T cells were transfected with plasmids encoding the indicated mutants of CD32A (b) or CD32C WT, or the indicated domain chimeras of CD32A and CD32B (d). The transfer of mutant receptors to target cells was assessed as in Fig. 2f. Chimera A-B-B consists of the extracellular domain of CD32A, transmembrane domain of CD32B, and cytosolic region of CD32B. Chimera A-A-B consists of the extracellular and transmembrane domains of CD32A and intracellular region of CD32B. Mean ± s.e.m. is shown (n = 4). Asterisks indicate statistical significance by one-way ANOVA. P values were corrected for multiple comparison (Dunnett). *P ≤0.05; ***P ≤0.001. **e**, 293T cells and SupT1 cells were cultured in media containing 10% FBS with either ultra-low IgG or regular IgG levels for at least one week. Subsequently, 293T cells were transfected with expression plasmids encoding either CD32B-GFP or SAMHD1-GFP, together with a plasmid encoding CCR5, followed by co-culture with SupT1 cells as described in Fig. S4a. Medium containing either ultra-low IgG or regular IgG FBS (each 10%) were used also during co-culture. Shown is the mean of duplicates.

a

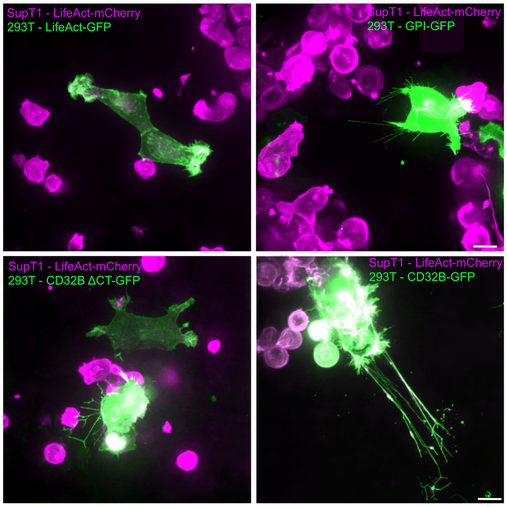

b

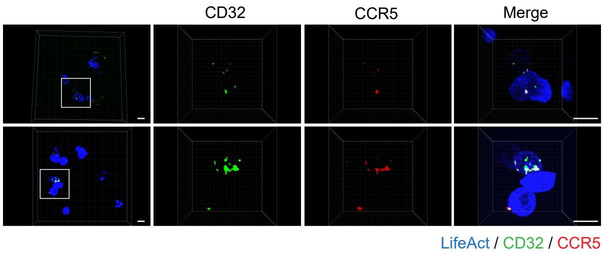

Figure S6 [Short and long-range contacts of 293T cells and SupT1 cells result in the deposition of CD32B-GFP punctae at the surface of SupT1 cells.], Related to Figure 2. a, 293T cells were transfected with pLifeAct-GFP (top left panel), pGPI (anchored)-GFP (top right panel), pCD32BΔCT-GFP (lower left panel) and pCD32B-GFP (lower right, green), mixed with LifeAct-mCherry-expressing SupT1 cells (magenta) and imaged using spinning disc microscopy as described in the STAR METHODS. Note the very long protrusions in the cells co-transfected with pCD32B-GFP which are absent or reduced compared to cells expressing other constructs. Scale bar: 10 µm. **b**, SupT1 cells were transduced with LifeAct-mCherry, and 293T cells were transfected with plasmids encoding CD32B-GFP and CCR5. After pre-treatment with mAb PGT151 and co-culture as described in Fig. S5a, cells were stained for CCR5, fixed and Z-stack images were taken using a spinning disc confocal microscope. Two examples out of five are shown. Scale bar: 10 µm.

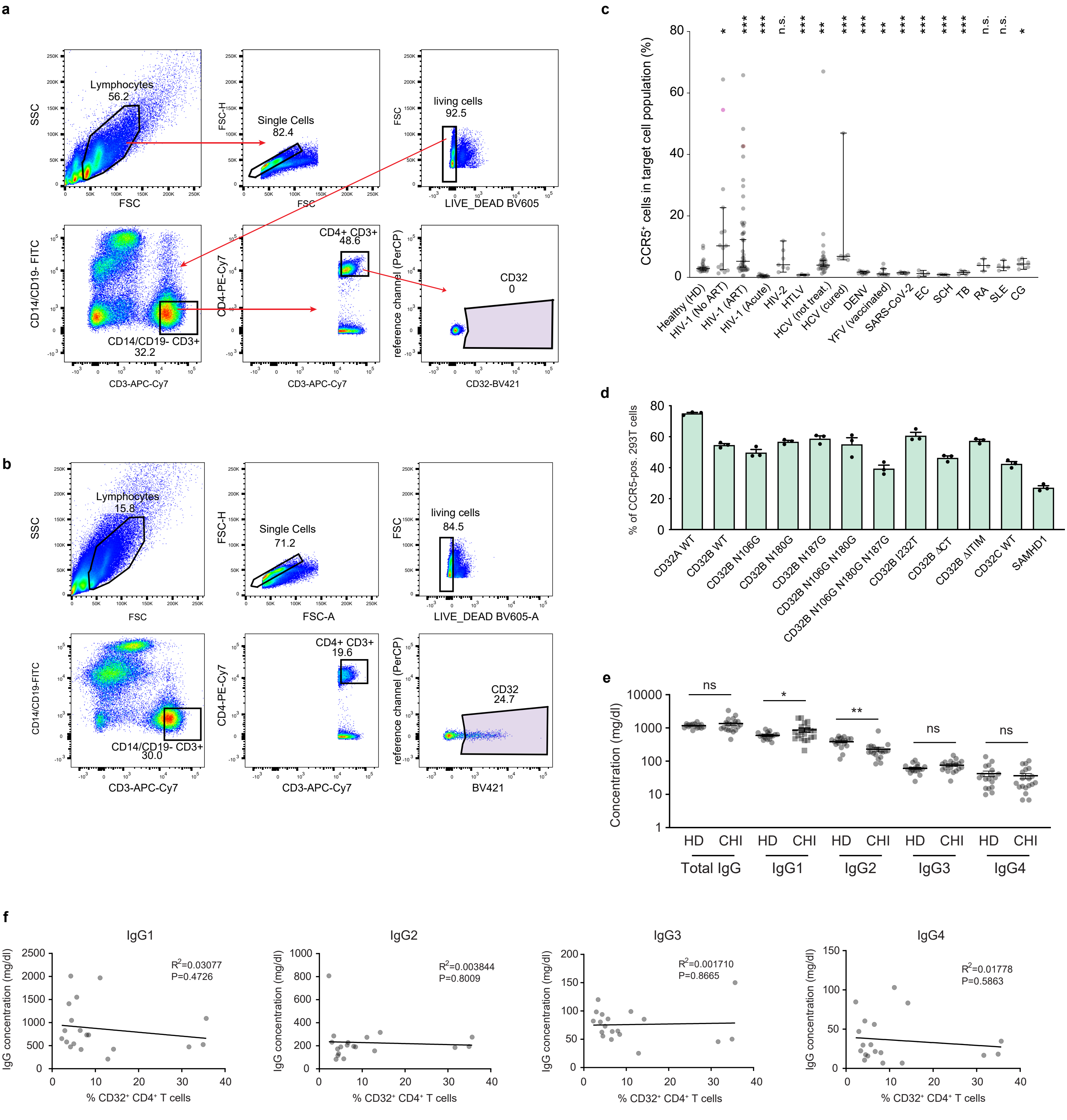

Figure S7 [Gating strategy for identification of CD32-pos. CD4 T cells in PBMC and characteristics of trogocytosis-enhancing sera.], Related to Figure 3. a, b, Lymphocytes were gated using the forward and side scatter area (FSC-A versus SSC-A); doublets were removed by FSC-A to FSC-height (FSC-H). To exclude monocytes and B cells, the CD14-neg. / CD19-neg. / CD3-pos. population was gated, followed by gating for CD4-pos. / CD3-pos. cells. To quantify the fraction of CD32-pos. CD4 T cells, a Fluorescence Minus One (FMO) control was used to set the gate. Representative examples of flow cytometry dot plots of one HD (a) and one HIV-1 patient (CHI) (b). **c, d**, Levels of CCR5 on co-cultured CD4 T cells (c) and on transfected 293T donor cells (d) from the experiment described in the legend of Fig. 3b. **e,** Concentrations of total IgG or the indicated IgG sublasses are shown for serum samples from HD or CHI shown in c. **f,** Correlative analyses of IgG subclass concentrations in serum and the percentage of CD32-pos. CD4 T cells following trogocytotic transfer from donor cells in the presence of the indicated CHI patient samples. Asterisks indicate statistical significance by Mann-Whitney test.*P ≤0.05; **P ≤0.01; ***P ≤0.001. n.s.: not significant.

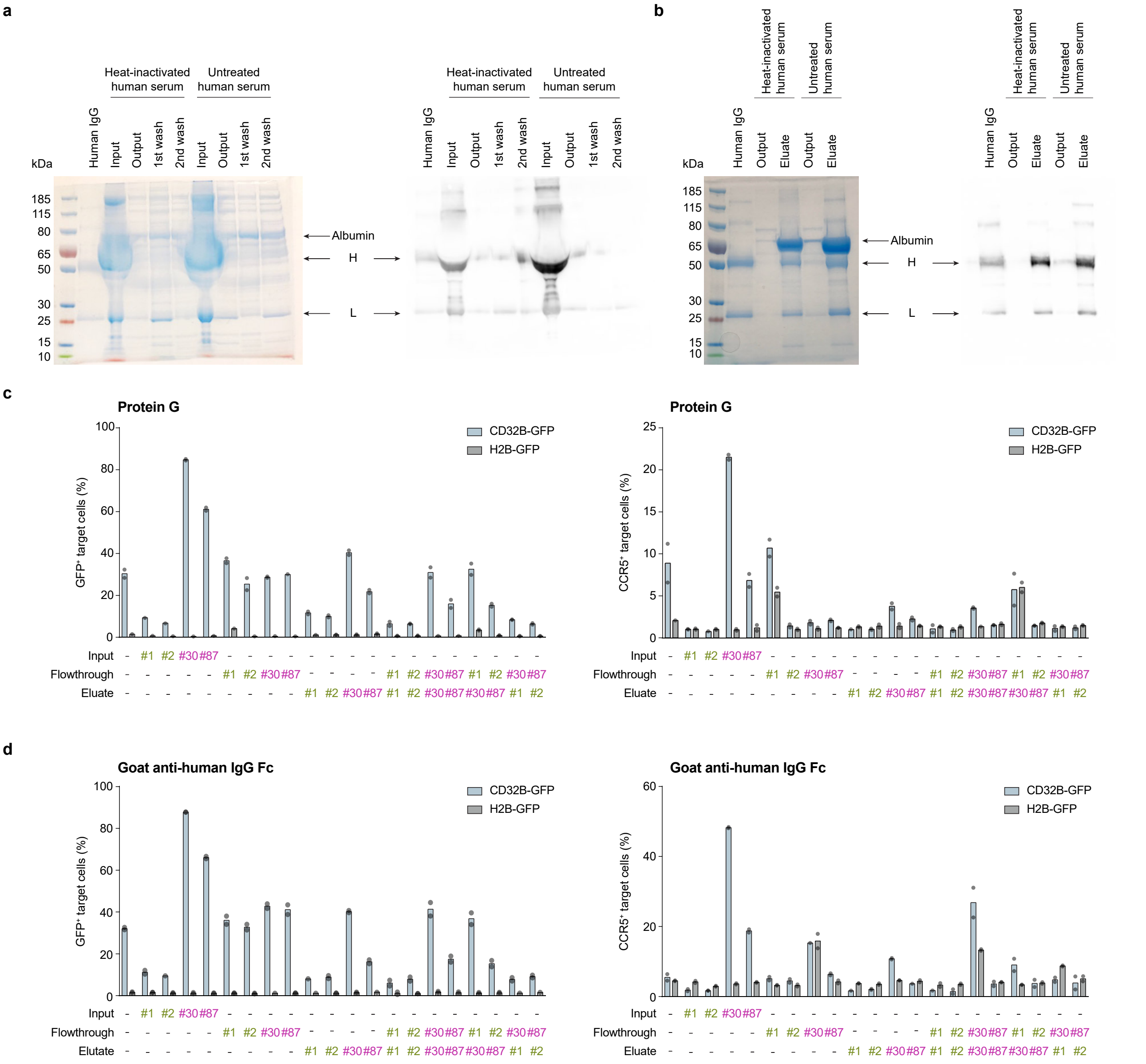

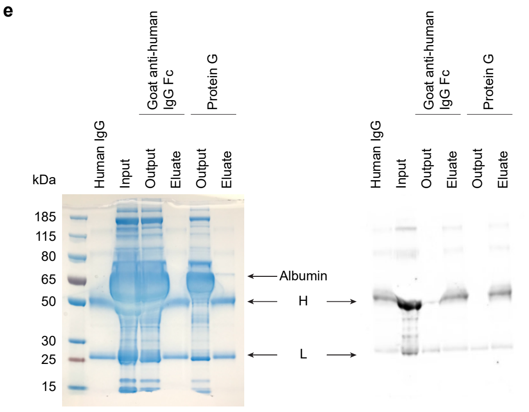

Figure S8 [IgG plays a critical role in trogocytosis-boosting patient sera.], Related to Figure 3. a, Albumin and IgG depletion spin columns were applied to heat-inactivated and untreated human serum from a healthy donor (HD). The input, output and flow-through were collected and analyzed with denaturing SDS-PAGE (left panel) and Western blotting against human IgG (right panel). The same method was applied to serum samples from Fig. 3c. H: heavy chain of IgG. L: light chain of IgG. **b,** Elution of albumin and IgGs binding from the depletion columns in (a). The output of (a) and the eluate were characterized with denaturing SDS-PAGE (left panel) and Western blotting against human IgG (right panel). Polyclonal human IgG was used as a control. **c, d,** Protein G (c) and goat anti-human IgG Fc (d) spin columns were applied to selected serum samples from two HD (green) and two patients with chronic HIV-1 infection (magenta). Samples from CHI donors with top trogocytosis levels in Fig. 3b were selected. The unprocessed sera (input), IgG-depleted sera (flow-through) and human IgG (eluate) were used as described in Fig. 3c. The transfer of CD32B and CCR5 was determined by flow cytometry. Mean of two technical replicates is shown. **e,** Input, output, and eluate of (c) and (d) were analyzed with denaturing SDS-PAGE (left panel) and Western blotting against human IgG (right panel). One representative sample from a healthy donor is shown.

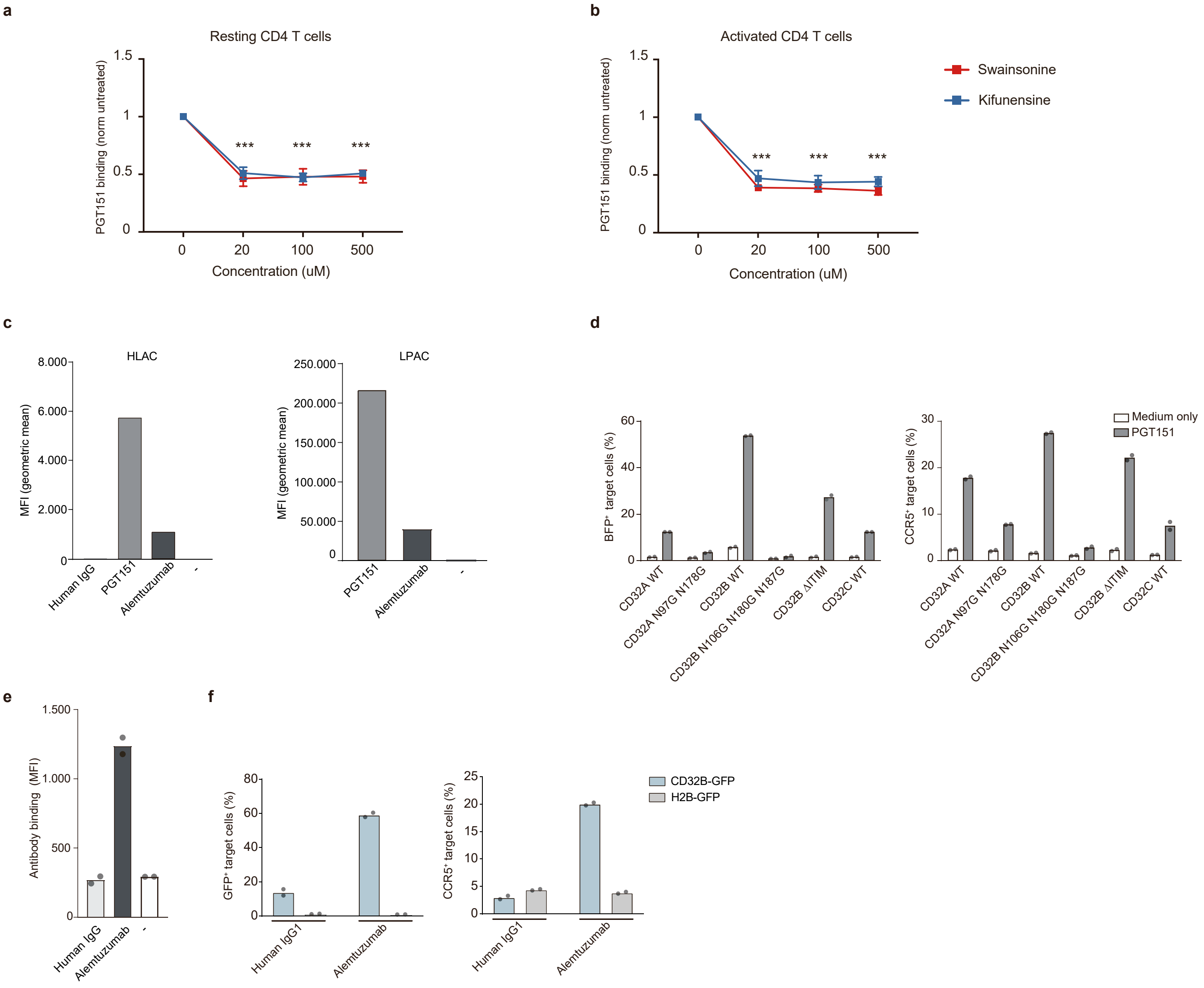

Figure S9 [Reduced binding of bNAb PGT151 to primary CD4 T cells after treatment with α-mannosidase inhibitors, and antibodies that enhance trogocytosis also bind to the surface of CD4 T cells], Related to Figure 3. a,b, Resting (a) or activated (b) CD4 T cells were analysed for their ability to bind mAb PGT151 by flow cyometry after two day-treatment with the indicated concentrations of the α-mannosidase inhibitor II Swainsonine or the α-mannosidase inhibitor I Kifunensine. The MFI values of untreated cells were set to 1. ***: $p<0.001$, Students' t-test (2 tailed). **c,** CD4 T cells were isolated from frozen vials of human lymphoid aggregate cultures from tonsil (HLAC) (left panel, 1 donor) and lamina propria aggregate cultures from the small intestine (LPAC) (right panel, 2 donors). After staining with the indicated primary antibodies and anti-human IgG secondary antibodies, binding levels were assessed by flow cytometry. **d**, 293T cells were transfected with expression plasmids encoding BFP fusion proteins of CD32A and CD32B wildtype (WT) or mutants together with a CCR5 expression plasmid and co-cultured with SupT1 cells as described in Fig. S4a, with or without addition of bNAb PGT151. The transfer of BFP fusion proteins of CD32A/B/C WT, their N-glycosylation sites mutants, CD32B ΔITIM or CCR5-GFP was assessed by flow cytometry. The mean of two technical replicates is shown. **e**, The binding level of anti-CD52 recombinant mAb Alemtuzumab to SupT1 cells was evaluated as described in Fig. 3f. Polyclonal human IgG served as a reference. The mean of two technical replicates is shown. **f**, 293T cells were transfected and pre-treated either with human IgG1 isotype control Abs or mAb Alemtuzumab as described in Fig. 3f. The transfer of CD32B-GFP and CCR5 to SupT1 T cells was assessed by flow cytometry. The mean of two technical replicates is shown.

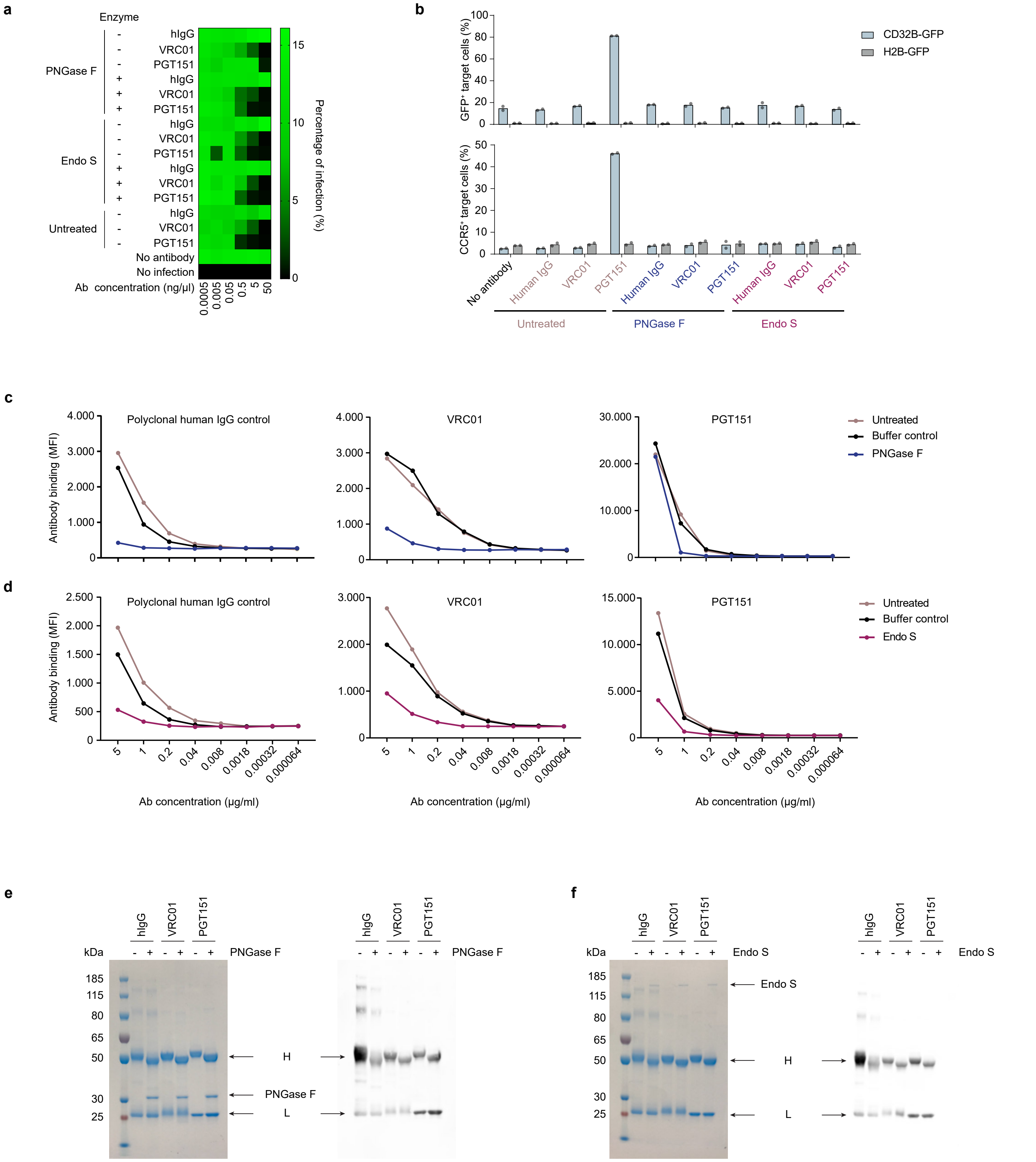

Figure S10 [The *N*-linked glycosylation of bNAb PGT151 is important for binding to CD32B and for boosting trogocytosis, but not for neutralizing HIV-1.], Related to Figure 3. a, Polyclonal IgG, bNAbs PGT151 (boosting) and VRC01 (non-boosting) were either treated with PNGase F, Endo S or left untreated. Subsequently, antibodies were incubated with primary CD4 T cells at the indicated concentrations. Next, cells were challenged with HIV-1 GFP and cultivated for 3 days. The percentage of infection was determined by flow cytometry and is shown as a heatmap. **b,** Polyclonal IgG, PGT151 and VRC01 were treated as in (a), and then applied to the 293T-SupT1 co-culture system. The transfer of CD32B-GFP and CCR5 was assessed by flow cytometry. The mean of two technical replicates is shown. **c, d,** Polyclonal human IgG, mAbs PGT151 and VRC01 were treated with PNGase F (c) or Endo S (d) and then incubated with detached CD32B-BFP-expressing 293T cells at the indicated concentrations. After staining with anti-human IgG antibodies, the binding levels of these antibodies were quantified by flow cytometry as the geometric mean of the MFI. **e, f,** Polyclonal IgG, mAbs PGT151 and VRC01 were treated with PNGase F (e) or Endo S (f). Deglycosylation was confirmed by shifted protein bands on denaturing SDS-PAGE (left panel) and Western blotting with anti-human IgG Fc antibodies (right panel). H: heavy chain. L: light chain.

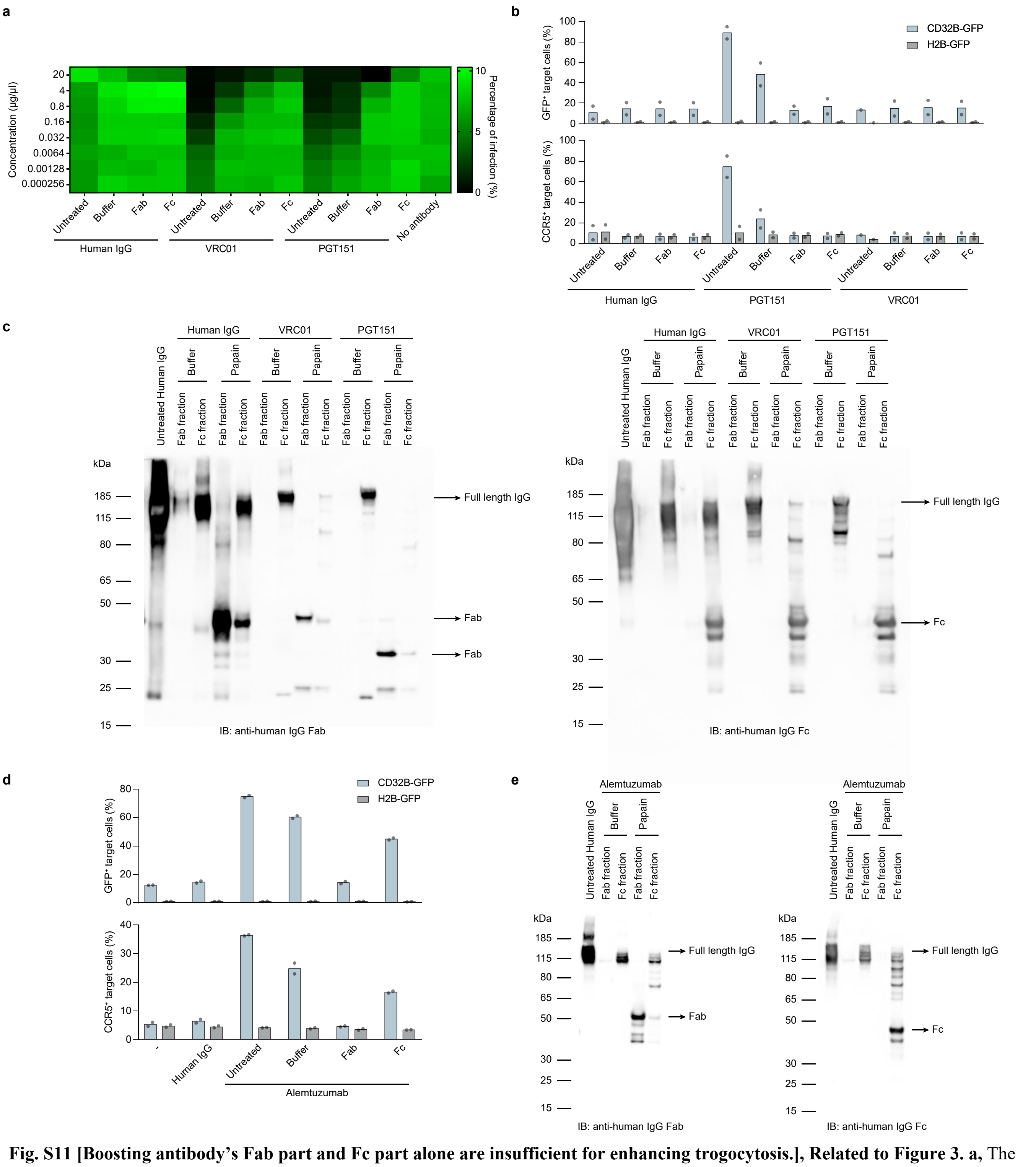

Fig. S11 [Boosting antibody's Fab part and Fc part alone are insufficient for enhancing trogocytosis.], Related to Figure 3. a, The Fab and Fc parts of polyclonal human IgG, bNAbs PGT151 and VRC01 were separated by immobilized papain. Antibody's Fab and Fc fractions were titrated and incubated with CD4 T cells for 30 min prior to challenge with HIV-1 GFP. Untreated and digestion buffer-treated antibodies were included as controls. The percentage of HIV-1 GFP infection was assessed by flow cytometry and is shown as a heatmap. **b,** Fab and Fc parts of polyclonal human IgG, mAbs PGT151 and VRC01 were separated as described in (a) and assessed for trogocytosis enhancement in the 293T-SupT1 co-culture system. The transfer of CD32 and CCR5 was quantified by flow cytometry. Shown is the mean (n=2). **c,** The purity of Fab and Fc parts in (a) and (b) was assessed with native SDS-PAGE and Western blotting using secondary antibodies specific for human IgG Fab (left panel) or human IgG Fc (right panel), respectively. Untreated polyclonal human IgG was used as a control. **d,** mAb Alemtuzumab was treated with immobilized papain as in (a). The resultant Fab and Fc fractions were applied in the 293T-SupT1 co-culture system. Untreated polyclonal human IgG, untreated and buffer-treated Alemtuzumab were used as controls. The transfer of CD32 and CCR5 was quantified by flow cytometry. Mean of two technical replicates is shown. **e,** The purity of Fab and Fc fractions of Alemtuzumab was assessed by native SDS-PAGE and Western blotting using antibodies specific for Fab (left panel) or Fc (right panel) parts of human IgGs.

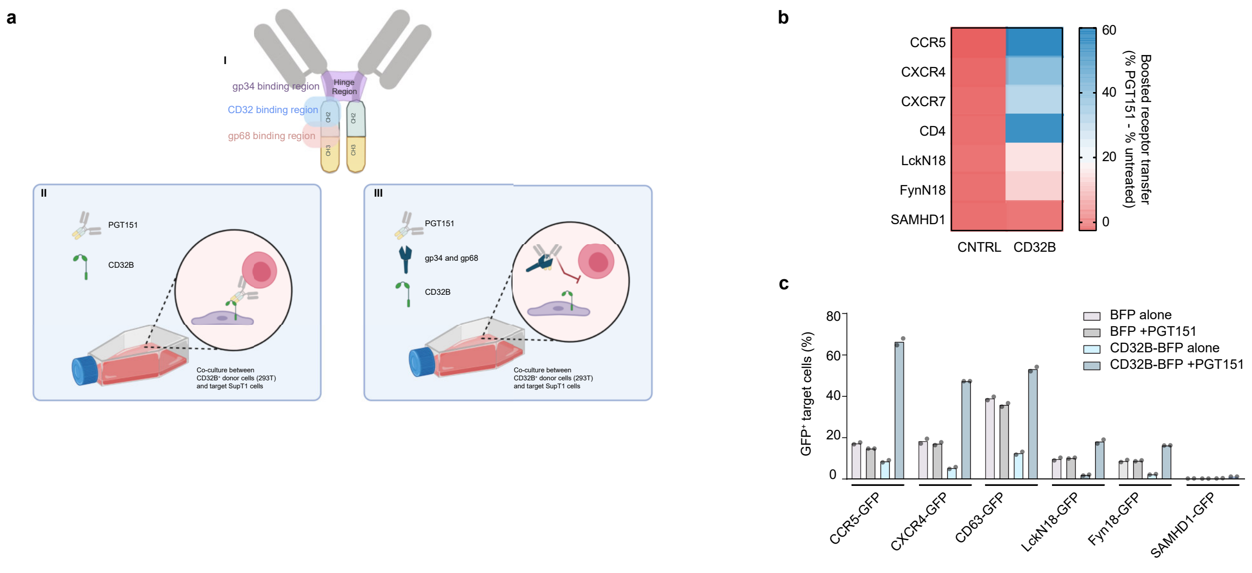

Figure S12 [CMV gp34 and gp68 block the binding of bNAb PGT151 to CD32B.], Related to Figure 3. a, Schematic overview of different binding sites of cytomegalovirus (CMV) glycoproteins 34 and 68 (gp34 and gp68) and CD32 to the Fc part of antibodies (I). (II) In co-culture system described in Fig. S4a, we proposed that the Fc region of PGT151 binds to 293T donor cells through CD32B and the Fab part binds to SupT1 target cells. (III) hCMV glycoproteins bind to the Fc region of bNAb PGT151 competing with and inhibiting CD32B binding. The illustration was created with BioRender.com. **b**, 293T cells were co-transfected with plasmids encoding either CD32B-BFP or BFP alone (CNTRL), together with a plasmid encoding either one of the indicated chemokine receptors, CD4, first 18 amino acid of the *N*-terminal part of Src kinase receptors, or SAMHD1. After two days, cells were pre-treated with PGT151 for 30 min before co-culture with SupT1 T cells. One day later, receptor positivity of target T cells was determined by flow cytometry. Means of two experiments are shown as a heatmap. **c**, 293T cells were transfected with expression plasmids encoding either BFP or CD32B-BFP, together with a plasmid encoding the indicated GFP fusion proteins. These cells were subsequently co-cultured with SupT1 cells in the presence or absence of bNAb PGT151. The transfer of GFP-conjugated proteins was assessed by flow cytometry and the mean of two technical replicates is shown.

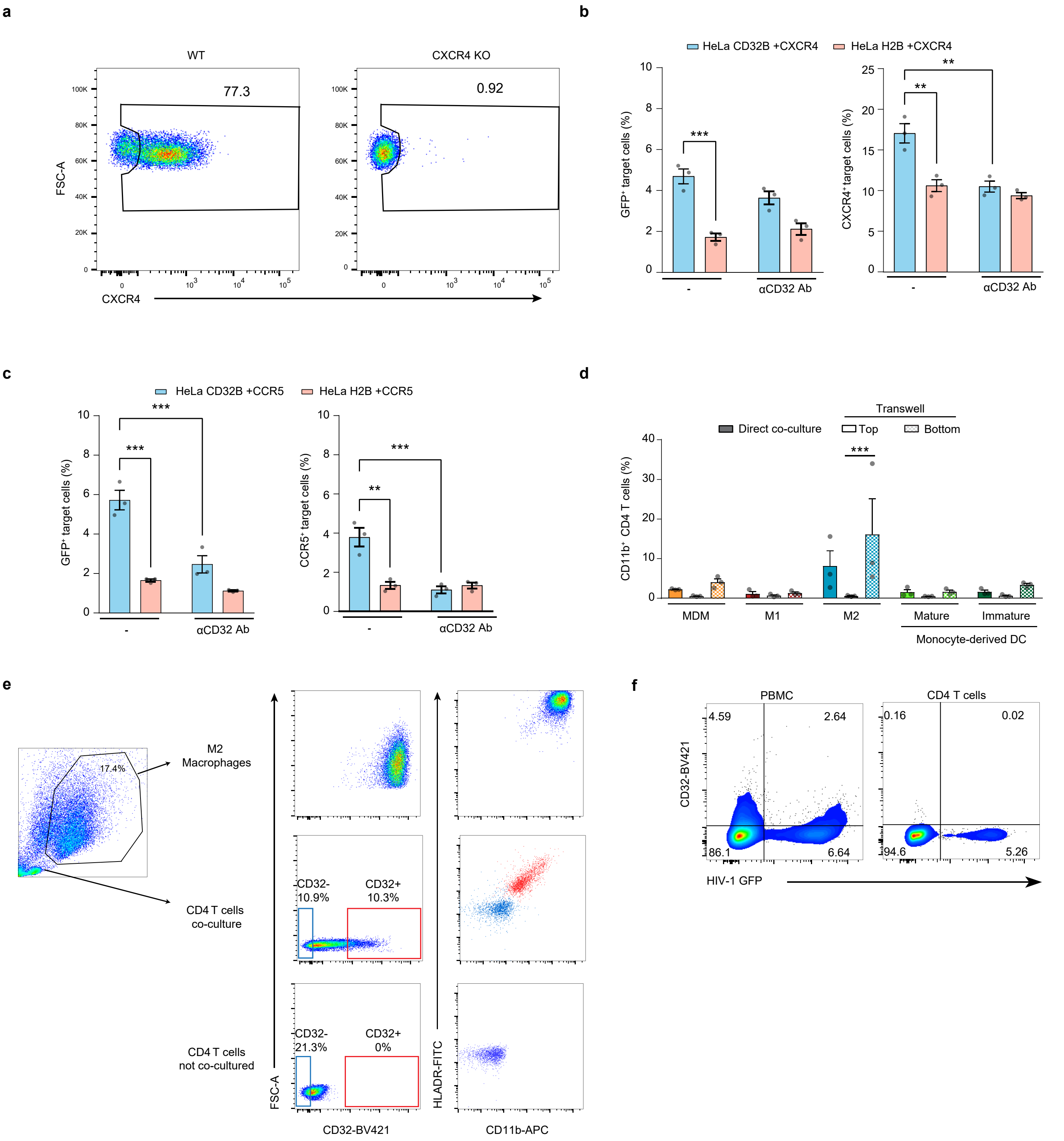

Figure S13 [Trogocytotic co-transfer of CXCR4, CCR5 or CD11b with CD32B.], Related to Figure 4. a, KO confirmation: CXCR4 levels on WT (left panel) and CXCR4 KO (right panel) CD4 T cells were quantified with flow cytometry 7 days after RNP nucleofection. Flow cytometry dot plots of one donor out of 3 are shown. **b,** CXCR4 KO CD4 T cells were stained with CellTrace and then co-cultured for 24 hours with HeLa cells expressing either CD32B-GFP or H2B-GFP together with CXCR4. The transfer of GFP and CXCR4 was quantified by flow cytometry. The mean ± s.e.m. is shown (n = 3). Asterisks indicate statistical significance by two-way ANOVA. *P* values were corrected for multiple comparison (Tukey). **c,** Freshly isolated CD4 T cells were stained with CellTrace and co-cultured for 24 hours with HeLa cells expressing either CD32B-GFP or H2B-GFP together with CCR5. The transfer of GFP and CCR5 was quantified by flow cytometry. Mean ± s.e.m. is shown (n = 3). Asterisks indicate statistical significance by two-way ANOVA. *P* values were corrected for multiple comparison (Tukey). $**P \leq 0.01$; $***P \leq 0.001$. **d,** The indicated myeloid cells were co-cultured with autologous CD4 T cells for 2 days either in direct cell contact or in a transwell setup with CD4 T cells on top, as described in Fig. 1d. Subsequently, CD4 T cells were analyzed for CD11b expression. CD4 T cells migrating to the bottom of the transwell and which therefore also had direct cell contact with differentiated myeloid cells were also collected and analyzed. Mean ± s.e.m. of three independent donors is shown. Asterisks indicate statistical significance by one-way ANOVA; *P* values were corrected for multiple comparison (Tukey). $***P \leq 0.001$. **e,** Characterization of CD11b and HLA-DR expression on the top 10% CD32B-positive (red) or negative (blue) CD4 T cells after co-cultured with M2. **f,** Freshly isolated PBMCs and CD4 T cells highly purified by negative selection, were infected with X4 HIV-1 GFP and 3 days later stained for CD32 surface expression and analyzed by flow cytometry. One representative out of three donor is shown.

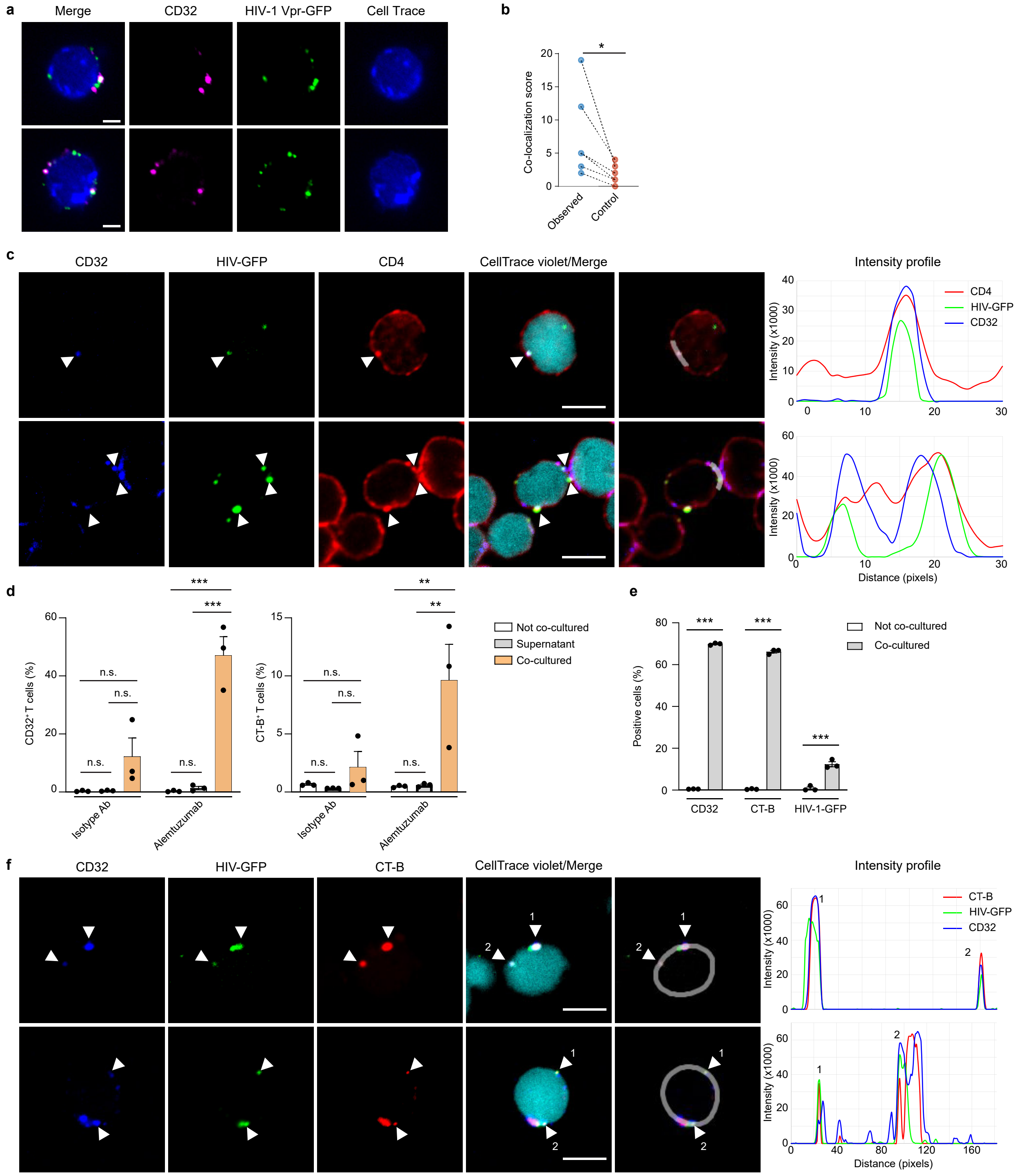

Figure S14 [The HIV-1 susceptibility of CD4 T cells is not increased by de novo expression of CD32.], Related to Figure 4. a, b, HIV-1 binding assay. M2-co-cultured primary CD4 T cells were sorted and challenged with HIV-1 Vpr-GFP particles. Subsequently, cells were stained for CD32. GFP and CD32 positivity of target CD4 T cells were determined by spinning disc confocal microscopy (a). Scale bar: 2 µm. **b,** Co-localization between HIV-1 Vpr-GFP and CD32 in (a) was quantified. The control group was obtained by shuffling z-planes within each stack, separately per each channel, before quantifying co-localization levels. Statistics indicate significance by two-tailed paired t-test. **c,** Two additional examples showing the co-localization of CD32, HIV Vpr-GFP and clustered CD4 as in Fig. 5c. The white arrow heads indicate the co-localization sites. The intensity profiles were analyzed along selected regions on cell surface with ImageJ. Scale bar: 5 µm. **d,** M2 macrophages were stained with AF647-conjugated CT-B prior to co-culture. The supernatant was collected after staining as a control. CellTrace⁺ autologous CD4 T cells were cultivated either alone, or incubated with either supernatant or CT-B-labeled M2 for 2 days, in the presence of either isotype Ab or mAb Alemtuzumab. The transfer of CD32 (left panel) and CT-B (right panel) was assessed by flow cytometry. Mean ± s.e.m. is shown (n=3). Asterisks indicate statistical significance by two-way ANOVA. P values were corrected for multiple comparison (Tukey). **e,** As described in (d), followed by co-cultured with CT-B-labeled M2, CD4 T cells were incubated with HIV-1 Vpr-GFP as described in (a). The transfer of CD32 and CT-B, and the binding of HIV-1 Vpr-GFP were assessed by flow cytometry. Mean ± s.e.m. is shown (n=3). Asterisks indicate statistical significance by two-way ANOVA. P values were corrected for multiple comparison (Šídák). **f,** more images of the experiment described in Fig. 4f and intensity profiles of these images quantified along the surface of the whole cell with ImageJ. The white arrow heads indicate the co-localization sites. Scale bar=5 µm. *P ≤0.05; **P ≤0.01; ***P ≤0.001; n.s.: not significant.

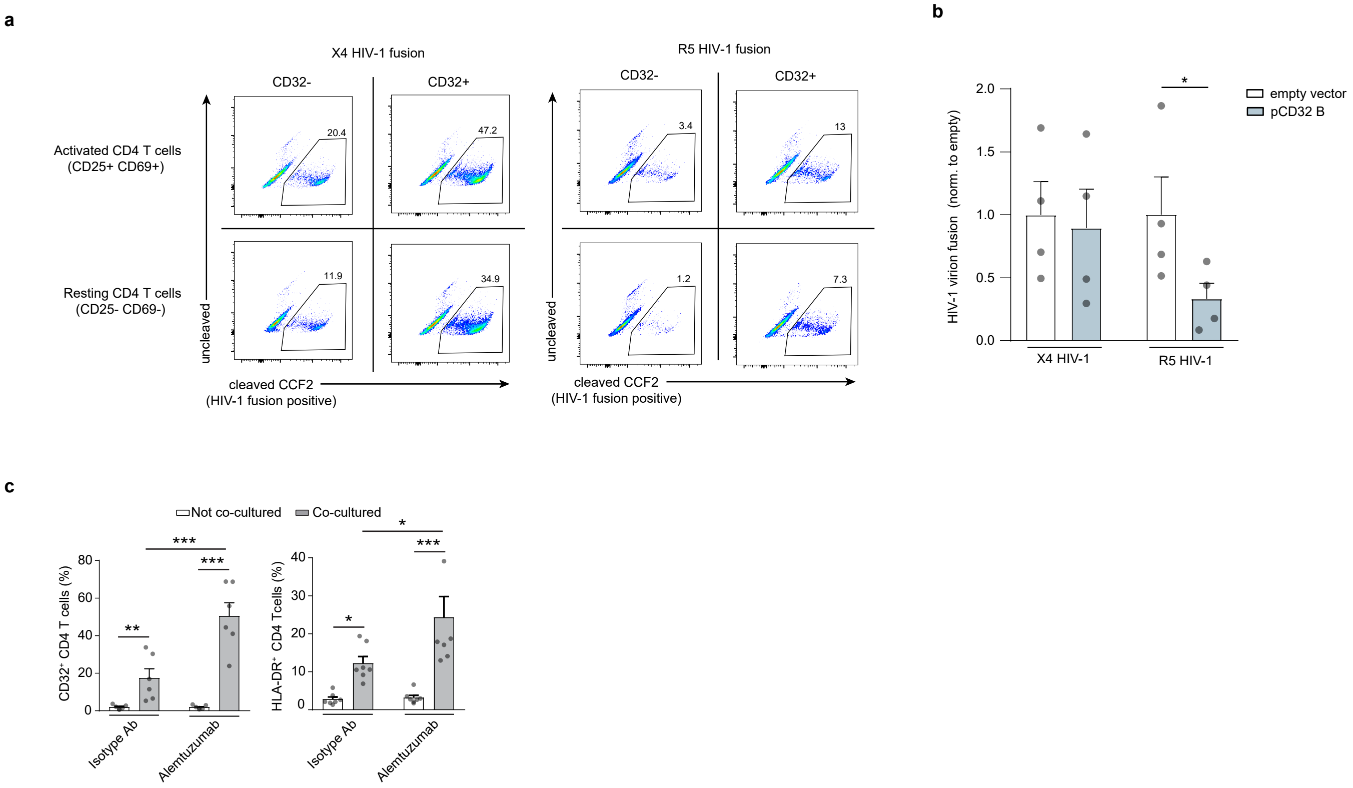

Figure S15 [Following M2 co-culture, CD32^{+} CD4 T cells display an enhanced capacity for HIV-1 fusion.], Related to Figure 4. **a,** Flow cytometry dot plots of HIV-1 fusion in four different CD4 T cell populations, sorted based on CD32 expression and expression of the early activation markers CD69 and CD25. Both X4 HIV-1 (left panel) and R5 HIV-1 (right panel) are shown. One representative donor out of two is shown. **b,** The ability of CD4 T cells to support HIV-1 fusion is not increased by exogenous overexpression of CD32. CD32B was over-expressed in primary CD4 T cells by nucleofection using a CD32B-encoding plasmid. HIV-1 fusion assays were performed with either exogenously CD32-expressing or empty vector-expressing, nucleofected CD4 T cells two days later. Results were normalized with the fusion level of empty vector-expressing cells. Mean ± s.e.m. is shown (n=4). Asterisks indicate significance by two-tailed paired *t*-test. **c,** M2 were pre-treated with Alemtuzumab or an isotype control antibody and then co-cultured with autologous CD4 T cells. Subsequently, CD4 T cells were sorted and the percentage of cells positive for surface-exposed CD32 (left panel) and HLA-DR (right panel) was quantified by flow cytometry. In parallel, the cells were used in the HIV-1 fusion assay shown in Fig. 4i. Mean ± s.e.m. is shown (n=6-7). Asterisks indicate statistical significance by two-way ANOVA. *P* values were corrected for multiple comparison (Tukey). **P* ≤0.05; ***P* ≤0.01; ****P* ≤0.001.

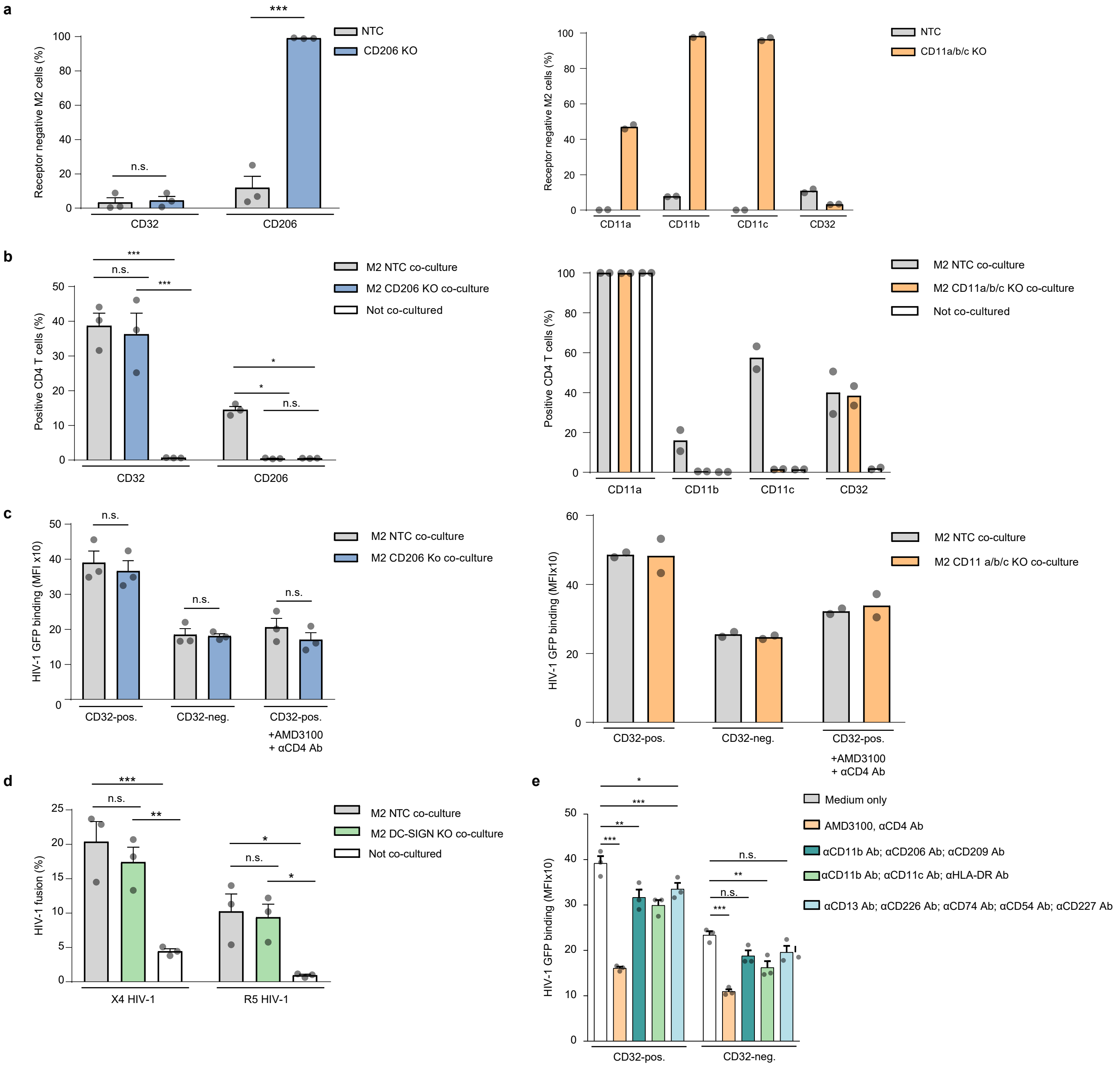

Figure S16 [Diminishing expression of previously reported HIV-1 binding receptors on macrophages does not impact HIV-1 attachment to co-cultured CD32+ autologous CD4 T cells.], Related to Figure 5. a, Monocytes were isolated from PBMC as described in Fig. S3b. and CD206 (left panel) or CD11a, b, and c (right panel) were knocked out (KO) by delivering Cas9 RNPs by nucleofection. The resulting monocytes were differentiated to M2 macrophages and stained for CD32 together with either CD206 or CD11a, b, and c, and the percentage of receptor-neg. cells was determined by flow cytometry. NTC was used as control. Mean ± s.e.m. are shown (n=3). Asterisks indicate statistical significance by two-way ANOVA. P values were corrected for multiple comparison (Šídák). **b**, Autologous CD4 T cells were co-cultured with M2 macrophages in (a). The expression of CD32 and CD206 (left panel), or CD32 and CD11a, b, and c (right panel) on co-cultured CD4 T cells was assessed by flow cytometry. Not co-cultured CD4 T cells served as control. Mean ± s.e.m. (n=3) (for CD206) and mean of two independent donors (CD11a/b/c) are shown. Asterisks indicate statistical significance by two-way ANOVA. P values were corrected for multiple comparison (Tukey). **c,** HIV-1 Vpr-GFP binding assay was performed with sorted CD4 T cells after co-culture with either M2 NTC, M2 CD206 KO (left panel) or M2 CD11a/b/c KO cells (right panel), followed by staining for CD32. HIV-1 Vpr-GFP binding to CD32+ and CD32- CD4 T cells was assessed by flow cytometry and is shown as the MFI of GFP. Mean ± s.e.m. (n=3) (CD206) and mean of two independent donors (CD11a/b/c) are shown. Statistical significance of the two-way ANOVA is indicated. P values were corrected for multiple comparison (Šídák). **d**, DC-SIGN was knocked out (KO) in monocytes by delivering Cas9 RNPs by nucleofection. NTC was used as control. Autologous CD4 T cells were subsequently co-cultured with M2 NTC or M2 DC-SIGN KO cells. After 2 days, co-cultured CD4 T cells were sorted and used in an HIV-1 fusion assay with either X4 or R5 HIV-1. Not co-cultured CD4 T cells served as control. Mean ± s.e.m. is shown (n=3). Asterisks indicate statistical significance by two-way ANOVA. P values were corrected for multiple comparison (Tukey). **e,** HIV-1 Vpr-GFP binding to M2 macrophage-co-cultured and sorted CD4 T cells following addition of the indicated antibodies against receptors known to be highly transferred to CD4 T cells during autologous M2 co-culture (see Fig. 1a) or the CXCR4 antagonist AMD3100. Mean ± s.e.m. are shown (n=3). Asterisks indicate statistical significance by two-way ANOVA. P values were corrected for multiple comparison (Šídák).

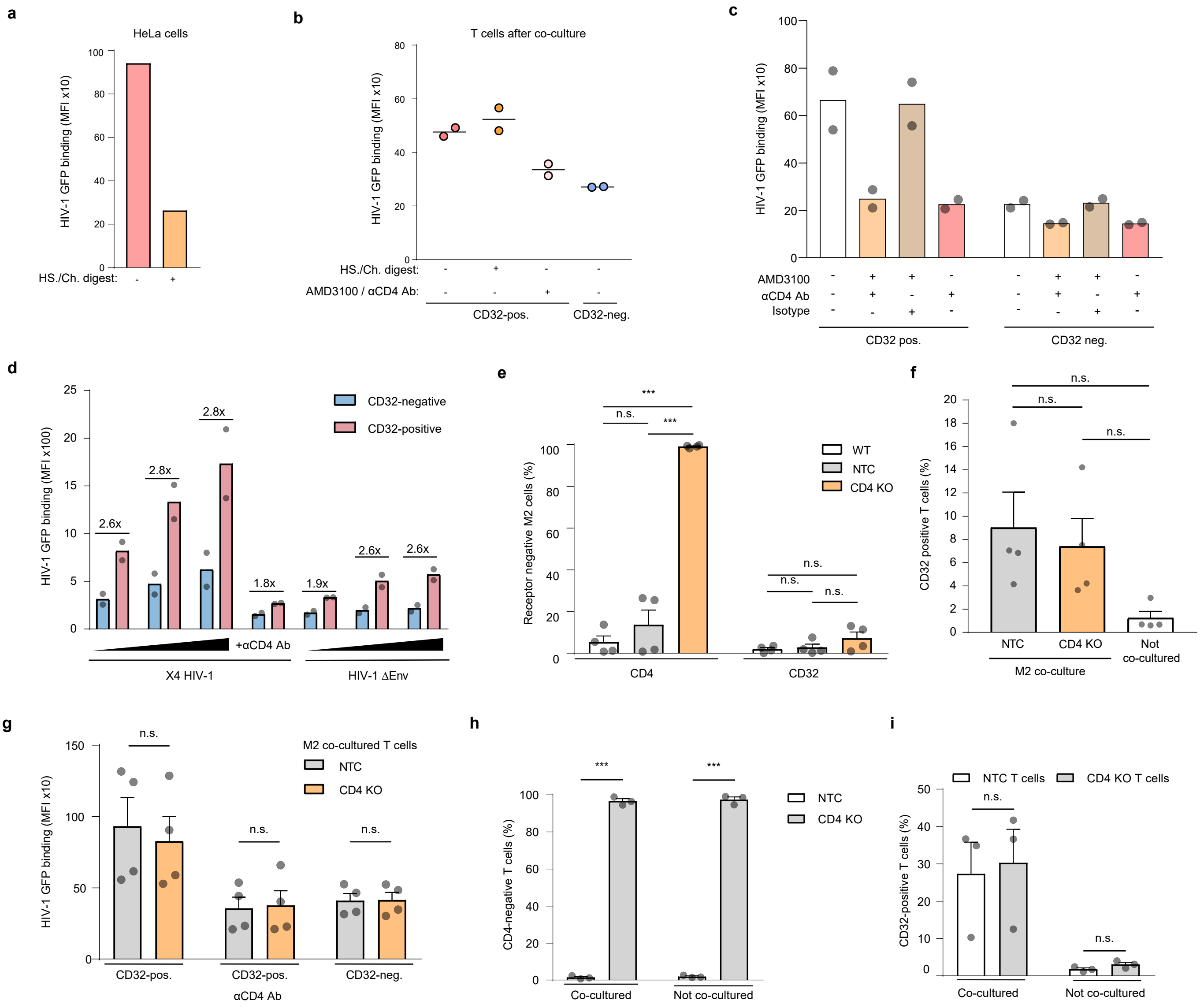

Figure S17 [Anti-CD4 antibodies effectively reduce the enhanced HIV-1 binding to CD32+ T cells.], Related to Figure 5. a, b, Heparinase I/II/III (HS.) and chondroitinase ABC (Ch.) digestion was applied to HeLa cells (a) or on CD4 T cells (b). The latter were sorted after co-cultured with M2 macrophages. These HeLa cells (a) or CD4 T cells (b) were then used in an HIV-1 Vpr-GFP binding assay. (b) After HIV-1 Vpr-GFP binding, CD4 T cells were stained with CD32, and the MFI of GFP on CD32-positive and negative populations is shown. Mean of two independent donors is shown. *P ≤0.05; **P ≤0.01; ***P ≤0.001; n.s.: not significant. **c,d,** HIV-1 Vpr-GFP binding assay was performed with CD4 T cells, which were sorted after co-cultured with M2. After incubation with virions, CD4 T cells were stained for CD32 and the HIV-1 Vpr-GFP binding level on CD32-positive and negative cell populations was assessed by flow cytometry and is shown as the MFI of the GFP signal. c, HIV-1 Vpr-GFP was incubated with CD4 T cells, in the presence of either anti-CD4 antibodies, an isotype control and or the CXCR4 antagonist AMD3100 in different combinations. d, Increasing amounts of X4 HIV-1 or X4 HIV-1ΔEnv particles carrying Vpr-GFP were incubated with CD4 T cells. Mean ± s.e.m. are shown (n=2-3). Asterisks indicate statistical significance by two-way ANOVA. P values were corrected for multiple comparison (Šídák). **e-g**, CD4 KO was introduced to monocytes isolated from PBMC by delivering Cas9 RNPs by nucleofection, and the resulting cells were differentiated to M2 macrophages as described in Fig. S3b. NTC was used as control. (e), The expression of CD32 and CD4 on macrophages was quantified by flow cytometry. Mean ± s.e.m. are shown (n=4). Asterisks indicate statistical significance by two-way ANOVA. P values were corrected for multiple comparison (Šídák). (f) CD4 T cells were co-cultured with M2 macrophages, and the transfer of CD32 was assessed by flow cytometry. Not co-cultured CD4 T cells served as control. Mean ± s.e.m. are shown (n=4). Statistical significance of the two-way ANOVA is indicated. P values were corrected for multiple comparison (Tukey). (g) CD4 T cells in (f) were sorted after co-culture, and subsequently incubated with HIV-1 Vpr-GFP, in the presence or absence of anti-CD4 monoclonal antibodies. After staining for CD32, the binding levels of HIV-1 Vpr-GFP on CD32-pos. and CD32-neg. T-cell populations were assessed by flow cytometry and are presented as the MFI of GFP. Mean ± s.e.m. are shown (n=4). Statistical significance of the two-way ANOVA is indicated. P values were corrected for multiple comparison (Šídák). **h, i**, CD4 T cells were isolated from PBMC and CD4 was knocked out (KO) by delivering Cas9 RNP by nucleofection. NTC was used as control. The resulting T cells were kept in culture for 16 days in the presence of IL-7/IL-15 before starting a 48-hour co-culture with autologous wild-type (WT) M2 macrophages. After co-culture, CD4 T cells were sorted and the HIV-1 binding assay was performed, followed by staining and assessing the surface levels of CD4 (h) and CD32 (i) by flow cytometry. Mean ± s.e.m. are shown (n=3). Asterisks indicate statistical significance by two-way ANOVA. P values were corrected for multiple comparison (Šídák). *P ≤0.05; **P ≤0.01; ***P ≤0.001; n.s.: not significant.

Table S2. [Information on ART-treated HIV-1 patients (CHI, ART) reported in this study], Related to Figure 3.

pseudo ID	source	viral load (cp/ml)	CD4 count (cells/µl)	CD4%	ART-Status	ART-initiation year	age	sex
L1	LMU	<50	595	30	yes	2012	44	male
L2	LMU	<50	1,127	42	yes	2001	58	male
L3	LMU	<50	544	38	yes	2016	25	female
L4	LMU	<50	406	20	yes	1994	71	male
L5	LMU	<50	938	37	yes	2011	36	male
L6	LMU	<50	968	35	yes	2013	63	male
L7	LMU	<50	405	25	yes	2009	46	male
L8	LMU	<50	947	42	yes	2001	61	male
L9	LMU	<50	619	33	yes	2009	55	male
L10	LMU	<50	575	30	yes	2001	43	male
L11	LMU	<50	662	32	yes	2010	47	male
L12	LMU	<50	471	35	yes	2012	47	male
L13	LMU	<50	575	32	yes	2007	42	male
L14	LMU	<50	685	38	yes	2014	32	female
L15	LMU	<50	560	33	yes	2009	52	male
L16	LMU	<50	834	31	yes	2011	41	male
L18	LMU	<50	449	35	yes	2020	48	male
L19	LMU	<50	585	33	yes	2010	60	male
L20	LMU	<50	856	34	yes	2011	60	male
L21	LMU	<50	581	44	yes	2000	57	male
L22	LMU	<50	591	33	yes	2004	59	male
L23	LMU	<50	394	43	yes	2008	79	male
L24	LMU	<50	528	32	yes	2014	64	male
L25	LMU	<50	448	39	yes	2003	73	male
L26	LMU	<50	460	29	yes	2010	42	male
L27	LMU	<50	411	46	yes	2011	53	male
T1	TUM	<40	865	36	yes	2014	40	male
T2	TUM	<40	1,059	29	yes	2018	43	male
T3	TUM	<40	853	34	yes	2016	29	male
T4	TUM	<40	599	32	yes	2011	52	male
T5	TUM	<40	578	28	yes	2004	39	female
T6	TUM	<40	233	37	yes	2004	56	male
T7	TUM	125	429	20	yes	2020	75	male
T8	TUM	108	610	39	yes	2021	43	male
T9	TUM	<40	806	35	yes	2003	54	male
T10	TUM	<40	751	36	yes	2009	51	male
T11	TUM	<40	638	39	yes	2014	34	male
T12	TUM	<40	823	31	yes	2009	55	female
T13	TUM	<40	940	34	yes	2001	50	male
T14	TUM	<40	927	30	yes	2020	23	male

Ludwig Maximilian University of Munich (LMU); Technical University of Munich (TUM); copies/ml (cp/ml); antiretroviral therapy (ART)

Table S3. [Information on untreated HIV-1 patient (CHI, No ART) reported in this study], Related to Figure 3.

Pseudo ID	Infection status	ART status	Viral load (cp/ml)
6	HIV	none	99,000
7	HIV	none	94,000
8	HIV	none	1,400,000
9	HIV	none	7,900,000
10	HIV	none	10,000
21	HIV	none	160,000
22	HIV	none	120,000
23	HIV	none	41,000
24	HIV	none	3,500,000
25	HIV	none	350,000
26	HIV	none	240,000
27	HIV	none	55,000
28	HIV	none	260,000
29	HIV	none	3,000
30	HIV	none	23,000

ART: Antiretroviral therapy; cp/ml: copies per ml plasma

Legend to Supplemental Videos

Video S1. [Time-lapse 3D-reconstruction of a live-cell imaging showing the transfer of CD32B-GFP.], Related to Figure 2. 293T cells transiently expressing CD32B-GFP were co-cultured with mCherry-expressing SupT1 cells in the presence of trogocytosis-enhancing bNAb PGT151. Live-cell imaging was taken with a spinning disc microscope for 4 hours. Four distinct transfer events between 293T cells and SupT1 cells were observed and annotated in the video. Individual frames from this video are shown in Fig. 2g. The time stamp is shown in the upper right corner. The white squares in the background are 10 µm x 10 µm and serve as a scale bar.

Video S2. [Detailed view of the first transfer event from video S1.], Related to Figure 2. The video is zoomed, slowed down, annotated and with a tilted viewing angle to better appreciate the transfer events. White arrows point to the CD32B-GFP^{+} membrane patches that are transferred to the SupT1 T cells. The white squares in the background are 10 µm x 10 µm and serve as a scale bar.
